# Supplementary material for: Mutations in SKI in Shprintzen–Goldberg syndrome lead to attenuated TGF-β responses through SKI stabilization
Source: eLife. 2021 Jan 8;10:e63545. doi: 10.7554/eLife.63545 (PMC7834018; doi:10.7554/eLife.63545)
Supplement: Figure 1—source data 1. [file elife-63545-fig1-data1.docx]

**Figure 1 – Source data 1 – Gori *et al.***

**Sequences of all knockout clones**

In each case, the wild-type sequence is given on top and the mutant sequence for a particular allele is given underneath. Depending on the gene, we found between two and 4 alleles for each gene. Sequences in lower case represent mismatches to the reference sequence.

**HEK293T SMAD4 KO clone 1**

*Allele 1*

^379^TGTGTGAATCCATATCACTACGAACGAGTTGTATCACCTGGAATTGAT^426^

TGTGTGAATCCATAT-ACTACGAACGAGTTGTATCACCTGGAATTGAT

Allele 2

^379^TGTGTGAATCCATATCACTACGAACGAGTTGTATCACCTGGAATTGAT^426^

TGTGTGAATCCATAT----ACGAACGAGTTGTATCACCTGGAATTGAT

Allele 3

^379^TGTGTGAATCCATATCACTACGAACGAGTTGTATCACCTGGAATTGAT^426^

TGTGTGAATCCATATC-CTACGAACGAGTTGTATCACCTGGAATTGAT

**HEK293T SMAD4 KO clone 2**

*Allele 1*

^379^TGTGTGAATCCATATCACTACGAACGAGTTGTATCACCTGGAATTGAT^426^

TGTGTGAATCC----CACTACGAACGAGTTGTATCACCTGGAATTGAT

*Allele 2* and *3* not detected

**HEK293T SMAD2 KO clone 1**

*Allele 1*

^799^ACTTACTCAGAACCTGCATTTTGGTGTTCGATAGCATATTATGAATTAAAT^849^

ACTTACTCAGAACCTGCATT--GGTGTTCGATAGCATATTATGAATTAAAT

*Allele 2* not detected

**HEK293T SMAD2 KO clone 2**

*Allele 1*

^799^ACTTACTCAGAACCTGCATTTTGGTGTTCGATAGCATATTATGAATTAAAT^849^

ACTTACTCAGAACCTGCATT--GGTGTTCGATAGCATATTATGAATTAAAT

*Allele 2*

^799^ACTTACTCAGAACCTGCATTTTGGTGTTCGATAGCATATTATGAATTAAAT^849^

ACTTACTCAGAACCTGCA >400 bp insertion

**HEK293T SMAD3 KO clone 1**

*Allele 1*

^703^ATCTCCTACTACGAGCTGAACCAGCG-CGTCGGGGAGACATTCCACGCC^750^

ATCTCCTACTACGAGCTGAACCAGCGaCGTCGGGGAGACATTCCACGCC

*Allele 2*

^703^ATCTCCTACTACGAGCTGAACCAGCGCGTCGGGGAGACATTCCACGCCTC^752^

ATCTCCTACTACGAGCTGAACCAGCG--TCGGGGAGACATTCCACGCCTC

*Allele 3* not detected

**HEK293T SMAD3 KO clone 2**

*Allele 1*

^703^ATCTCCTACTACGAGCTGAACCAGCGCGTCGGGGAGACATTCCACGCC^750^

ATCTCCTACTACGAGCTGAACCAGC------------CATTCCACGCC

*Allele 2*

^703^ATCTCCTACTACGAGCTGAACCAGCGCGTCGGGGAGACATTCCACGCC^750^

--------------80 bp deletion----------ATTCCACGCC

*Allele 3*

^703^ATCTCCTACTACGAGCTGAACCAGCGCGTCGGGGAGACATTCCACGCC^750^

--------------72 bp deletion------TTCCACGCC

**HEK293T SMAD2/3 double knockout clone 1**

***SMAD2***

*Allele 1*

^799^ACTTACTCAGAACCTGCATTTT-GGTGTTCGATAGCATATTATGAATTAAAT^849^

ACTTACTCAGAACCTGCATTTTTGGTGTTCGATAGCATATTATGAATTAAAT

*Allele 2* and *3* not detected

***SMAD3***

*Allele 1*

^703^ATCTCCTACTACGAGCTGAACCAGCGCGTCGGGGAGACATTCCACGCCTCGC^754^

ATCTCCTACTACGAGCT------------CGGGGAGACATTCCACGCCTCGC

*Allele 2*

^703^ATCTCCTACTACGAGCTGAACCAGCGCGTCGGGGAGACATTCCACGCCTCGC^754^

ATCTCCTACTACGAG---------------------ACATTCCACGCCTCGC

*Allele 3*

^703^ATCTCCTACTACGAGCTGAACCAGCGCGTCGGGGAGACATTCCACGCCTCGC^754^

ATCTCCTACTACGAGCTGAACCAGCG--TCGGGGAGACATTCCACGCCTCGC

**HEK293T SMAD2/3 double knockout clone 2**

***SMAD2***

*Allele 1*

^799^ACTTACTCAGAACCTGCATTTTGGTGTTCGATAGCATATTATGAATTAAAT^849^

ACTTACTCAGAACCTGCATT--GGTGTTCGATAGCATATTATGAATTAAAT

*Allele 2*

^799^ACTTACTCAGAACCTGCATTTT-GGTGTTCGATAGCATATTATGAATTAAAT^849^

ACTTACTCAGAACCTGCATTTTTGGTGTTCGATAGCATATTATGAATTAAAT

*Allele 3*

^799^ACTTACTCAGAACCTGCATTTTGGTGTTCGATAGCATATTATGAATTAAAT^849^

ACTTACTCAGAACCTG--TTTTGGTGTTCGATAGCATATTATGAATTAAAT

***SMAD3***

*Allele 1*

^703^ATCTCCTACTACGAGCTGAACCAGCGCGTCGGGGAGACATTCCACGCCTCGC^754^

ATCTCCTACTACGAGCTGAACCAGCG------GGAGACATTCCACGCCTCGC

*Allele 2*

^703^ATCTCCTACTACGAGCTGAACCAGCGCGTCGGGGAGACATTCCACGCCTCGC^754^

ATCTCCTACTACGAGCTGAACCAGCGC---------------CACGCCTCGC

*Allele 3*

^703^ATCTCCTACTACGAGCTGAACCAGCGCGTCGGGGAGACATTCCACGCCTCGC^754^

ATCTCCTACTACGAGCTGAACCAGCG--TCGGGGAGACATTCCACGCCTCGC

**HaCaT SMAD4 KO clone 1**

*Allele 1*

^379^TGTGTGAATCCATATCACTACGAACGAGTTGTATCACCTGGAATTGAT^426^

TGTGTGAATCCATAT-------AACGAGTTGTATCACCTGGAATTGAT

*Allele 2*

^379^TGTGTGAATCCATATCACTACGAACGAGTTGTATCACCTGGAATTGAT^426^

TGTGTGAATCCATAT-insertion 34-bp

*Allele 3*

^379^TGTGTGAATCCATATCACTACGAACGAGTTGTATCACCTGGAATTGAT^426^

TGTGTGAATCCATAT----ACGAACGAGTTGTATCACCTGGAATTGAT

*Allele 4*

^379^TGTGTGAATCCATATCACTACGAACGAGTTGTATCACCTGGAATTGAT^426^

TGTGTGAATCCATAT-insertion 158 bp

**HaCaT SMAD4 KO clone 2**

*Allele 1*

^379^TGTGTGAATCCATATCACTACGAACGAGTTGTATCACCTGGAATTGAT^426^

TGTGTGAATCCATATC--TACGAACGAGTTGTATCACCTGGAATTGAT

*Allele 2*

^379^TGTGTGAATCCATATCACTACGAACGAGTTGTATCACCTGGAATTGAT^426^

TGTGTGAATCCATAT-ACTACGAACGAGTTGTATCACCTGGAATTGAT

*Allele 3*

^379^TGTGTGAATCCATAT-CACTACGAACGAGTTGTATCACCTGGAATTGAT^426^

TGTGTGAATCCATATACACTACGAACGAGTTGTATCACCTGGAATTGAT

*Allele 4* not detected

**HaCaT SMAD4 KO clone 3**

*Allele 1*

^1474^GTTGATGACCTTCGTCGCTTATGCATACTCAGGATGAGTTTTGTGAAA^1521^

GTTGATGACCTTCG--GCTTATGCATACTCAGGATGAGTTTTGTGAAA

*Allele 2*

^1474^GTTGATGACCTTCGTCGCTTATGCATACTCAGGATGAGTTTTGTGAAA^1521^

GTTGATGACCTT--TCGCTTATGCATACTCAGGATGAGTTTTGTGAAA

*Allele 3*

^1474^GTTGATGACCTTCGT-CGCTTATGCATACTCAGGATGAGTTTTGTGAAA^1521^

GTTGATGACCTTCGTTCGCTTATGCATACTCAGGATGAGTTTTGTGAAA

*Allele 4* not detected

**HaCaT SMAD4 KO clone 4**

*Allele 1*

^1474^GTTGATGACCTTCGTCGCTTATGCATACTCAGGATGAGTTTTGTGAAA^1521^

-------------------TATGCATACTCAGGATGAGTTTTGTGAAA

*Allele 2*

^1474^GTTGATGACCTTCGTCGCTTATGCATACTCAGGATGAGTTTTGTGAAA^1521^

GTTGATGACCTTCG---CTTATGCATACTCAGGATGAGTTTTGTGAAA

*Allele 3*

^1474^GTTGATGACCTTCGTCGCTTATGCATACTCAGGATGAGTTTTGTGAAA^1521^

GTTGATGACCTTCG-insertion 211 bp

*Allele 4* not detected
