# Supplementary material for: Mutations in SKI in Shprintzen–Goldberg syndrome lead to attenuated TGF-β responses through SKI stabilization
Source: eLife. 2021 Jan 8;10:e63545. doi: 10.7554/eLife.63545 (PMC7834018; doi:10.7554/eLife.63545)

```
26/11/2015 - 10:22:19
peptides generated from sequence-file :
 E:\Multipep Spotter09CR-UK\Users\Caroline Hill\Ski 19 mutation.SEQ

Nr.      Pos.     Mol.Weight       Sequence Label
1        A 1      3800.5   F-Q-P-H-P-G-A-Q-K-T-L-E-Q-F-H-L-S-S-M-S-S-
L-G-G-P-A-A-F-S-A-R-W-A-Q-E
2        A 2      3885.6   F-Q-P-H-P-G-R-Q-K-T-L-E-Q-F-H-L-S-S-M-S-S-
L-G-G-P-A-A-F-S-A-R-W-A-Q-E
3        A 3      3843.5   F-Q-P-H-P-G-N-Q-K-T-L-E-Q-F-H-L-S-S-M-S-S-
L-G-G-P-A-A-F-S-A-R-W-A-Q-E
4        A 4      3844.5   F-Q-P-H-P-G-D-Q-K-T-L-E-Q-F-H-L-S-S-M-S-S-
L-G-G-P-A-A-F-S-A-R-W-A-Q-E
5        A 5      3832.5   F-Q-P-H-P-G-C-Q-K-T-L-E-Q-F-H-L-S-S-M-S-S-
L-G-G-P-A-A-F-S-A-R-W-A-Q-E
6        A 6      3857.5   F-Q-P-H-P-G-Q-Q-K-T-L-E-Q-F-H-L-S-S-M-S-S-
L-G-G-P-A-A-F-S-A-R-W-A-Q-E
7        A 7      3858.5   F-Q-P-H-P-G-E-Q-K-T-L-E-Q-F-H-L-S-S-M-S-S-
L-G-G-P-A-A-F-S-A-R-W-A-Q-E
8        A 8      3786.5   F-Q-P-H-P-G-G-Q-K-T-L-E-Q-F-H-L-S-S-M-S-S-
L-G-G-P-A-A-F-S-A-R-W-A-Q-E
9        A 9      3866.5   F-Q-P-H-P-G-H-Q-K-T-L-E-Q-F-H-L-S-S-M-S-S-
L-G-G-P-A-A-F-S-A-R-W-A-Q-E
10       A10      3842.6   F-Q-P-H-P-G-I-Q-K-T-L-E-Q-F-H-L-S-S-M-S-S-
L-G-G-P-A-A-F-S-A-R-W-A-Q-E
11       A11      3842.6   F-Q-P-H-P-G-L-Q-K-T-L-E-Q-F-H-L-S-S-M-S-S-
L-G-G-P-A-A-F-S-A-R-W-A-Q-E
12       A12      3857.6   F-Q-P-H-P-G-K-Q-K-T-L-E-Q-F-H-L-S-S-M-S-S-
L-G-G-P-A-A-F-S-A-R-W-A-Q-E
13       A13      3860.6   F-Q-P-H-P-G-M-Q-K-T-L-E-Q-F-H-L-S-S-M-S-S-
L-G-G-P-A-A-F-S-A-R-W-A-Q-E
14       A14      3876.6   F-Q-P-H-P-G-F-Q-K-T-L-E-Q-F-H-L-S-S-M-S-S-
L-G-G-P-A-A-F-S-A-R-W-A-Q-E
15       A15      3826.5   F-Q-P-H-P-G-P-Q-K-T-L-E-Q-F-H-L-S-S-M-S-S-
L-G-G-P-A-A-F-S-A-R-W-A-Q-E
16       A16      3816.5   F-Q-P-H-P-G-S-Q-K-T-L-E-Q-F-H-L-S-S-M-S-S-
L-G-G-P-A-A-F-S-A-R-W-A-Q-E
17       A17      3830.5   F-Q-P-H-P-G-T-Q-K-T-L-E-Q-F-H-L-S-S-M-S-S-
L-G-G-P-A-A-F-S-A-R-W-A-Q-E
18       A18      3915.6   F-Q-P-H-P-G-W-Q-K-T-L-E-Q-F-H-L-S-S-M-S-S-
L-G-G-P-A-A-F-S-A-R-W-A-Q-E
19       A19      3892.6   F-Q-P-H-P-G-Y-Q-K-T-L-E-Q-F-H-L-S-S-M-S-S-
L-G-G-P-A-A-F-S-A-R-W-A-Q-E
20       A20      3828.5   F-Q-P-H-P-G-V-Q-K-T-L-E-Q-F-H-L-S-S-M-S-S-
L-G-G-P-A-A-F-S-A-R-W-A-Q-E
21       B 1      3785.6   F-Q-P-H-P-G-L-A-K-T-L-E-Q-F-H-L-S-S-M-S-S-
L-G-G-P-A-A-F-S-A-R-W-A-Q-E
22       B 2      3870.7   F-Q-P-H-P-G-L-R-K-T-L-E-Q-F-H-L-S-S-M-S-S-
L-G-G-P-A-A-F-S-A-R-W-A-Q-E
23       B 3      3828.6   F-Q-P-H-P-G-L-N-K-T-L-E-Q-F-H-L-S-S-M-S-S-
L-G-G-P-A-A-F-S-A-R-W-A-Q-E
24       B 4      3829.6   F-Q-P-H-P-G-L-D-K-T-L-E-Q-F-H-L-S-S-M-S-S-
L-G-G-P-A-A-F-S-A-R-W-A-Q-E
25       B 5      3817.6   F-Q-P-H-P-G-L-C-K-T-L-E-Q-F-H-L-S-S-M-S-S-
```

L-G-G-P-A-A-F-S-A-R-W-A-Q-E
26 B 6 3842.6 F-Q-P-H-P-G-L-Q-K-T-L-E-Q-F-H-L-S-S-M-S-S-
L-G-G-P-A-A-F-S-A-R-W-A-Q-E
27 B 7 3843.6 F-Q-P-H-P-G-L-E-K-T-L-E-Q-F-H-L-S-S-M-S-S-
L-G-G-P-A-A-F-S-A-R-W-A-Q-E
28 B 8 3771.6 F-Q-P-H-P-G-L-G-K-T-L-E-Q-F-H-L-S-S-M-S-S-
L-G-G-P-A-A-F-S-A-R-W-A-Q-E
29 B 9 3851.6 F-Q-P-H-P-G-L-H-K-T-L-E-Q-F-H-L-S-S-M-S-S-
L-G-G-P-A-A-F-S-A-R-W-A-Q-E
30 B10 3827.7 F-Q-P-H-P-G-L-I-K-T-L-E-Q-F-H-L-S-S-M-S-S-
L-G-G-P-A-A-F-S-A-R-W-A-Q-E
31 B11 3827.7 F-Q-P-H-P-G-L-L-K-T-L-E-Q-F-H-L-S-S-M-S-S-
L-G-G-P-A-A-F-S-A-R-W-A-Q-E
32 B12 3842.7 F-Q-P-H-P-G-L-K-K-T-L-E-Q-F-H-L-S-S-M-S-S-
L-G-G-P-A-A-F-S-A-R-W-A-Q-E
33 B13 3845.7 F-Q-P-H-P-G-L-M-K-T-L-E-Q-F-H-L-S-S-M-S-S-
L-G-G-P-A-A-F-S-A-R-W-A-Q-E
34 B14 3861.7 F-Q-P-H-P-G-L-F-K-T-L-E-Q-F-H-L-S-S-M-S-S-
L-G-G-P-A-A-F-S-A-R-W-A-Q-E
35 B15 3811.6 F-Q-P-H-P-G-L-P-K-T-L-E-Q-F-H-L-S-S-M-S-S-
L-G-G-P-A-A-F-S-A-R-W-A-Q-E
36 B16 3801.6 F-Q-P-H-P-G-L-S-K-T-L-E-Q-F-H-L-S-S-M-S-S-
L-G-G-P-A-A-F-S-A-R-W-A-Q-E
37 B17 3815.6 F-Q-P-H-P-G-L-T-K-T-L-E-Q-F-H-L-S-S-M-S-S-
L-G-G-P-A-A-F-S-A-R-W-A-Q-E
38 B18 3900.7 F-Q-P-H-P-G-L-W-K-T-L-E-Q-F-H-L-S-S-M-S-S-
L-G-G-P-A-A-F-S-A-R-W-A-Q-E
39 B19 3877.7 F-Q-P-H-P-G-L-Y-K-T-L-E-Q-F-H-L-S-S-M-S-S-
L-G-G-P-A-A-F-S-A-R-W-A-Q-E
40 B20 3813.6 F-Q-P-H-P-G-L-V-K-T-L-E-Q-F-H-L-S-S-M-S-S-
L-G-G-P-A-A-F-S-A-R-W-A-Q-E
41 C 1 3785.5 F-Q-P-H-P-G-L-Q-A-T-L-E-Q-F-H-L-S-S-M-S-S-
L-G-G-P-A-A-F-S-A-R-W-A-Q-E
42 C 2 3870.6 F-Q-P-H-P-G-L-Q-R-T-L-E-Q-F-H-L-S-S-M-S-S-
L-G-G-P-A-A-F-S-A-R-W-A-Q-E
43 C 3 3828.5 F-Q-P-H-P-G-L-Q-N-T-L-E-Q-F-H-L-S-S-M-S-S-
L-G-G-P-A-A-F-S-A-R-W-A-Q-E
44 C 4 3829.5 F-Q-P-H-P-G-L-Q-D-T-L-E-Q-F-H-L-S-S-M-S-S-
L-G-G-P-A-A-F-S-A-R-W-A-Q-E
45 C 5 3817.5 F-Q-P-H-P-G-L-Q-C-T-L-E-Q-F-H-L-S-S-M-S-S-
L-G-G-P-A-A-F-S-A-R-W-A-Q-E
46 C 6 3842.5 F-Q-P-H-P-G-L-Q-Q-T-L-E-Q-F-H-L-S-S-M-S-S-
L-G-G-P-A-A-F-S-A-R-W-A-Q-E
47 C 7 3843.5 F-Q-P-H-P-G-L-Q-E-T-L-E-Q-F-H-L-S-S-M-S-S-
L-G-G-P-A-A-F-S-A-R-W-A-Q-E
48 C 8 3771.5 F-Q-P-H-P-G-L-Q-G-T-L-E-Q-F-H-L-S-S-M-S-S-
L-G-G-P-A-A-F-S-A-R-W-A-Q-E
49 C 9 3851.5 F-Q-P-H-P-G-L-Q-H-T-L-E-Q-F-H-L-S-S-M-S-S-
L-G-G-P-A-A-F-S-A-R-W-A-Q-E
50 C10 3827.6 F-Q-P-H-P-G-L-Q-I-T-L-E-Q-F-H-L-S-S-M-S-S-
L-G-G-P-A-A-F-S-A-R-W-A-Q-E
51 C11 3827.6 F-Q-P-H-P-G-L-Q-L-T-L-E-Q-F-H-L-S-S-M-S-S-
L-G-G-P-A-A-F-S-A-R-W-A-Q-E
52 C12 3842.6 F-Q-P-H-P-G-L-Q-K-T-L-E-Q-F-H-L-S-S-M-S-S-

			L-G-G-P-A-A-F-S-A-R-W-A-Q-E
53	C13	3845.6	F-Q-P-H-P-G-L-Q-M-T-L-E-Q-F-H-L-S-S-M-S-S-L-G-G-P-A-A-F-S-A-R-W-A-Q-E
54	C14	3861.6	F-Q-P-H-P-G-L-Q-F-T-L-E-Q-F-H-L-S-S-M-S-S-L-G-G-P-A-A-F-S-A-R-W-A-Q-E
55	C15	3811.5	F-Q-P-H-P-G-L-Q-P-T-L-E-Q-F-H-L-S-S-M-S-S-L-G-G-P-A-A-F-S-A-R-W-A-Q-E
56	C16	3801.5	F-Q-P-H-P-G-L-Q-S-T-L-E-Q-F-H-L-S-S-M-S-S-L-G-G-P-A-A-F-S-A-R-W-A-Q-E
57	C17	3815.5	F-Q-P-H-P-G-L-Q-T-T-L-E-Q-F-H-L-S-S-M-S-S-L-G-G-P-A-A-F-S-A-R-W-A-Q-E
58	C18	3900.6	F-Q-P-H-P-G-L-Q-W-T-L-E-Q-F-H-L-S-S-M-S-S-L-G-G-P-A-A-F-S-A-R-W-A-Q-E
59	C19	3877.6	F-Q-P-H-P-G-L-Q-Y-T-L-E-Q-F-H-L-S-S-M-S-S-L-G-G-P-A-A-F-S-A-R-W-A-Q-E
60	C20	3813.5	F-Q-P-H-P-G-L-Q-V-T-L-E-Q-F-H-L-S-S-M-S-S-L-G-G-P-A-A-F-S-A-R-W-A-Q-E
61	D 1	3812.6	F-Q-P-H-P-G-L-Q-K-A-L-E-Q-F-H-L-S-S-M-S-S-L-G-G-P-A-A-F-S-A-R-W-A-Q-E
62	D 2	3897.7	F-Q-P-H-P-G-L-Q-K-R-L-E-Q-F-H-L-S-S-M-S-S-L-G-G-P-A-A-F-S-A-R-W-A-Q-E
63	D 3	3855.6	F-Q-P-H-P-G-L-Q-K-N-L-E-Q-F-H-L-S-S-M-S-S-L-G-G-P-A-A-F-S-A-R-W-A-Q-E
64	D 4	3856.6	F-Q-P-H-P-G-L-Q-K-D-L-E-Q-F-H-L-S-S-M-S-S-L-G-G-P-A-A-F-S-A-R-W-A-Q-E
65	D 5	3844.6	F-Q-P-H-P-G-L-Q-K-C-L-E-Q-F-H-L-S-S-M-S-S-L-G-G-P-A-A-F-S-A-R-W-A-Q-E
66	D 6	3869.6	F-Q-P-H-P-G-L-Q-K-Q-L-E-Q-F-H-L-S-S-M-S-S-L-G-G-P-A-A-F-S-A-R-W-A-Q-E
67	D 7	3870.6	F-Q-P-H-P-G-L-Q-K-E-L-E-Q-F-H-L-S-S-M-S-S-L-G-G-P-A-A-F-S-A-R-W-A-Q-E
68	D 8	3798.6	F-Q-P-H-P-G-L-Q-K-G-L-E-Q-F-H-L-S-S-M-S-S-L-G-G-P-A-A-F-S-A-R-W-A-Q-E
69	D 9	3878.6	F-Q-P-H-P-G-L-Q-K-H-L-E-Q-F-H-L-S-S-M-S-S-L-G-G-P-A-A-F-S-A-R-W-A-Q-E
70	D10	3854.7	F-Q-P-H-P-G-L-Q-K-I-L-E-Q-F-H-L-S-S-M-S-S-L-G-G-P-A-A-F-S-A-R-W-A-Q-E
71	D11	3854.7	F-Q-P-H-P-G-L-Q-K-L-L-E-Q-F-H-L-S-S-M-S-S-L-G-G-P-A-A-F-S-A-R-W-A-Q-E
72	D12	3869.7	F-Q-P-H-P-G-L-Q-K-K-L-E-Q-F-H-L-S-S-M-S-S-L-G-G-P-A-A-F-S-A-R-W-A-Q-E
73	D13	3872.7	F-Q-P-H-P-G-L-Q-K-M-L-E-Q-F-H-L-S-S-M-S-S-L-G-G-P-A-A-F-S-A-R-W-A-Q-E
74	D14	3888.7	F-Q-P-H-P-G-L-Q-K-F-L-E-Q-F-H-L-S-S-M-S-S-L-G-G-P-A-A-F-S-A-R-W-A-Q-E
75	D15	3838.6	F-Q-P-H-P-G-L-Q-K-P-L-E-Q-F-H-L-S-S-M-S-S-L-G-G-P-A-A-F-S-A-R-W-A-Q-E
76	D16	3828.6	F-Q-P-H-P-G-L-Q-K-S-L-E-Q-F-H-L-S-S-M-S-S-L-G-G-P-A-A-F-S-A-R-W-A-Q-E
77	D17	3842.6	F-Q-P-H-P-G-L-Q-K-T-L-E-Q-F-H-L-S-S-M-S-S-L-G-G-P-A-A-F-S-A-R-W-A-Q-E
78	D18	3927.7	F-Q-P-H-P-G-L-Q-K-W-L-E-Q-F-H-L-S-S-M-S-S-L-G-G-P-A-A-F-S-A-R-W-A-Q-E
79	D19	3904.7	F-Q-P-H-P-G-L-Q-K-Y-L-E-Q-F-H-L-S-S-M-S-S-

L-G-G-P-A-A-F-S-A-R-W-A-Q-E
80 D20 3840.6 F-Q-P-H-P-G-L-Q-K-V-L-E-Q-F-H-L-S-S-M-S-S-L-G-G-P-A-A-F-S-A-R-W-A-Q-E
81 E 1 3800.5 F-Q-P-H-P-G-L-Q-K-T-A-E-Q-F-H-L-S-S-M-S-S-L-G-G-P-A-A-F-S-A-R-W-A-Q-E
82 E 2 3885.6 F-Q-P-H-P-G-L-Q-K-T-R-E-Q-F-H-L-S-S-M-S-S-L-G-G-P-A-A-F-S-A-R-W-A-Q-E
83 E 3 3843.5 F-Q-P-H-P-G-L-Q-K-T-N-E-Q-F-H-L-S-S-M-S-S-L-G-G-P-A-A-F-S-A-R-W-A-Q-E
84 E 4 3844.5 F-Q-P-H-P-G-L-Q-K-T-D-E-Q-F-H-L-S-S-M-S-S-L-G-G-P-A-A-F-S-A-R-W-A-Q-E
85 E 5 3832.5 F-Q-P-H-P-G-L-Q-K-T-C-E-Q-F-H-L-S-S-M-S-S-L-G-G-P-A-A-F-S-A-R-W-A-Q-E
86 E 6 3857.5 F-Q-P-H-P-G-L-Q-K-T-Q-E-Q-F-H-L-S-S-M-S-S-L-G-G-P-A-A-F-S-A-R-W-A-Q-E
87 E 7 3858.5 F-Q-P-H-P-G-L-Q-K-T-E-E-Q-F-H-L-S-S-M-S-S-L-G-G-P-A-A-F-S-A-R-W-A-Q-E
88 E 8 3786.5 F-Q-P-H-P-G-L-Q-K-T-G-E-Q-F-H-L-S-S-M-S-S-L-G-G-P-A-A-F-S-A-R-W-A-Q-E
89 E 9 3866.5 F-Q-P-H-P-G-L-Q-K-T-H-E-Q-F-H-L-S-S-M-S-S-L-G-G-P-A-A-F-S-A-R-W-A-Q-E
90 E10 3842.6 F-Q-P-H-P-G-L-Q-K-T-I-E-Q-F-H-L-S-S-M-S-S-L-G-G-P-A-A-F-S-A-R-W-A-Q-E
91 E11 3842.6 F-Q-P-H-P-G-L-Q-K-T-L-E-Q-F-H-L-S-S-M-S-S-L-G-G-P-A-A-F-S-A-R-W-A-Q-E
92 E12 3857.6 F-Q-P-H-P-G-L-Q-K-T-K-E-Q-F-H-L-S-S-M-S-S-L-G-G-P-A-A-F-S-A-R-W-A-Q-E
93 E13 3860.6 F-Q-P-H-P-G-L-Q-K-T-M-E-Q-F-H-L-S-S-M-S-S-L-G-G-P-A-A-F-S-A-R-W-A-Q-E
94 E14 3876.6 F-Q-P-H-P-G-L-Q-K-T-F-E-Q-F-H-L-S-S-M-S-S-L-G-G-P-A-A-F-S-A-R-W-A-Q-E
95 E15 3826.5 F-Q-P-H-P-G-L-Q-K-T-P-E-Q-F-H-L-S-S-M-S-S-L-G-G-P-A-A-F-S-A-R-W-A-Q-E
96 E16 3816.5 F-Q-P-H-P-G-L-Q-K-T-S-E-Q-F-H-L-S-S-M-S-S-L-G-G-P-A-A-F-S-A-R-W-A-Q-E
97 E17 3830.5 F-Q-P-H-P-G-L-Q-K-T-T-E-Q-F-H-L-S-S-M-S-S-L-G-G-P-A-A-F-S-A-R-W-A-Q-E
98 E18 3915.6 F-Q-P-H-P-G-L-Q-K-T-W-E-Q-F-H-L-S-S-M-S-S-L-G-G-P-A-A-F-S-A-R-W-A-Q-E
99 E19 3892.6 F-Q-P-H-P-G-L-Q-K-T-Y-E-Q-F-H-L-S-S-M-S-S-L-G-G-P-A-A-F-S-A-R-W-A-Q-E
100 E20 3828.5 F-Q-P-H-P-G-L-Q-K-T-V-E-Q-F-H-L-S-S-M-S-S-L-G-G-P-A-A-F-S-A-R-W-A-Q-E
101 F 1 3784.6 F-Q-P-H-P-G-L-Q-K-T-L-A-Q-F-H-L-S-S-M-S-S-L-G-G-P-A-A-F-S-A-R-W-A-Q-E
102 F 2 3869.7 F-Q-P-H-P-G-L-Q-K-T-L-R-Q-F-H-L-S-S-M-S-S-L-G-G-P-A-A-F-S-A-R-W-A-Q-E
103 F 3 3827.6 F-Q-P-H-P-G-L-Q-K-T-L-N-Q-F-H-L-S-S-M-S-S-L-G-G-P-A-A-F-S-A-R-W-A-Q-E
104 F 4 3828.6 F-Q-P-H-P-G-L-Q-K-T-L-D-Q-F-H-L-S-S-M-S-S-L-G-G-P-A-A-F-S-A-R-W-A-Q-E
105 F 5 3816.6 F-Q-P-H-P-G-L-Q-K-T-L-C-Q-F-H-L-S-S-M-S-S-L-G-G-P-A-A-F-S-A-R-W-A-Q-E
106 F 6 3841.6 F-Q-P-H-P-G-L-Q-K-T-L-Q-Q-F-H-L-S-S-M-S-S-

L-G-G-P-A-A-F-S-A-R-W-A-Q-E
107 F 7 3842.6 F-Q-P-H-P-G-L-Q-K-T-L-E-Q-F-H-L-S-S-M-S-S-
L-G-G-P-A-A-F-S-A-R-W-A-Q-E
108 F 8 3770.6 F-Q-P-H-P-G-L-Q-K-T-L-G-Q-F-H-L-S-S-M-S-S-
L-G-G-P-A-A-F-S-A-R-W-A-Q-E
109 F 9 3850.6 F-Q-P-H-P-G-L-Q-K-T-L-H-Q-F-H-L-S-S-M-S-S-
L-G-G-P-A-A-F-S-A-R-W-A-Q-E
110 F10 3826.7 F-Q-P-H-P-G-L-Q-K-T-L-I-Q-F-H-L-S-S-M-S-S-
L-G-G-P-A-A-F-S-A-R-W-A-Q-E
111 F11 3826.7 F-Q-P-H-P-G-L-Q-K-T-L-L-Q-F-H-L-S-S-M-S-S-
L-G-G-P-A-A-F-S-A-R-W-A-Q-E
112 F12 3841.7 F-Q-P-H-P-G-L-Q-K-T-L-K-Q-F-H-L-S-S-M-S-S-
L-G-G-P-A-A-F-S-A-R-W-A-Q-E
113 F13 3844.7 F-Q-P-H-P-G-L-Q-K-T-L-M-Q-F-H-L-S-S-M-S-S-
L-G-G-P-A-A-F-S-A-R-W-A-Q-E
114 F14 3860.7 F-Q-P-H-P-G-L-Q-K-T-L-F-Q-F-H-L-S-S-M-S-S-
L-G-G-P-A-A-F-S-A-R-W-A-Q-E
115 F15 3810.6 F-Q-P-H-P-G-L-Q-K-T-L-P-Q-F-H-L-S-S-M-S-S-
L-G-G-P-A-A-F-S-A-R-W-A-Q-E
116 F16 3800.6 F-Q-P-H-P-G-L-Q-K-T-L-S-Q-F-H-L-S-S-M-S-S-
L-G-G-P-A-A-F-S-A-R-W-A-Q-E
117 F17 3814.6 F-Q-P-H-P-G-L-Q-K-T-L-T-Q-F-H-L-S-S-M-S-S-
L-G-G-P-A-A-F-S-A-R-W-A-Q-E
118 F18 3899.7 F-Q-P-H-P-G-L-Q-K-T-L-W-Q-F-H-L-S-S-M-S-S-
L-G-G-P-A-A-F-S-A-R-W-A-Q-E
119 F19 3876.7 F-Q-P-H-P-G-L-Q-K-T-L-Y-Q-F-H-L-S-S-M-S-S-
L-G-G-P-A-A-F-S-A-R-W-A-Q-E
120 F20 3812.6 F-Q-P-H-P-G-L-Q-K-T-L-V-Q-F-H-L-S-S-M-S-S-
L-G-G-P-A-A-F-S-A-R-W-A-Q-E
121 G 1 3785.6 F-Q-P-H-P-G-L-Q-K-T-L-E-A-F-H-L-S-S-M-S-S-
L-G-G-P-A-A-F-S-A-R-W-A-Q-E
122 G 2 3870.7 F-Q-P-H-P-G-L-Q-K-T-L-E-R-F-H-L-S-S-M-S-S-
L-G-G-P-A-A-F-S-A-R-W-A-Q-E
123 G 3 3828.6 F-Q-P-H-P-G-L-Q-K-T-L-E-N-F-H-L-S-S-M-S-S-
L-G-G-P-A-A-F-S-A-R-W-A-Q-E
124 G 4 3829.6 F-Q-P-H-P-G-L-Q-K-T-L-E-D-F-H-L-S-S-M-S-S-
L-G-G-P-A-A-F-S-A-R-W-A-Q-E
125 G 5 3817.6 F-Q-P-H-P-G-L-Q-K-T-L-E-C-F-H-L-S-S-M-S-S-
L-G-G-P-A-A-F-S-A-R-W-A-Q-E
126 G 6 3842.6 F-Q-P-H-P-G-L-Q-K-T-L-E-Q-F-H-L-S-S-M-S-S-
L-G-G-P-A-A-F-S-A-R-W-A-Q-E
127 G 7 3843.6 F-Q-P-H-P-G-L-Q-K-T-L-E-E-F-H-L-S-S-M-S-S-
L-G-G-P-A-A-F-S-A-R-W-A-Q-E
128 G 8 3771.6 F-Q-P-H-P-G-L-Q-K-T-L-E-G-F-H-L-S-S-M-S-S-
L-G-G-P-A-A-F-S-A-R-W-A-Q-E
129 G 9 3851.6 F-Q-P-H-P-G-L-Q-K-T-L-E-H-F-H-L-S-S-M-S-S-
L-G-G-P-A-A-F-S-A-R-W-A-Q-E
130 G10 3827.7 F-Q-P-H-P-G-L-Q-K-T-L-E-I-F-H-L-S-S-M-S-S-
L-G-G-P-A-A-F-S-A-R-W-A-Q-E
131 G11 3827.7 F-Q-P-H-P-G-L-Q-K-T-L-E-L-F-H-L-S-S-M-S-S-
L-G-G-P-A-A-F-S-A-R-W-A-Q-E
132 G12 3842.7 F-Q-P-H-P-G-L-Q-K-T-L-E-K-F-H-L-S-S-M-S-S-
L-G-G-P-A-A-F-S-A-R-W-A-Q-E
133 G13 3845.7 F-Q-P-H-P-G-L-Q-K-T-L-E-M-F-H-L-S-S-M-S-S-

L-G-G-P-A-A-F-S-A-R-W-A-Q-E
134 G14 3861.7 F-Q-P-H-P-G-L-Q-K-T-L-E-F-F-H-L-S-S-M-S-S-
L-G-G-P-A-A-F-S-A-R-W-A-Q-E
135 G15 3811.6 F-Q-P-H-P-G-L-Q-K-T-L-E-P-F-H-L-S-S-M-S-S-
L-G-G-P-A-A-F-S-A-R-W-A-Q-E
136 G16 3801.6 F-Q-P-H-P-G-L-Q-K-T-L-E-S-F-H-L-S-S-M-S-S-
L-G-G-P-A-A-F-S-A-R-W-A-Q-E
137 G17 3815.6 F-Q-P-H-P-G-L-Q-K-T-L-E-T-F-H-L-S-S-M-S-S-
L-G-G-P-A-A-F-S-A-R-W-A-Q-E
138 G18 3900.7 F-Q-P-H-P-G-L-Q-K-T-L-E-W-F-H-L-S-S-M-S-S-
L-G-G-P-A-A-F-S-A-R-W-A-Q-E
139 G19 3877.7 F-Q-P-H-P-G-L-Q-K-T-L-E-Y-F-H-L-S-S-M-S-S-
L-G-G-P-A-A-F-S-A-R-W-A-Q-E
140 G20 3813.6 F-Q-P-H-P-G-L-Q-K-T-L-E-V-F-H-L-S-S-M-S-S-
L-G-G-P-A-A-F-S-A-R-W-A-Q-E
141 H 1 3766.5 F-Q-P-H-P-G-L-Q-K-T-L-E-Q-A-H-L-S-S-M-S-S-
L-G-G-P-A-A-F-S-A-R-W-A-Q-E
142 H 2 3851.6 F-Q-P-H-P-G-L-Q-K-T-L-E-Q-R-H-L-S-S-M-S-S-
L-G-G-P-A-A-F-S-A-R-W-A-Q-E
143 H 3 3809.5 F-Q-P-H-P-G-L-Q-K-T-L-E-Q-N-H-L-S-S-M-S-S-
L-G-G-P-A-A-F-S-A-R-W-A-Q-E
144 H 4 3810.5 F-Q-P-H-P-G-L-Q-K-T-L-E-Q-D-H-L-S-S-M-S-S-
L-G-G-P-A-A-F-S-A-R-W-A-Q-E
145 H 5 3798.5 F-Q-P-H-P-G-L-Q-K-T-L-E-Q-C-H-L-S-S-M-S-S-
L-G-G-P-A-A-F-S-A-R-W-A-Q-E
146 H 6 3823.5 F-Q-P-H-P-G-L-Q-K-T-L-E-Q-Q-H-L-S-S-M-S-S-
L-G-G-P-A-A-F-S-A-R-W-A-Q-E
147 H 7 3824.5 F-Q-P-H-P-G-L-Q-K-T-L-E-Q-E-H-L-S-S-M-S-S-
L-G-G-P-A-A-F-S-A-R-W-A-Q-E
148 H 8 3752.5 F-Q-P-H-P-G-L-Q-K-T-L-E-Q-G-H-L-S-S-M-S-S-
L-G-G-P-A-A-F-S-A-R-W-A-Q-E
149 H 9 3832.5 F-Q-P-H-P-G-L-Q-K-T-L-E-Q-H-H-L-S-S-M-S-S-
L-G-G-P-A-A-F-S-A-R-W-A-Q-E
150 H10 3808.6 F-Q-P-H-P-G-L-Q-K-T-L-E-Q-I-H-L-S-S-M-S-S-
L-G-G-P-A-A-F-S-A-R-W-A-Q-E
151 H11 3808.6 F-Q-P-H-P-G-L-Q-K-T-L-E-Q-L-H-L-S-S-M-S-S-
L-G-G-P-A-A-F-S-A-R-W-A-Q-E
152 H12 3823.6 F-Q-P-H-P-G-L-Q-K-T-L-E-Q-K-H-L-S-S-M-S-S-
L-G-G-P-A-A-F-S-A-R-W-A-Q-E
153 H13 3826.6 F-Q-P-H-P-G-L-Q-K-T-L-E-Q-M-H-L-S-S-M-S-S-
L-G-G-P-A-A-F-S-A-R-W-A-Q-E
154 H14 3842.6 F-Q-P-H-P-G-L-Q-K-T-L-E-Q-F-H-L-S-S-M-S-S-
L-G-G-P-A-A-F-S-A-R-W-A-Q-E
155 H15 3792.5 F-Q-P-H-P-G-L-Q-K-T-L-E-Q-P-H-L-S-S-M-S-S-
L-G-G-P-A-A-F-S-A-R-W-A-Q-E
156 H16 3782.5 F-Q-P-H-P-G-L-Q-K-T-L-E-Q-S-H-L-S-S-M-S-S-
L-G-G-P-A-A-F-S-A-R-W-A-Q-E
157 H17 3796.5 F-Q-P-H-P-G-L-Q-K-T-L-E-Q-T-H-L-S-S-M-S-S-
L-G-G-P-A-A-F-S-A-R-W-A-Q-E
158 H18 3881.6 F-Q-P-H-P-G-L-Q-K-T-L-E-Q-W-H-L-S-S-M-S-S-
L-G-G-P-A-A-F-S-A-R-W-A-Q-E
159 H19 3858.6 F-Q-P-H-P-G-L-Q-K-T-L-E-Q-Y-H-L-S-S-M-S-S-
L-G-G-P-A-A-F-S-A-R-W-A-Q-E
160 H20 3794.5 F-Q-P-H-P-G-L-Q-K-T-L-E-Q-V-H-L-S-S-M-S-S-

L-G-G-P-A-A-F-S-A-R-W-A-Q-E
161 I 1 3776.6 F-Q-P-H-P-G-L-Q-K-T-L-E-Q-F-A-L-S-S-M-S-S-L-G-G-P-A-A-F-S-A-R-W-A-Q-E
162 I 2 3861.7 F-Q-P-H-P-G-L-Q-K-T-L-E-Q-F-R-L-S-S-M-S-S-L-G-G-P-A-A-F-S-A-R-W-A-Q-E
163 I 3 3819.6 F-Q-P-H-P-G-L-Q-K-T-L-E-Q-F-N-L-S-S-M-S-S-L-G-G-P-A-A-F-S-A-R-W-A-Q-E
164 I 4 3820.6 F-Q-P-H-P-G-L-Q-K-T-L-E-Q-F-D-L-S-S-M-S-S-L-G-G-P-A-A-F-S-A-R-W-A-Q-E
165 I 5 3808.6 F-Q-P-H-P-G-L-Q-K-T-L-E-Q-F-C-L-S-S-M-S-S-L-G-G-P-A-A-F-S-A-R-W-A-Q-E
166 I 6 3833.6 F-Q-P-H-P-G-L-Q-K-T-L-E-Q-F-Q-L-S-S-M-S-S-L-G-G-P-A-A-F-S-A-R-W-A-Q-E
167 I 7 3834.6 F-Q-P-H-P-G-L-Q-K-T-L-E-Q-F-E-L-S-S-M-S-S-L-G-G-P-A-A-F-S-A-R-W-A-Q-E
168 I 8 3762.6 F-Q-P-H-P-G-L-Q-K-T-L-E-Q-F-G-L-S-S-M-S-S-L-G-G-P-A-A-F-S-A-R-W-A-Q-E
169 I 9 3842.6 F-Q-P-H-P-G-L-Q-K-T-L-E-Q-F-H-L-S-S-M-S-S-L-G-G-P-A-A-F-S-A-R-W-A-Q-E
170 I10 3818.7 F-Q-P-H-P-G-L-Q-K-T-L-E-Q-F-I-L-S-S-M-S-S-L-G-G-P-A-A-F-S-A-R-W-A-Q-E
171 I11 3818.7 F-Q-P-H-P-G-L-Q-K-T-L-E-Q-F-L-L-S-S-M-S-S-L-G-G-P-A-A-F-S-A-R-W-A-Q-E
172 I12 3833.7 F-Q-P-H-P-G-L-Q-K-T-L-E-Q-F-K-L-S-S-M-S-S-L-G-G-P-A-A-F-S-A-R-W-A-Q-E
173 I13 3836.7 F-Q-P-H-P-G-L-Q-K-T-L-E-Q-F-M-L-S-S-M-S-S-L-G-G-P-A-A-F-S-A-R-W-A-Q-E
174 I14 3852.7 F-Q-P-H-P-G-L-Q-K-T-L-E-Q-F-F-L-S-S-M-S-S-L-G-G-P-A-A-F-S-A-R-W-A-Q-E
175 I15 3802.6 F-Q-P-H-P-G-L-Q-K-T-L-E-Q-F-P-L-S-S-M-S-S-L-G-G-P-A-A-F-S-A-R-W-A-Q-E
176 I16 3792.6 F-Q-P-H-P-G-L-Q-K-T-L-E-Q-F-S-L-S-S-M-S-S-L-G-G-P-A-A-F-S-A-R-W-A-Q-E
177 I17 3806.6 F-Q-P-H-P-G-L-Q-K-T-L-E-Q-F-T-L-S-S-M-S-S-L-G-G-P-A-A-F-S-A-R-W-A-Q-E
178 I18 3891.7 F-Q-P-H-P-G-L-Q-K-T-L-E-Q-F-W-L-S-S-M-S-S-L-G-G-P-A-A-F-S-A-R-W-A-Q-E
179 I19 3868.7 F-Q-P-H-P-G-L-Q-K-T-L-E-Q-F-Y-L-S-S-M-S-S-L-G-G-P-A-A-F-S-A-R-W-A-Q-E
180 I20 3804.6 F-Q-P-H-P-G-L-Q-K-T-L-E-Q-F-V-L-S-S-M-S-S-L-G-G-P-A-A-F-S-A-R-W-A-Q-E
181 J 1 3800.5 F-Q-P-H-P-G-L-Q-K-T-L-E-Q-F-H-A-S-S-M-S-S-L-G-G-P-A-A-F-S-A-R-W-A-Q-E
182 J 2 3885.6 F-Q-P-H-P-G-L-Q-K-T-L-E-Q-F-H-R-S-S-M-S-S-L-G-G-P-A-A-F-S-A-R-W-A-Q-E
183 J 3 3843.5 F-Q-P-H-P-G-L-Q-K-T-L-E-Q-F-H-N-S-S-M-S-S-L-G-G-P-A-A-F-S-A-R-W-A-Q-E
184 J 4 3844.5 F-Q-P-H-P-G-L-Q-K-T-L-E-Q-F-H-D-S-S-M-S-S-L-G-G-P-A-A-F-S-A-R-W-A-Q-E
185 J 5 3832.5 F-Q-P-H-P-G-L-Q-K-T-L-E-Q-F-H-C-S-S-M-S-S-L-G-G-P-A-A-F-S-A-R-W-A-Q-E
186 J 6 3857.5 F-Q-P-H-P-G-L-Q-K-T-L-E-Q-F-H-Q-S-S-M-S-S-L-G-G-P-A-A-F-S-A-R-W-A-Q-E
187 J 7 3858.5 F-Q-P-H-P-G-L-Q-K-T-L-E-Q-F-H-E-S-S-M-S-S-

L-G-G-P-A-A-F-S-A-R-W-A-Q-E
188 J 8 3786.5 F-Q-P-H-P-G-L-Q-K-T-L-E-Q-F-H-G-S-S-M-S-S-
L-G-G-P-A-A-F-S-A-R-W-A-Q-E
189 J 9 3866.5 F-Q-P-H-P-G-L-Q-K-T-L-E-Q-F-H-H-S-S-M-S-S-
L-G-G-P-A-A-F-S-A-R-W-A-Q-E
190 J10 3842.6 F-Q-P-H-P-G-L-Q-K-T-L-E-Q-F-H-I-S-S-M-S-S-
L-G-G-P-A-A-F-S-A-R-W-A-Q-E
191 J11 3842.6 F-Q-P-H-P-G-L-Q-K-T-L-E-Q-F-H-L-S-S-M-S-S-
L-G-G-P-A-A-F-S-A-R-W-A-Q-E
192 J12 3857.6 F-Q-P-H-P-G-L-Q-K-T-L-E-Q-F-H-K-S-S-M-S-S-
L-G-G-P-A-A-F-S-A-R-W-A-Q-E
193 J13 3860.6 F-Q-P-H-P-G-L-Q-K-T-L-E-Q-F-H-M-S-S-M-S-S-
L-G-G-P-A-A-F-S-A-R-W-A-Q-E
194 J14 3876.6 F-Q-P-H-P-G-L-Q-K-T-L-E-Q-F-H-F-S-S-M-S-S-
L-G-G-P-A-A-F-S-A-R-W-A-Q-E
195 J15 3826.5 F-Q-P-H-P-G-L-Q-K-T-L-E-Q-F-H-P-S-S-M-S-S-
L-G-G-P-A-A-F-S-A-R-W-A-Q-E
196 J16 3816.5 F-Q-P-H-P-G-L-Q-K-T-L-E-Q-F-H-S-S-S-M-S-S-
L-G-G-P-A-A-F-S-A-R-W-A-Q-E
197 J17 3830.5 F-Q-P-H-P-G-L-Q-K-T-L-E-Q-F-H-T-S-S-M-S-S-
L-G-G-P-A-A-F-S-A-R-W-A-Q-E
198 J18 3915.6 F-Q-P-H-P-G-L-Q-K-T-L-E-Q-F-H-W-S-S-M-S-S-
L-G-G-P-A-A-F-S-A-R-W-A-Q-E
199 J19 3892.6 F-Q-P-H-P-G-L-Q-K-T-L-E-Q-F-H-Y-S-S-M-S-S-
L-G-G-P-A-A-F-S-A-R-W-A-Q-E
200 J20 3828.5 F-Q-P-H-P-G-L-Q-K-T-L-E-Q-F-H-V-S-S-M-S-S-
L-G-G-P-A-A-F-S-A-R-W-A-Q-E
201 K 1 3826.6 F-Q-P-H-P-G-L-Q-K-T-L-E-Q-F-H-L-A-S-M-S-S-
L-G-G-P-A-A-F-S-A-R-W-A-Q-E
202 K 2 3911.7 F-Q-P-H-P-G-L-Q-K-T-L-E-Q-F-H-L-R-S-M-S-S-
L-G-G-P-A-A-F-S-A-R-W-A-Q-E
203 K 3 3869.6 F-Q-P-H-P-G-L-Q-K-T-L-E-Q-F-H-L-N-S-M-S-S-
L-G-G-P-A-A-F-S-A-R-W-A-Q-E
204 K 4 3870.6 F-Q-P-H-P-G-L-Q-K-T-L-E-Q-F-H-L-D-S-M-S-S-
L-G-G-P-A-A-F-S-A-R-W-A-Q-E
205 K 5 3858.6 F-Q-P-H-P-G-L-Q-K-T-L-E-Q-F-H-L-C-S-M-S-S-
L-G-G-P-A-A-F-S-A-R-W-A-Q-E
206 K 6 3883.6 F-Q-P-H-P-G-L-Q-K-T-L-E-Q-F-H-L-Q-S-M-S-S-
L-G-G-P-A-A-F-S-A-R-W-A-Q-E
207 K 7 3884.6 F-Q-P-H-P-G-L-Q-K-T-L-E-Q-F-H-L-E-S-M-S-S-
L-G-G-P-A-A-F-S-A-R-W-A-Q-E
208 K 8 3812.6 F-Q-P-H-P-G-L-Q-K-T-L-E-Q-F-H-L-G-S-M-S-S-
L-G-G-P-A-A-F-S-A-R-W-A-Q-E
209 K 9 3892.6 F-Q-P-H-P-G-L-Q-K-T-L-E-Q-F-H-L-H-S-M-S-S-
L-G-G-P-A-A-F-S-A-R-W-A-Q-E
210 K10 3868.7 F-Q-P-H-P-G-L-Q-K-T-L-E-Q-F-H-L-I-S-M-S-S-
L-G-G-P-A-A-F-S-A-R-W-A-Q-E
211 K11 3868.7 F-Q-P-H-P-G-L-Q-K-T-L-E-Q-F-H-L-L-S-M-S-S-
L-G-G-P-A-A-F-S-A-R-W-A-Q-E
212 K12 3883.7 F-Q-P-H-P-G-L-Q-K-T-L-E-Q-F-H-L-K-S-M-S-S-
L-G-G-P-A-A-F-S-A-R-W-A-Q-E
213 K13 3886.7 F-Q-P-H-P-G-L-Q-K-T-L-E-Q-F-H-L-M-S-M-S-S-
L-G-G-P-A-A-F-S-A-R-W-A-Q-E
214 K14 3902.7 F-Q-P-H-P-G-L-Q-K-T-L-E-Q-F-H-L-F-S-M-S-S-

L-G-G-P-A-A-F-S-A-R-W-A-Q-E
215 K15 3852.6 F-Q-P-H-P-G-L-Q-K-T-L-E-Q-F-H-L-P-S-M-S-S-
L-G-G-P-A-A-F-S-A-R-W-A-Q-E
216 K16 3842.6 F-Q-P-H-P-G-L-Q-K-T-L-E-Q-F-H-L-S-S-M-S-S-
L-G-G-P-A-A-F-S-A-R-W-A-Q-E
217 K17 3856.6 F-Q-P-H-P-G-L-Q-K-T-L-E-Q-F-H-L-T-S-M-S-S-
L-G-G-P-A-A-F-S-A-R-W-A-Q-E
218 K18 3941.7 F-Q-P-H-P-G-L-Q-K-T-L-E-Q-F-H-L-W-S-M-S-S-
L-G-G-P-A-A-F-S-A-R-W-A-Q-E
219 K19 3918.7 F-Q-P-H-P-G-L-Q-K-T-L-E-Q-F-H-L-Y-S-M-S-S-
L-G-G-P-A-A-F-S-A-R-W-A-Q-E
220 K20 3854.6 F-Q-P-H-P-G-L-Q-K-T-L-E-Q-F-H-L-V-S-M-S-S-
L-G-G-P-A-A-F-S-A-R-W-A-Q-E
221 L 1 3826.6 F-Q-P-H-P-G-L-Q-K-T-L-E-Q-F-H-L-S-A-M-S-S-
L-G-G-P-A-A-F-S-A-R-W-A-Q-E
222 L 2 3911.7 F-Q-P-H-P-G-L-Q-K-T-L-E-Q-F-H-L-S-R-M-S-S-
L-G-G-P-A-A-F-S-A-R-W-A-Q-E
223 L 3 3869.6 F-Q-P-H-P-G-L-Q-K-T-L-E-Q-F-H-L-S-N-M-S-S-
L-G-G-P-A-A-F-S-A-R-W-A-Q-E
224 L 4 3870.6 F-Q-P-H-P-G-L-Q-K-T-L-E-Q-F-H-L-S-D-M-S-S-
L-G-G-P-A-A-F-S-A-R-W-A-Q-E
225 L 5 3858.6 F-Q-P-H-P-G-L-Q-K-T-L-E-Q-F-H-L-S-C-M-S-S-
L-G-G-P-A-A-F-S-A-R-W-A-Q-E
226 L 6 3883.6 F-Q-P-H-P-G-L-Q-K-T-L-E-Q-F-H-L-S-Q-M-S-S-
L-G-G-P-A-A-F-S-A-R-W-A-Q-E
227 L 7 3884.6 F-Q-P-H-P-G-L-Q-K-T-L-E-Q-F-H-L-S-E-M-S-S-
L-G-G-P-A-A-F-S-A-R-W-A-Q-E
228 L 8 3812.6 F-Q-P-H-P-G-L-Q-K-T-L-E-Q-F-H-L-S-G-M-S-S-
L-G-G-P-A-A-F-S-A-R-W-A-Q-E
229 L 9 3892.6 F-Q-P-H-P-G-L-Q-K-T-L-E-Q-F-H-L-S-H-M-S-S-
L-G-G-P-A-A-F-S-A-R-W-A-Q-E
230 L10 3868.7 F-Q-P-H-P-G-L-Q-K-T-L-E-Q-F-H-L-S-I-M-S-S-
L-G-G-P-A-A-F-S-A-R-W-A-Q-E
231 L11 3868.7 F-Q-P-H-P-G-L-Q-K-T-L-E-Q-F-H-L-S-L-M-S-S-
L-G-G-P-A-A-F-S-A-R-W-A-Q-E
232 L12 3883.7 F-Q-P-H-P-G-L-Q-K-T-L-E-Q-F-H-L-S-K-M-S-S-
L-G-G-P-A-A-F-S-A-R-W-A-Q-E
233 L13 3886.7 F-Q-P-H-P-G-L-Q-K-T-L-E-Q-F-H-L-S-M-M-S-S-
L-G-G-P-A-A-F-S-A-R-W-A-Q-E
234 L14 3902.7 F-Q-P-H-P-G-L-Q-K-T-L-E-Q-F-H-L-S-F-M-S-S-
L-G-G-P-A-A-F-S-A-R-W-A-Q-E
235 L15 3852.6 F-Q-P-H-P-G-L-Q-K-T-L-E-Q-F-H-L-S-P-M-S-S-
L-G-G-P-A-A-F-S-A-R-W-A-Q-E
236 L16 3842.6 F-Q-P-H-P-G-L-Q-K-T-L-E-Q-F-H-L-S-S-M-S-S-
L-G-G-P-A-A-F-S-A-R-W-A-Q-E
237 L17 3856.6 F-Q-P-H-P-G-L-Q-K-T-L-E-Q-F-H-L-S-T-M-S-S-
L-G-G-P-A-A-F-S-A-R-W-A-Q-E
238 L18 3941.7 F-Q-P-H-P-G-L-Q-K-T-L-E-Q-F-H-L-S-W-M-S-S-
L-G-G-P-A-A-F-S-A-R-W-A-Q-E
239 L19 3918.7 F-Q-P-H-P-G-L-Q-K-T-L-E-Q-F-H-L-S-Y-M-S-S-
L-G-G-P-A-A-F-S-A-R-W-A-Q-E
240 L20 3854.6 F-Q-P-H-P-G-L-Q-K-T-L-E-Q-F-H-L-S-V-M-S-S-
L-G-G-P-A-A-F-S-A-R-W-A-Q-E
241 M 1 3782.5 F-Q-P-H-P-G-L-Q-K-T-L-E-Q-F-H-L-S-S-A-S-S-

L-G-G-P-A-A-F-S-A-R-W-A-Q-E
242 M 2 3867.6 F-Q-P-H-P-G-L-Q-K-T-L-E-Q-F-H-L-S-S-R-S-S-
L-G-G-P-A-A-F-S-A-R-W-A-Q-E
243 M 3 3825.5 F-Q-P-H-P-G-L-Q-K-T-L-E-Q-F-H-L-S-S-N-S-S-
L-G-G-P-A-A-F-S-A-R-W-A-Q-E
244 M 4 3826.5 F-Q-P-H-P-G-L-Q-K-T-L-E-Q-F-H-L-S-S-D-S-S-
L-G-G-P-A-A-F-S-A-R-W-A-Q-E
245 M 5 3814.5 F-Q-P-H-P-G-L-Q-K-T-L-E-Q-F-H-L-S-S-C-S-S-
L-G-G-P-A-A-F-S-A-R-W-A-Q-E
246 M 6 3839.5 F-Q-P-H-P-G-L-Q-K-T-L-E-Q-F-H-L-S-S-Q-S-S-
L-G-G-P-A-A-F-S-A-R-W-A-Q-E
247 M 7 3840.5 F-Q-P-H-P-G-L-Q-K-T-L-E-Q-F-H-L-S-S-E-S-S-
L-G-G-P-A-A-F-S-A-R-W-A-Q-E
248 M 8 3768.5 F-Q-P-H-P-G-L-Q-K-T-L-E-Q-F-H-L-S-S-G-S-S-
L-G-G-P-A-A-F-S-A-R-W-A-Q-E
249 M 9 3848.5 F-Q-P-H-P-G-L-Q-K-T-L-E-Q-F-H-L-S-S-H-S-S-
L-G-G-P-A-A-F-S-A-R-W-A-Q-E
250 M10 3824.6 F-Q-P-H-P-G-L-Q-K-T-L-E-Q-F-H-L-S-S-I-S-S-
L-G-G-P-A-A-F-S-A-R-W-A-Q-E
251 M11 3824.6 F-Q-P-H-P-G-L-Q-K-T-L-E-Q-F-H-L-S-S-L-S-S-
L-G-G-P-A-A-F-S-A-R-W-A-Q-E
252 M12 3839.6 F-Q-P-H-P-G-L-Q-K-T-L-E-Q-F-H-L-S-S-K-S-S-
L-G-G-P-A-A-F-S-A-R-W-A-Q-E
253 M13 3842.6 F-Q-P-H-P-G-L-Q-K-T-L-E-Q-F-H-L-S-S-M-S-S-
L-G-G-P-A-A-F-S-A-R-W-A-Q-E
254 M14 3858.6 F-Q-P-H-P-G-L-Q-K-T-L-E-Q-F-H-L-S-S-F-S-S-
L-G-G-P-A-A-F-S-A-R-W-A-Q-E
255 M15 3808.5 F-Q-P-H-P-G-L-Q-K-T-L-E-Q-F-H-L-S-S-P-S-S-
L-G-G-P-A-A-F-S-A-R-W-A-Q-E
256 M16 3798.5 F-Q-P-H-P-G-L-Q-K-T-L-E-Q-F-H-L-S-S-S-S-S-
L-G-G-P-A-A-F-S-A-R-W-A-Q-E
257 M17 3812.5 F-Q-P-H-P-G-L-Q-K-T-L-E-Q-F-H-L-S-S-T-S-S-
L-G-G-P-A-A-F-S-A-R-W-A-Q-E
258 M18 3897.6 F-Q-P-H-P-G-L-Q-K-T-L-E-Q-F-H-L-S-S-W-S-S-
L-G-G-P-A-A-F-S-A-R-W-A-Q-E
259 M19 3874.6 F-Q-P-H-P-G-L-Q-K-T-L-E-Q-F-H-L-S-S-Y-S-S-
L-G-G-P-A-A-F-S-A-R-W-A-Q-E
260 M20 3810.5 F-Q-P-H-P-G-L-Q-K-T-L-E-Q-F-H-L-S-S-V-S-S-
L-G-G-P-A-A-F-S-A-R-W-A-Q-E
261 N 1 3826.6 F-Q-P-H-P-G-L-Q-K-T-L-E-Q-F-H-L-S-S-M-A-S-
L-G-G-P-A-A-F-S-A-R-W-A-Q-E
262 N 2 3911.7 F-Q-P-H-P-G-L-Q-K-T-L-E-Q-F-H-L-S-S-M-R-S-
L-G-G-P-A-A-F-S-A-R-W-A-Q-E
263 N 3 3869.6 F-Q-P-H-P-G-L-Q-K-T-L-E-Q-F-H-L-S-S-M-N-S-
L-G-G-P-A-A-F-S-A-R-W-A-Q-E
264 N 4 3870.6 F-Q-P-H-P-G-L-Q-K-T-L-E-Q-F-H-L-S-S-M-D-S-
L-G-G-P-A-A-F-S-A-R-W-A-Q-E
265 N 5 3858.6 F-Q-P-H-P-G-L-Q-K-T-L-E-Q-F-H-L-S-S-M-C-S-
L-G-G-P-A-A-F-S-A-R-W-A-Q-E
266 N 6 3883.6 F-Q-P-H-P-G-L-Q-K-T-L-E-Q-F-H-L-S-S-M-Q-S-
L-G-G-P-A-A-F-S-A-R-W-A-Q-E
267 N 7 3884.6 F-Q-P-H-P-G-L-Q-K-T-L-E-Q-F-H-L-S-S-M-E-S-
L-G-G-P-A-A-F-S-A-R-W-A-Q-E
268 N 8 3812.6 F-Q-P-H-P-G-L-Q-K-T-L-E-Q-F-H-L-S-S-M-G-S-

L-G-G-P-A-A-F-S-A-R-W-A-Q-E
269 N 9 3892.6 F-Q-P-H-P-G-L-Q-K-T-L-E-Q-F-H-L-S-S-M-H-S-
L-G-G-P-A-A-F-S-A-R-W-A-Q-E
270 N10 3868.7 F-Q-P-H-P-G-L-Q-K-T-L-E-Q-F-H-L-S-S-M-I-S-
L-G-G-P-A-A-F-S-A-R-W-A-Q-E
271 N11 3868.7 F-Q-P-H-P-G-L-Q-K-T-L-E-Q-F-H-L-S-S-M-L-S-
L-G-G-P-A-A-F-S-A-R-W-A-Q-E
272 N12 3883.7 F-Q-P-H-P-G-L-Q-K-T-L-E-Q-F-H-L-S-S-M-K-S-
L-G-G-P-A-A-F-S-A-R-W-A-Q-E
273 N13 3886.7 F-Q-P-H-P-G-L-Q-K-T-L-E-Q-F-H-L-S-S-M-M-S-
L-G-G-P-A-A-F-S-A-R-W-A-Q-E
274 N14 3902.7 F-Q-P-H-P-G-L-Q-K-T-L-E-Q-F-H-L-S-S-M-F-S-
L-G-G-P-A-A-F-S-A-R-W-A-Q-E
275 N15 3852.6 F-Q-P-H-P-G-L-Q-K-T-L-E-Q-F-H-L-S-S-M-P-S-
L-G-G-P-A-A-F-S-A-R-W-A-Q-E
276 N16 3842.6 F-Q-P-H-P-G-L-Q-K-T-L-E-Q-F-H-L-S-S-M-S-S-
L-G-G-P-A-A-F-S-A-R-W-A-Q-E
277 N17 3856.6 F-Q-P-H-P-G-L-Q-K-T-L-E-Q-F-H-L-S-S-M-T-S-
L-G-G-P-A-A-F-S-A-R-W-A-Q-E
278 N18 3941.7 F-Q-P-H-P-G-L-Q-K-T-L-E-Q-F-H-L-S-S-M-W-S-
L-G-G-P-A-A-F-S-A-R-W-A-Q-E
279 N19 3918.7 F-Q-P-H-P-G-L-Q-K-T-L-E-Q-F-H-L-S-S-M-Y-S-
L-G-G-P-A-A-F-S-A-R-W-A-Q-E
280 N20 3854.6 F-Q-P-H-P-G-L-Q-K-T-L-E-Q-F-H-L-S-S-M-V-S-
L-G-G-P-A-A-F-S-A-R-W-A-Q-E
281 O 1 3826.6 F-Q-P-H-P-G-L-Q-K-T-L-E-Q-F-H-L-S-S-M-S-A-
L-G-G-P-A-A-F-S-A-R-W-A-Q-E
282 O 2 3911.7 F-Q-P-H-P-G-L-Q-K-T-L-E-Q-F-H-L-S-S-M-S-R-
L-G-G-P-A-A-F-S-A-R-W-A-Q-E
283 O 3 3869.6 F-Q-P-H-P-G-L-Q-K-T-L-E-Q-F-H-L-S-S-M-S-N-
L-G-G-P-A-A-F-S-A-R-W-A-Q-E
284 O 4 3870.6 F-Q-P-H-P-G-L-Q-K-T-L-E-Q-F-H-L-S-S-M-S-D-
L-G-G-P-A-A-F-S-A-R-W-A-Q-E
285 O 5 3858.6 F-Q-P-H-P-G-L-Q-K-T-L-E-Q-F-H-L-S-S-M-S-C-
L-G-G-P-A-A-F-S-A-R-W-A-Q-E
286 O 6 3883.6 F-Q-P-H-P-G-L-Q-K-T-L-E-Q-F-H-L-S-S-M-S-Q-
L-G-G-P-A-A-F-S-A-R-W-A-Q-E
287 O 7 3884.6 F-Q-P-H-P-G-L-Q-K-T-L-E-Q-F-H-L-S-S-M-S-E-
L-G-G-P-A-A-F-S-A-R-W-A-Q-E
288 O 8 3812.6 F-Q-P-H-P-G-L-Q-K-T-L-E-Q-F-H-L-S-S-M-S-G-
L-G-G-P-A-A-F-S-A-R-W-A-Q-E
289 O 9 3892.6 F-Q-P-H-P-G-L-Q-K-T-L-E-Q-F-H-L-S-S-M-S-H-
L-G-G-P-A-A-F-S-A-R-W-A-Q-E
290 O10 3868.7 F-Q-P-H-P-G-L-Q-K-T-L-E-Q-F-H-L-S-S-M-S-I-
L-G-G-P-A-A-F-S-A-R-W-A-Q-E
291 O11 3868.7 F-Q-P-H-P-G-L-Q-K-T-L-E-Q-F-H-L-S-S-M-S-L-
L-G-G-P-A-A-F-S-A-R-W-A-Q-E
292 O12 3883.7 F-Q-P-H-P-G-L-Q-K-T-L-E-Q-F-H-L-S-S-M-S-K-
L-G-G-P-A-A-F-S-A-R-W-A-Q-E
293 O13 3886.7 F-Q-P-H-P-G-L-Q-K-T-L-E-Q-F-H-L-S-S-M-S-M-
L-G-G-P-A-A-F-S-A-R-W-A-Q-E
294 O14 3902.7 F-Q-P-H-P-G-L-Q-K-T-L-E-Q-F-H-L-S-S-M-S-F-
L-G-G-P-A-A-F-S-A-R-W-A-Q-E
295 O15 3852.6 F-Q-P-H-P-G-L-Q-K-T-L-E-Q-F-H-L-S-S-M-S-P-

L-G-G-P-A-A-F-S-A-R-W-A-Q-E
296 O16 3842.6 F-Q-P-H-P-G-L-Q-K-T-L-E-Q-F-H-L-S-S-M-S-S-
L-G-G-P-A-A-F-S-A-R-W-A-Q-E
297 O17 3856.6 F-Q-P-H-P-G-L-Q-K-T-L-E-Q-F-H-L-S-S-M-S-T-
L-G-G-P-A-A-F-S-A-R-W-A-Q-E
298 O18 3941.7 F-Q-P-H-P-G-L-Q-K-T-L-E-Q-F-H-L-S-S-M-S-W-
L-G-G-P-A-A-F-S-A-R-W-A-Q-E
299 O19 3918.7 F-Q-P-H-P-G-L-Q-K-T-L-E-Q-F-H-L-S-S-M-S-Y-
L-G-G-P-A-A-F-S-A-R-W-A-Q-E
300 O20 3854.6 F-Q-P-H-P-G-L-Q-K-T-L-E-Q-F-H-L-S-S-M-S-V-
L-G-G-P-A-A-F-S-A-R-W-A-Q-E
301 P 1 3800.5 F-Q-P-H-P-G-L-Q-K-T-L-E-Q-F-H-L-S-S-M-S-S-
A-G-G-P-A-A-F-S-A-R-W-A-Q-E
302 P 2 3885.6 F-Q-P-H-P-G-L-Q-K-T-L-E-Q-F-H-L-S-S-M-S-S-
R-G-G-P-A-A-F-S-A-R-W-A-Q-E
303 P 3 3843.5 F-Q-P-H-P-G-L-Q-K-T-L-E-Q-F-H-L-S-S-M-S-S-
N-G-G-P-A-A-F-S-A-R-W-A-Q-E
304 P 4 3844.5 F-Q-P-H-P-G-L-Q-K-T-L-E-Q-F-H-L-S-S-M-S-S-
D-G-G-P-A-A-F-S-A-R-W-A-Q-E
305 P 5 3832.5 F-Q-P-H-P-G-L-Q-K-T-L-E-Q-F-H-L-S-S-M-S-S-
C-G-G-P-A-A-F-S-A-R-W-A-Q-E
306 P 6 3857.5 F-Q-P-H-P-G-L-Q-K-T-L-E-Q-F-H-L-S-S-M-S-S-
Q-G-G-P-A-A-F-S-A-R-W-A-Q-E
307 P 7 3858.5 F-Q-P-H-P-G-L-Q-K-T-L-E-Q-F-H-L-S-S-M-S-S-
E-G-G-P-A-A-F-S-A-R-W-A-Q-E
308 P 8 3786.5 F-Q-P-H-P-G-L-Q-K-T-L-E-Q-F-H-L-S-S-M-S-S-
G-G-G-P-A-A-F-S-A-R-W-A-Q-E
309 P 9 3866.5 F-Q-P-H-P-G-L-Q-K-T-L-E-Q-F-H-L-S-S-M-S-S-
H-G-G-P-A-A-F-S-A-R-W-A-Q-E
310 P10 3842.6 F-Q-P-H-P-G-L-Q-K-T-L-E-Q-F-H-L-S-S-M-S-S-
I-G-G-P-A-A-F-S-A-R-W-A-Q-E
311 P11 3842.6 F-Q-P-H-P-G-L-Q-K-T-L-E-Q-F-H-L-S-S-M-S-S-
L-G-G-P-A-A-F-S-A-R-W-A-Q-E
312 P12 3857.6 F-Q-P-H-P-G-L-Q-K-T-L-E-Q-F-H-L-S-S-M-S-S-
K-G-G-P-A-A-F-S-A-R-W-A-Q-E
313 P13 3860.6 F-Q-P-H-P-G-L-Q-K-T-L-E-Q-F-H-L-S-S-M-S-S-
M-G-G-P-A-A-F-S-A-R-W-A-Q-E
314 P14 3876.6 F-Q-P-H-P-G-L-Q-K-T-L-E-Q-F-H-L-S-S-M-S-S-
F-G-G-P-A-A-F-S-A-R-W-A-Q-E
315 P15 3826.5 F-Q-P-H-P-G-L-Q-K-T-L-E-Q-F-H-L-S-S-M-S-S-
P-G-G-P-A-A-F-S-A-R-W-A-Q-E
316 P16 3816.5 F-Q-P-H-P-G-L-Q-K-T-L-E-Q-F-H-L-S-S-M-S-S-
S-G-G-P-A-A-F-S-A-R-W-A-Q-E
317 P17 3830.5 F-Q-P-H-P-G-L-Q-K-T-L-E-Q-F-H-L-S-S-M-S-S-
T-G-G-P-A-A-F-S-A-R-W-A-Q-E
318 P18 3915.6 F-Q-P-H-P-G-L-Q-K-T-L-E-Q-F-H-L-S-S-M-S-S-
W-G-G-P-A-A-F-S-A-R-W-A-Q-E
319 P19 3892.6 F-Q-P-H-P-G-L-Q-K-T-L-E-Q-F-H-L-S-S-M-S-S-
Y-G-G-P-A-A-F-S-A-R-W-A-Q-E
320 P20 3828.5 F-Q-P-H-P-G-L-Q-K-T-L-E-Q-F-H-L-S-S-M-S-S-
V-G-G-P-A-A-F-S-A-R-W-A-Q-E
321 Q 1 3856.6 F-Q-P-H-P-G-L-Q-K-T-L-E-Q-F-H-L-S-S-M-S-S-
L-A-G-P-A-A-F-S-A-R-W-A-Q-E
322 Q 2 3941.7 F-Q-P-H-P-G-L-Q-K-T-L-E-Q-F-H-L-S-S-M-S-S-

L-R-G-P-A-A-F-S-A-R-W-A-Q-E
323 Q 3 3899.6 F-Q-P-H-P-G-L-Q-K-T-L-E-Q-F-H-L-S-S-M-S-S-
L-N-G-P-A-A-F-S-A-R-W-A-Q-E
324 Q 4 3900.6 F-Q-P-H-P-G-L-Q-K-T-L-E-Q-F-H-L-S-S-M-S-S-
L-D-G-P-A-A-F-S-A-R-W-A-Q-E
325 Q 5 3888.6 F-Q-P-H-P-G-L-Q-K-T-L-E-Q-F-H-L-S-S-M-S-S-
L-C-G-P-A-A-F-S-A-R-W-A-Q-E
326 Q 6 3913.6 F-Q-P-H-P-G-L-Q-K-T-L-E-Q-F-H-L-S-S-M-S-S-
L-Q-G-P-A-A-F-S-A-R-W-A-Q-E
327 Q 7 3914.6 F-Q-P-H-P-G-L-Q-K-T-L-E-Q-F-H-L-S-S-M-S-S-
L-E-G-P-A-A-F-S-A-R-W-A-Q-E
328 Q 8 3842.6 F-Q-P-H-P-G-L-Q-K-T-L-E-Q-F-H-L-S-S-M-S-S-
L-G-G-P-A-A-F-S-A-R-W-A-Q-E
329 Q 9 3922.6 F-Q-P-H-P-G-L-Q-K-T-L-E-Q-F-H-L-S-S-M-S-S-
L-H-G-P-A-A-F-S-A-R-W-A-Q-E
330 Q10 3898.7 F-Q-P-H-P-G-L-Q-K-T-L-E-Q-F-H-L-S-S-M-S-S-
L-I-G-P-A-A-F-S-A-R-W-A-Q-E
331 Q11 3898.7 F-Q-P-H-P-G-L-Q-K-T-L-E-Q-F-H-L-S-S-M-S-S-
L-L-G-P-A-A-F-S-A-R-W-A-Q-E
332 Q12 3913.7 F-Q-P-H-P-G-L-Q-K-T-L-E-Q-F-H-L-S-S-M-S-S-
L-K-G-P-A-A-F-S-A-R-W-A-Q-E
333 Q13 3916.7 F-Q-P-H-P-G-L-Q-K-T-L-E-Q-F-H-L-S-S-M-S-S-
L-M-G-P-A-A-F-S-A-R-W-A-Q-E
334 Q14 3932.7 F-Q-P-H-P-G-L-Q-K-T-L-E-Q-F-H-L-S-S-M-S-S-
L-F-G-P-A-A-F-S-A-R-W-A-Q-E
335 Q15 3882.6 F-Q-P-H-P-G-L-Q-K-T-L-E-Q-F-H-L-S-S-M-S-S-
L-P-G-P-A-A-F-S-A-R-W-A-Q-E
336 Q16 3872.6 F-Q-P-H-P-G-L-Q-K-T-L-E-Q-F-H-L-S-S-M-S-S-
L-S-G-P-A-A-F-S-A-R-W-A-Q-E
337 Q17 3886.6 F-Q-P-H-P-G-L-Q-K-T-L-E-Q-F-H-L-S-S-M-S-S-
L-T-G-P-A-A-F-S-A-R-W-A-Q-E
338 Q18 3971.7 F-Q-P-H-P-G-L-Q-K-T-L-E-Q-F-H-L-S-S-M-S-S-
L-W-G-P-A-A-F-S-A-R-W-A-Q-E
339 Q19 3948.7 F-Q-P-H-P-G-L-Q-K-T-L-E-Q-F-H-L-S-S-M-S-S-
L-Y-G-P-A-A-F-S-A-R-W-A-Q-E
340 Q20 3884.6 F-Q-P-H-P-G-L-Q-K-T-L-E-Q-F-H-L-S-S-M-S-S-
L-V-G-P-A-A-F-S-A-R-W-A-Q-E
341 R 1 3856.6 F-Q-P-H-P-G-L-Q-K-T-L-E-Q-F-H-L-S-S-M-S-S-
L-G-A-P-A-A-F-S-A-R-W-A-Q-E
342 R 2 3941.7 F-Q-P-H-P-G-L-Q-K-T-L-E-Q-F-H-L-S-S-M-S-S-
L-G-R-P-A-A-F-S-A-R-W-A-Q-E
343 R 3 3899.6 F-Q-P-H-P-G-L-Q-K-T-L-E-Q-F-H-L-S-S-M-S-S-
L-G-N-P-A-A-F-S-A-R-W-A-Q-E
344 R 4 3900.6 F-Q-P-H-P-G-L-Q-K-T-L-E-Q-F-H-L-S-S-M-S-S-
L-G-D-P-A-A-F-S-A-R-W-A-Q-E
345 R 5 3888.6 F-Q-P-H-P-G-L-Q-K-T-L-E-Q-F-H-L-S-S-M-S-S-
L-G-C-P-A-A-F-S-A-R-W-A-Q-E
346 R 6 3913.6 F-Q-P-H-P-G-L-Q-K-T-L-E-Q-F-H-L-S-S-M-S-S-
L-G-Q-P-A-A-F-S-A-R-W-A-Q-E
347 R 7 3914.6 F-Q-P-H-P-G-L-Q-K-T-L-E-Q-F-H-L-S-S-M-S-S-
L-G-E-P-A-A-F-S-A-R-W-A-Q-E
348 R 8 3842.6 F-Q-P-H-P-G-L-Q-K-T-L-E-Q-F-H-L-S-S-M-S-S-
L-G-G-P-A-A-F-S-A-R-W-A-Q-E
349 R 9 3922.6 F-Q-P-H-P-G-L-Q-K-T-L-E-Q-F-H-L-S-S-M-S-S-

L-G-H-P-A-A-F-S-A-R-W-A-Q-E
350 R10 3898.7 F-Q-P-H-P-G-L-Q-K-T-L-E-Q-F-H-L-S-S-M-S-S-
L-G-I-P-A-A-F-S-A-R-W-A-Q-E
351 R11 3898.7 F-Q-P-H-P-G-L-Q-K-T-L-E-Q-F-H-L-S-S-M-S-S-
L-G-L-P-A-A-F-S-A-R-W-A-Q-E
352 R12 3913.7 F-Q-P-H-P-G-L-Q-K-T-L-E-Q-F-H-L-S-S-M-S-S-
L-G-K-P-A-A-F-S-A-R-W-A-Q-E
353 R13 3916.7 F-Q-P-H-P-G-L-Q-K-T-L-E-Q-F-H-L-S-S-M-S-S-
L-G-M-P-A-A-F-S-A-R-W-A-Q-E
354 R14 3932.7 F-Q-P-H-P-G-L-Q-K-T-L-E-Q-F-H-L-S-S-M-S-S-
L-G-F-P-A-A-F-S-A-R-W-A-Q-E
355 R15 3882.6 F-Q-P-H-P-G-L-Q-K-T-L-E-Q-F-H-L-S-S-M-S-S-
L-G-P-P-A-A-F-S-A-R-W-A-Q-E
356 R16 3872.6 F-Q-P-H-P-G-L-Q-K-T-L-E-Q-F-H-L-S-S-M-S-S-
L-G-S-P-A-A-F-S-A-R-W-A-Q-E
357 R17 3886.6 F-Q-P-H-P-G-L-Q-K-T-L-E-Q-F-H-L-S-S-M-S-S-
L-G-T-P-A-A-F-S-A-R-W-A-Q-E
358 R18 3971.7 F-Q-P-H-P-G-L-Q-K-T-L-E-Q-F-H-L-S-S-M-S-S-
L-G-W-P-A-A-F-S-A-R-W-A-Q-E
359 R19 3948.7 F-Q-P-H-P-G-L-Q-K-T-L-E-Q-F-H-L-S-S-M-S-S-
L-G-Y-P-A-A-F-S-A-R-W-A-Q-E
360 R20 3884.6 F-Q-P-H-P-G-L-Q-K-T-L-E-Q-F-H-L-S-S-M-S-S-
L-G-V-P-A-A-F-S-A-R-W-A-Q-E
361 S 1 3816.6 F-Q-P-H-P-G-L-Q-K-T-L-E-Q-F-H-L-S-S-M-S-S-
L-G-G-A-A-A-F-S-A-R-W-A-Q-E
362 S 2 3901.7 F-Q-P-H-P-G-L-Q-K-T-L-E-Q-F-H-L-S-S-M-S-S-
L-G-G-R-A-A-F-S-A-R-W-A-Q-E
363 S 3 3859.6 F-Q-P-H-P-G-L-Q-K-T-L-E-Q-F-H-L-S-S-M-S-S-
L-G-G-N-A-A-F-S-A-R-W-A-Q-E
364 S 4 3860.6 F-Q-P-H-P-G-L-Q-K-T-L-E-Q-F-H-L-S-S-M-S-S-
L-G-G-D-A-A-F-S-A-R-W-A-Q-E
365 S 5 3848.6 F-Q-P-H-P-G-L-Q-K-T-L-E-Q-F-H-L-S-S-M-S-S-
L-G-G-C-A-A-F-S-A-R-W-A-Q-E
366 S 6 3873.6 F-Q-P-H-P-G-L-Q-K-T-L-E-Q-F-H-L-S-S-M-S-S-
L-G-G-Q-A-A-F-S-A-R-W-A-Q-E
367 S 7 3874.6 F-Q-P-H-P-G-L-Q-K-T-L-E-Q-F-H-L-S-S-M-S-S-
L-G-G-E-A-A-F-S-A-R-W-A-Q-E
368 S 8 3802.6 F-Q-P-H-P-G-L-Q-K-T-L-E-Q-F-H-L-S-S-M-S-S-
L-G-G-G-A-A-F-S-A-R-W-A-Q-E
369 S 9 3882.6 F-Q-P-H-P-G-L-Q-K-T-L-E-Q-F-H-L-S-S-M-S-S-
L-G-G-H-A-A-F-S-A-R-W-A-Q-E
370 S10 3858.7 F-Q-P-H-P-G-L-Q-K-T-L-E-Q-F-H-L-S-S-M-S-S-
L-G-G-I-A-A-F-S-A-R-W-A-Q-E
371 S11 3858.7 F-Q-P-H-P-G-L-Q-K-T-L-E-Q-F-H-L-S-S-M-S-S-
L-G-G-L-A-A-F-S-A-R-W-A-Q-E
372 S12 3873.7 F-Q-P-H-P-G-L-Q-K-T-L-E-Q-F-H-L-S-S-M-S-S-
L-G-G-K-A-A-F-S-A-R-W-A-Q-E
373 S13 3876.7 F-Q-P-H-P-G-L-Q-K-T-L-E-Q-F-H-L-S-S-M-S-S-
L-G-G-M-A-A-F-S-A-R-W-A-Q-E
374 S14 3892.7 F-Q-P-H-P-G-L-Q-K-T-L-E-Q-F-H-L-S-S-M-S-S-
L-G-G-F-A-A-F-S-A-R-W-A-Q-E
375 S15 3842.6 F-Q-P-H-P-G-L-Q-K-T-L-E-Q-F-H-L-S-S-M-S-S-
L-G-G-P-A-A-F-S-A-R-W-A-Q-E
376 S16 3832.6 F-Q-P-H-P-G-L-Q-K-T-L-E-Q-F-H-L-S-S-M-S-S-

L-G-G-S-A-A-F-S-A-R-W-A-Q-E
377 S17 3846.6 F-Q-P-H-P-G-L-Q-K-T-L-E-Q-F-H-L-S-S-M-S-S-
L-G-G-T-A-A-F-S-A-R-W-A-Q-E
378 S18 3931.7 F-Q-P-H-P-G-L-Q-K-T-L-E-Q-F-H-L-S-S-M-S-S-
L-G-G-W-A-A-F-S-A-R-W-A-Q-E
379 S19 3908.7 F-Q-P-H-P-G-L-Q-K-T-L-E-Q-F-H-L-S-S-M-S-S-
L-G-G-Y-A-A-F-S-A-R-W-A-Q-E
380 S20 3844.6 F-Q-P-H-P-G-L-Q-K-T-L-E-Q-F-H-L-S-S-M-S-S-
L-G-G-V-A-A-F-S-A-R-W-A-Q-E
381 T 1 0

382 T 2 0

383 T 3 0

384 T 4 0

385 T 5 0

386 T 6 0

387 T 7 0

388 T 8 0

389 T 9 0

390 T10 0

391 T11 0

392 T12 0

393 T13 0

394 T14 0

395 T15 0

396 T16 0

397 T17 0

398 T18 0

399 T19 0

400 T20 0

401 U 1 0

402 U 2 0

403 U 3 0

404 U 4 0

405 U 5 0

406 U 6 0

407 U 7 0

408 U 8 0

409 U 9 0

410 U10 0

411 U11 0

412 U12 0

413 U13 0

414 U14 0

415 U15 0

416 U16 0

417 U17 0

418 U18 0

419 U19 0

420 U20 0

421 V 1 3842.6 F-Q-P-H-P-G-L-Q-K-T-L-E-Q-F-H-L-S-S-M-S-S-
L-G-G-P-A-A-F-S-A-R-W-A-Q-E
422 V 2 3842.6 F-Q-P-H-P-G-L-Q-K-T-L-E-Q-F-H-L-S-S-M-S-S-
L-G-G-P-A-A-F-S-A-R-W-A-Q-E
423 V 3 3842.6 F-Q-P-H-P-G-L-Q-K-T-L-E-Q-F-H-L-S-S-M-S-S-
L-G-G-P-A-A-F-S-A-R-W-A-Q-E
424 V 4 3842.6 F-Q-P-H-P-G-L-Q-K-T-L-E-Q-F-H-L-S-S-M-S-S-
L-G-G-P-A-A-F-S-A-R-W-A-Q-E
425 V 5 3842.6 F-Q-P-H-P-G-L-Q-K-T-L-E-Q-F-H-L-S-S-M-S-S-
L-G-G-P-A-A-F-S-A-R-W-A-Q-E
426 V 6 0

427 V 7 0

428 V 8 3842.6 F-Q-P-H-P-G-L-Q-K-T-L-E-Q-F-H-L-S-S-M-S-S-
L-G-G-P-A-A-F-S-A-R-W-A-Q-E
429 V 9 3842.6 F-Q-P-H-P-G-L-Q-K-T-L-E-Q-F-H-L-S-S-M-S-S-
L-G-G-P-A-A-F-S-A-R-W-A-Q-E
430 V10 3842.6 F-Q-P-H-P-G-L-Q-K-T-L-E-Q-F-H-L-S-S-M-S-S-

L-G-G-P-A-A-F-S-A-R-W-A-Q-E
431 V11 3842.6 F-Q-P-H-P-G-L-Q-K-T-L-E-Q-F-H-L-S-S-M-S-S-L-G-G-P-A-A-F-S-A-R-W-A-Q-E
432 V12 3842.6 F-Q-P-H-P-G-L-Q-K-T-L-E-Q-F-H-L-S-S-M-S-S-L-G-G-P-A-A-F-S-A-R-W-A-Q-E
433 V13 0

434 V14 0

435 V15 3842.6 F-Q-P-H-P-G-L-Q-K-T-L-E-Q-F-H-L-S-S-M-S-S-L-G-G-P-A-A-F-S-A-R-W-A-Q-E
436 V16 3842.6 F-Q-P-H-P-G-L-Q-K-T-L-E-Q-F-H-L-S-S-M-S-S-L-G-G-P-A-A-F-S-A-R-W-A-Q-E
437 V17 3842.6 F-Q-P-H-P-G-L-Q-K-T-L-E-Q-F-H-L-S-S-M-S-S-L-G-G-P-A-A-F-S-A-R-W-A-Q-E
438 V18 3842.6 F-Q-P-H-P-G-L-Q-K-T-L-E-Q-F-H-L-S-S-M-S-S-L-G-G-P-A-A-F-S-A-R-W-A-Q-E
439 V19 3842.6 F-Q-P-H-P-G-L-Q-K-T-L-E-Q-F-H-L-S-S-M-S-S-L-G-G-P-A-A-F-S-A-R-W-A-Q-E
440 V20 0

441 W 1 3842.6 F-Q-P-H-P-G-L-Q-K-T-L-E-Q-F-H-L-S-S-M-S-S-L-G-G-P-A-A-F-S-A-R-W-A-Q-E
442 W 2 3842.6 F-Q-P-H-P-G-L-Q-K-T-L-E-Q-F-H-L-S-S-M-S-S-L-G-G-P-A-A-F-S-A-R-W-A-Q-E
443 W 3 3842.6 F-Q-P-H-P-G-L-Q-K-T-L-E-Q-F-H-L-S-S-M-S-S-L-G-G-P-A-A-F-S-A-R-W-A-Q-E
444 W 4 3842.6 F-Q-P-H-P-G-L-Q-K-T-L-E-Q-F-H-L-S-S-M-S-S-L-G-G-P-A-A-F-S-A-R-W-A-Q-E
445 W 5 3842.6 F-Q-P-H-P-G-L-Q-K-T-L-E-Q-F-H-L-S-S-M-S-S-L-G-G-P-A-A-F-S-A-R-W-A-Q-E
446 W 6 0

447 W 7 0

448 W 8 3842.6 F-Q-P-H-P-G-L-Q-K-T-L-E-Q-F-H-L-S-S-M-S-S-L-G-G-P-A-A-F-S-A-R-W-A-Q-E
449 W 9 3842.6 F-Q-P-H-P-G-L-Q-K-T-L-E-Q-F-H-L-S-S-M-S-S-L-G-G-P-A-A-F-S-A-R-W-A-Q-E
450 W10 3842.6 F-Q-P-H-P-G-L-Q-K-T-L-E-Q-F-H-L-S-S-M-S-S-L-G-G-P-A-A-F-S-A-R-W-A-Q-E
451 W11 3842.6 F-Q-P-H-P-G-L-Q-K-T-L-E-Q-F-H-L-S-S-M-S-S-L-G-G-P-A-A-F-S-A-R-W-A-Q-E
452 W12 3842.6 F-Q-P-H-P-G-L-Q-K-T-L-E-Q-F-H-L-S-S-M-S-S-L-G-G-P-A-A-F-S-A-R-W-A-Q-E
453 W13 0

454 W14 0

455 W15 3842.6 F-Q-P-H-P-G-L-Q-K-T-L-E-Q-F-H-L-S-S-M-S-S-L-G-G-P-A-A-F-S-A-R-W-A-Q-E
456 W16 3842.6 F-Q-P-H-P-G-L-Q-K-T-L-E-Q-F-H-L-S-S-M-S-S-L-G-G-P-A-A-F-S-A-R-W-A-Q-E
457 W17 3842.6 F-Q-P-H-P-G-L-Q-K-T-L-E-Q-F-H-L-S-S-M-S-S-

L-G-G-P-A-A-F-S-A-R-W-A-Q-E
458 W18 3842.6 F-Q-P-H-P-G-L-Q-K-T-L-E-Q-F-H-L-S-S-M-S-S-
L-G-G-P-A-A-F-S-A-R-W-A-Q-E
459 W19 3842.6 F-Q-P-H-P-G-L-Q-K-T-L-E-Q-F-H-L-S-S-M-S-S-
L-G-G-P-A-A-F-S-A-R-W-A-Q-E
460 W20 0

461 X 1 3842.6 F-Q-P-H-P-G-L-Q-K-T-L-E-Q-F-H-L-S-S-M-S-S-
L-G-G-P-A-A-F-S-A-R-W-A-Q-E
462 X 2 3842.6 F-Q-P-H-P-G-L-Q-K-T-L-E-Q-F-H-L-S-S-M-S-S-
L-G-G-P-A-A-F-S-A-R-W-A-Q-E
463 X 3 3842.6 F-Q-P-H-P-G-L-Q-K-T-L-E-Q-F-H-L-S-S-M-S-S-
L-G-G-P-A-A-F-S-A-R-W-A-Q-E
464 X 4 3842.6 F-Q-P-H-P-G-L-Q-K-T-L-E-Q-F-H-L-S-S-M-S-S-
L-G-G-P-A-A-F-S-A-R-W-A-Q-E
465 X 5 3842.6 F-Q-P-H-P-G-L-Q-K-T-L-E-Q-F-H-L-S-S-M-S-S-
L-G-G-P-A-A-F-S-A-R-W-A-Q-E
466 X 6 0

467 X 7 0

468 X 8 3842.6 F-Q-P-H-P-G-L-Q-K-T-L-E-Q-F-H-L-S-S-M-S-S-
L-G-G-P-A-A-F-S-A-R-W-A-Q-E
469 X 9 3842.6 F-Q-P-H-P-G-L-Q-K-T-L-E-Q-F-H-L-S-S-M-S-S-
L-G-G-P-A-A-F-S-A-R-W-A-Q-E
470 X10 3842.6 F-Q-P-H-P-G-L-Q-K-T-L-E-Q-F-H-L-S-S-M-S-S-
L-G-G-P-A-A-F-S-A-R-W-A-Q-E
471 X11 3842.6 F-Q-P-H-P-G-L-Q-K-T-L-E-Q-F-H-L-S-S-M-S-S-
L-G-G-P-A-A-F-S-A-R-W-A-Q-E
472 X12 3842.6 F-Q-P-H-P-G-L-Q-K-T-L-E-Q-F-H-L-S-S-M-S-S-
L-G-G-P-A-A-F-S-A-R-W-A-Q-E
473 X13 0

474 X14 0

475 X15 3842.6 F-Q-P-H-P-G-L-Q-K-T-L-E-Q-F-H-L-S-S-M-S-S-
L-G-G-P-A-A-F-S-A-R-W-A-Q-E
476 X16 3842.6 F-Q-P-H-P-G-L-Q-K-T-L-E-Q-F-H-L-S-S-M-S-S-
L-G-G-P-A-A-F-S-A-R-W-A-Q-E
477 X17 3842.6 F-Q-P-H-P-G-L-Q-K-T-L-E-Q-F-H-L-S-S-M-S-S-
L-G-G-P-A-A-F-S-A-R-W-A-Q-E
478 X18 3842.6 F-Q-P-H-P-G-L-Q-K-T-L-E-Q-F-H-L-S-S-M-S-S-
L-G-G-P-A-A-F-S-A-R-W-A-Q-E
479 X19 3842.6 F-Q-P-H-P-G-L-Q-K-T-L-E-Q-F-H-L-S-S-M-S-S-
L-G-G-P-A-A-F-S-A-R-W-A-Q-E
480 X20 0

481 Y 1 3842.6 F-Q-P-H-P-G-L-Q-K-T-L-E-Q-F-H-L-S-S-M-S-S-
L-G-G-P-A-A-F-S-A-R-W-A-Q-E
482 Y 2 3842.6 F-Q-P-H-P-G-L-Q-K-T-L-E-Q-F-H-L-S-S-M-S-S-
L-G-G-P-A-A-F-S-A-R-W-A-Q-E
483 Y 3 3842.6 F-Q-P-H-P-G-L-Q-K-T-L-E-Q-F-H-L-S-S-M-S-S-
L-G-G-P-A-A-F-S-A-R-W-A-Q-E
484 Y 4 3842.6 F-Q-P-H-P-G-L-Q-K-T-L-E-Q-F-H-L-S-S-M-S-S-

L-G-G-P-A-A-F-S-A-R-W-A-Q-E
485 Y 5 3842.6 F-Q-P-H-P-G-L-Q-K-T-L-E-Q-F-H-L-S-S-M-S-S-
L-G-G-P-A-A-F-S-A-R-W-A-Q-E
486 Y 6 0

487 Y 7 0

488 Y 8 3842.6 F-Q-P-H-P-G-L-Q-K-T-L-E-Q-F-H-L-S-S-M-S-S-
L-G-G-P-A-A-F-S-A-R-W-A-Q-E
489 Y 9 3842.6 F-Q-P-H-P-G-L-Q-K-T-L-E-Q-F-H-L-S-S-M-S-S-
L-G-G-P-A-A-F-S-A-R-W-A-Q-E
490 Y10 3842.6 F-Q-P-H-P-G-L-Q-K-T-L-E-Q-F-H-L-S-S-M-S-S-
L-G-G-P-A-A-F-S-A-R-W-A-Q-E
491 Y11 3842.6 F-Q-P-H-P-G-L-Q-K-T-L-E-Q-F-H-L-S-S-M-S-S-
L-G-G-P-A-A-F-S-A-R-W-A-Q-E
492 Y12 3842.6 F-Q-P-H-P-G-L-Q-K-T-L-E-Q-F-H-L-S-S-M-S-S-
L-G-G-P-A-A-F-S-A-R-W-A-Q-E
493 Y13 0

494 Y14 0

495 Y15 3842.6 F-Q-P-H-P-G-L-Q-K-T-L-E-Q-F-H-L-S-S-M-S-S-
L-G-G-P-A-A-F-S-A-R-W-A-Q-E
496 Y16 3842.6 F-Q-P-H-P-G-L-Q-K-T-L-E-Q-F-H-L-S-S-M-S-S-
L-G-G-P-A-A-F-S-A-R-W-A-Q-E
497 Y17 3842.6 F-Q-P-H-P-G-L-Q-K-T-L-E-Q-F-H-L-S-S-M-S-S-
L-G-G-P-A-A-F-S-A-R-W-A-Q-E
498 Y18 3842.6 F-Q-P-H-P-G-L-Q-K-T-L-E-Q-F-H-L-S-S-M-S-S-
L-G-G-P-A-A-F-S-A-R-W-A-Q-E
499 Y19 3842.6 F-Q-P-H-P-G-L-Q-K-T-L-E-Q-F-H-L-S-S-M-S-S-
L-G-G-P-A-A-F-S-A-R-W-A-Q-E
500 Y20 0

501 Z 1 0

502 Z 2 0

503 Z 3 0

504 Z 4 0

505 Z 5 0

506 Z 6 0

507 Z 7 0

508 Z 8 0

509 Z 9 0

510 Z10 0

511 Z11 0

512 Z12 0

513 Z13 0

514 Z14 0

515 Z15 0

516 Z16 0

517 Z17 0

518 Z18 0

519 Z19 0

520 Z20 0

521 [1 3516.3 T-L-E-Q-F-H-L-S-S-M-S-S-L-G-G-P-A-A-F-S-A-R-W-A-Q-E-A-Y-K-K-E-S
522 [2 3516.3 T-L-E-Q-F-H-L-S-S-M-S-S-L-G-G-P-A-A-F-S-A-R-W-A-Q-E-A-Y-K-K-E-S
523 [3 3516.3 T-L-E-Q-F-H-L-S-S-M-S-S-L-G-G-P-A-A-F-S-A-R-W-A-Q-E-A-Y-K-K-E-S
524 [4 3516.3 T-L-E-Q-F-H-L-S-S-M-S-S-L-G-G-P-A-A-F-S-A-R-W-A-Q-E-A-Y-K-K-E-S
525 [5 3516.3 T-L-E-Q-F-H-L-S-S-M-S-S-L-G-G-P-A-A-F-S-A-R-W-A-Q-E-A-Y-K-K-E-S
526 [6 0

527 [7 0

528 [8 3516.3 T-L-E-Q-F-H-L-S-S-M-S-S-L-G-G-P-A-A-F-S-A-R-W-A-Q-E-A-Y-K-K-E-S
529 [9 3516.3 T-L-E-Q-F-H-L-S-S-M-S-S-L-G-G-P-A-A-F-S-A-R-W-A-Q-E-A-Y-K-K-E-S
530 [10 3516.3 T-L-E-Q-F-H-L-S-S-M-S-S-L-G-G-P-A-A-F-S-A-R-W-A-Q-E-A-Y-K-K-E-S
531 [11 3516.3 T-L-E-Q-F-H-L-S-S-M-S-S-L-G-G-P-A-A-F-S-A-R-W-A-Q-E-A-Y-K-K-E-S
532 [12 3516.3 T-L-E-Q-F-H-L-S-S-M-S-S-L-G-G-P-A-A-F-S-A-R-W-A-Q-E-A-Y-K-K-E-S
533 [13 0

534 [14 0

535 [15 3516.3 T-L-E-Q-F-H-L-S-S-M-S-S-L-G-G-P-A-A-F-S-A-R-W-A-Q-E-A-Y-K-K-E-S
536 [16 3516.3 T-L-E-Q-F-H-L-S-S-M-S-S-L-G-G-P-A-A-F-S-A-R-W-A-Q-E-A-Y-K-K-E-S
537 [17 3516.3 T-L-E-Q-F-H-L-S-S-M-S-S-L-G-G-P-A-A-F-S-A-R-W-A-Q-E-A-Y-K-K-E-S
538 [18 3516.3 T-L-E-Q-F-H-L-S-S-M-S-S-L-G-G-P-A-A-

F-S-A-R-W-A-Q-E-A-Y-K-K-E-S
539 [19 3516.3 T-L-E-Q-F-H-L-S-S-M-S-S-L-G-G-P-A-A-
F-S-A-R-W-A-Q-E-A-Y-K-K-E-S
540 [20 0

541 \ 1 3516.3 T-L-E-Q-F-H-L-S-S-M-S-S-L-G-G-P-A-A-
F-S-A-R-W-A-Q-E-A-Y-K-K-E-S
542 \ 2 3516.3 T-L-E-Q-F-H-L-S-S-M-S-S-L-G-G-P-A-A-
F-S-A-R-W-A-Q-E-A-Y-K-K-E-S
543 \ 3 3516.3 T-L-E-Q-F-H-L-S-S-M-S-S-L-G-G-P-A-A-
F-S-A-R-W-A-Q-E-A-Y-K-K-E-S
544 \ 4 3516.3 T-L-E-Q-F-H-L-S-S-M-S-S-L-G-G-P-A-A-
F-S-A-R-W-A-Q-E-A-Y-K-K-E-S
545 \ 5 3516.3 T-L-E-Q-F-H-L-S-S-M-S-S-L-G-G-P-A-A-
F-S-A-R-W-A-Q-E-A-Y-K-K-E-S
546 \ 6 0

547 \ 7 0

548 \ 8 3516.3 T-L-E-Q-F-H-L-S-S-M-S-S-L-G-G-P-A-A-
F-S-A-R-W-A-Q-E-A-Y-K-K-E-S
549 \ 9 3516.3 T-L-E-Q-F-H-L-S-S-M-S-S-L-G-G-P-A-A-
F-S-A-R-W-A-Q-E-A-Y-K-K-E-S
550 \10 3516.3 T-L-E-Q-F-H-L-S-S-M-S-S-L-G-G-P-A-A-
F-S-A-R-W-A-Q-E-A-Y-K-K-E-S
551 \11 3516.3 T-L-E-Q-F-H-L-S-S-M-S-S-L-G-G-P-A-A-
F-S-A-R-W-A-Q-E-A-Y-K-K-E-S
552 \12 3516.3 T-L-E-Q-F-H-L-S-S-M-S-S-L-G-G-P-A-A-
F-S-A-R-W-A-Q-E-A-Y-K-K-E-S
553 \13 0

554 \14 0

555 \15 3516.3 T-L-E-Q-F-H-L-S-S-M-S-S-L-G-G-P-A-A-
F-S-A-R-W-A-Q-E-A-Y-K-K-E-S
556 \16 3516.3 T-L-E-Q-F-H-L-S-S-M-S-S-L-G-G-P-A-A-
F-S-A-R-W-A-Q-E-A-Y-K-K-E-S
557 \17 3516.3 T-L-E-Q-F-H-L-S-S-M-S-S-L-G-G-P-A-A-
F-S-A-R-W-A-Q-E-A-Y-K-K-E-S
558 \18 3516.3 T-L-E-Q-F-H-L-S-S-M-S-S-L-G-G-P-A-A-
F-S-A-R-W-A-Q-E-A-Y-K-K-E-S
559 \19 3516.3 T-L-E-Q-F-H-L-S-S-M-S-S-L-G-G-P-A-A-
F-S-A-R-W-A-Q-E-A-Y-K-K-E-S
560 \20 0

561] 1 3516.3 T-L-E-Q-F-H-L-S-S-M-S-S-L-G-G-P-A-A-
F-S-A-R-W-A-Q-E-A-Y-K-K-E-S
562] 2 3516.3 T-L-E-Q-F-H-L-S-S-M-S-S-L-G-G-P-A-A-
F-S-A-R-W-A-Q-E-A-Y-K-K-E-S
563] 3 3516.3 T-L-E-Q-F-H-L-S-S-M-S-S-L-G-G-P-A-A-
F-S-A-R-W-A-Q-E-A-Y-K-K-E-S
564] 4 3516.3 T-L-E-Q-F-H-L-S-S-M-S-S-L-G-G-P-A-A-
F-S-A-R-W-A-Q-E-A-Y-K-K-E-S
565] 5 3516.3 T-L-E-Q-F-H-L-S-S-M-S-S-L-G-G-P-A-A-

F-S-A-R-W-A-Q-E-A-Y-K-K-E-S
566] 6 0

567] 7 0

568] 8 3516.3 T-L-E-Q-F-H-L-S-S-M-S-S-L-G-G-P-A-A-
F-S-A-R-W-A-Q-E-A-Y-K-K-E-S
569] 9 3516.3 T-L-E-Q-F-H-L-S-S-M-S-S-L-G-G-P-A-A-
F-S-A-R-W-A-Q-E-A-Y-K-K-E-S
570]10 3516.3 T-L-E-Q-F-H-L-S-S-M-S-S-L-G-G-P-A-A-
F-S-A-R-W-A-Q-E-A-Y-K-K-E-S
571]11 3516.3 T-L-E-Q-F-H-L-S-S-M-S-S-L-G-G-P-A-A-
F-S-A-R-W-A-Q-E-A-Y-K-K-E-S
572]12 3516.3 T-L-E-Q-F-H-L-S-S-M-S-S-L-G-G-P-A-A-
F-S-A-R-W-A-Q-E-A-Y-K-K-E-S
573]13 0

574]14 0

575]15 3516.3 T-L-E-Q-F-H-L-S-S-M-S-S-L-G-G-P-A-A-
F-S-A-R-W-A-Q-E-A-Y-K-K-E-S
576]16 3516.3 T-L-E-Q-F-H-L-S-S-M-S-S-L-G-G-P-A-A-
F-S-A-R-W-A-Q-E-A-Y-K-K-E-S
577]17 3516.3 T-L-E-Q-F-H-L-S-S-M-S-S-L-G-G-P-A-A-
F-S-A-R-W-A-Q-E-A-Y-K-K-E-S
578]18 3516.3 T-L-E-Q-F-H-L-S-S-M-S-S-L-G-G-P-A-A-
F-S-A-R-W-A-Q-E-A-Y-K-K-E-S
579]19 3516.3 T-L-E-Q-F-H-L-S-S-M-S-S-L-G-G-P-A-A-
F-S-A-R-W-A-Q-E-A-Y-K-K-E-S
580]20 0

581 ^ 1 3516.3 T-L-E-Q-F-H-L-S-S-M-S-S-L-G-G-P-A-A-
F-S-A-R-W-A-Q-E-A-Y-K-K-E-S
582 ^ 2 3516.3 T-L-E-Q-F-H-L-S-S-M-S-S-L-G-G-P-A-A-
F-S-A-R-W-A-Q-E-A-Y-K-K-E-S
583 ^ 3 3516.3 T-L-E-Q-F-H-L-S-S-M-S-S-L-G-G-P-A-A-
F-S-A-R-W-A-Q-E-A-Y-K-K-E-S
584 ^ 4 3516.3 T-L-E-Q-F-H-L-S-S-M-S-S-L-G-G-P-A-A-
F-S-A-R-W-A-Q-E-A-Y-K-K-E-S
585 ^ 5 3516.3 T-L-E-Q-F-H-L-S-S-M-S-S-L-G-G-P-A-A-
F-S-A-R-W-A-Q-E-A-Y-K-K-E-S
586 ^ 6 0

587 ^ 7 0

588 ^ 8 3516.3 T-L-E-Q-F-H-L-S-S-M-S-S-L-G-G-P-A-A-
F-S-A-R-W-A-Q-E-A-Y-K-K-E-S
589 ^ 9 3516.3 T-L-E-Q-F-H-L-S-S-M-S-S-L-G-G-P-A-A-
F-S-A-R-W-A-Q-E-A-Y-K-K-E-S
590 ^10 3516.3 T-L-E-Q-F-H-L-S-S-M-S-S-L-G-G-P-A-A-
F-S-A-R-W-A-Q-E-A-Y-K-K-E-S
591 ^11 3516.3 T-L-E-Q-F-H-L-S-S-M-S-S-L-G-G-P-A-A-
F-S-A-R-W-A-Q-E-A-Y-K-K-E-S
592 ^12 3516.3 T-L-E-Q-F-H-L-S-S-M-S-S-L-G-G-P-A-A-

F-S-A-R-W-A-Q-E-A-Y-K-K-E-S
593 ^13 0

594 ^14 0

595 ^15 3516.3 T-L-E-Q-F-H-L-S-S-M-S-S-L-G-G-P-A-A-
F-S-A-R-W-A-Q-E-A-Y-K-K-E-S
596 ^16 3516.3 T-L-E-Q-F-H-L-S-S-M-S-S-L-G-G-P-A-A-
F-S-A-R-W-A-Q-E-A-Y-K-K-E-S
597 ^17 3516.3 T-L-E-Q-F-H-L-S-S-M-S-S-L-G-G-P-A-A-
F-S-A-R-W-A-Q-E-A-Y-K-K-E-S
598 ^18 3516.3 T-L-E-Q-F-H-L-S-S-M-S-S-L-G-G-P-A-A-
F-S-A-R-W-A-Q-E-A-Y-K-K-E-S
599 ^19 3516.3 T-L-E-Q-F-H-L-S-S-M-S-S-L-G-G-P-A-A-
F-S-A-R-W-A-Q-E-A-Y-K-K-E-S
600 ^20 0

601 A 1 3800.5 F-Q-P-H-P-G-A-Q-K-T-L-E-Q-F-H-L-S-S-M-S-S-
L-G-G-P-A-A-F-S-A-R-W-A-Q-E
602 A 2 3885.6 F-Q-P-H-P-G-R-Q-K-T-L-E-Q-F-H-L-S-S-M-S-S-
L-G-G-P-A-A-F-S-A-R-W-A-Q-E
603 A 3 3843.5 F-Q-P-H-P-G-N-Q-K-T-L-E-Q-F-H-L-S-S-M-S-S-
L-G-G-P-A-A-F-S-A-R-W-A-Q-E
604 A 4 3844.5 F-Q-P-H-P-G-D-Q-K-T-L-E-Q-F-H-L-S-S-M-S-S-
L-G-G-P-A-A-F-S-A-R-W-A-Q-E
605 A 5 3832.5 F-Q-P-H-P-G-C-Q-K-T-L-E-Q-F-H-L-S-S-M-S-S-
L-G-G-P-A-A-F-S-A-R-W-A-Q-E
606 A 6 3857.5 F-Q-P-H-P-G-Q-Q-K-T-L-E-Q-F-H-L-S-S-M-S-S-
L-G-G-P-A-A-F-S-A-R-W-A-Q-E
607 A 7 3858.5 F-Q-P-H-P-G-E-Q-K-T-L-E-Q-F-H-L-S-S-M-S-S-
L-G-G-P-A-A-F-S-A-R-W-A-Q-E
608 A 8 3786.5 F-Q-P-H-P-G-G-Q-K-T-L-E-Q-F-H-L-S-S-M-S-S-
L-G-G-P-A-A-F-S-A-R-W-A-Q-E
609 A 9 3866.5 F-Q-P-H-P-G-H-Q-K-T-L-E-Q-F-H-L-S-S-M-S-S-
L-G-G-P-A-A-F-S-A-R-W-A-Q-E
610 A10 3842.6 F-Q-P-H-P-G-I-Q-K-T-L-E-Q-F-H-L-S-S-M-S-S-
L-G-G-P-A-A-F-S-A-R-W-A-Q-E
611 A11 3842.6 F-Q-P-H-P-G-L-Q-K-T-L-E-Q-F-H-L-S-S-M-S-S-
L-G-G-P-A-A-F-S-A-R-W-A-Q-E
612 A12 3857.6 F-Q-P-H-P-G-K-Q-K-T-L-E-Q-F-H-L-S-S-M-S-S-
L-G-G-P-A-A-F-S-A-R-W-A-Q-E
613 A13 3860.6 F-Q-P-H-P-G-M-Q-K-T-L-E-Q-F-H-L-S-S-M-S-S-
L-G-G-P-A-A-F-S-A-R-W-A-Q-E
614 A14 3876.6 F-Q-P-H-P-G-F-Q-K-T-L-E-Q-F-H-L-S-S-M-S-S-
L-G-G-P-A-A-F-S-A-R-W-A-Q-E
615 A15 3826.5 F-Q-P-H-P-G-P-Q-K-T-L-E-Q-F-H-L-S-S-M-S-S-
L-G-G-P-A-A-F-S-A-R-W-A-Q-E
616 A16 3816.5 F-Q-P-H-P-G-S-Q-K-T-L-E-Q-F-H-L-S-S-M-S-S-
L-G-G-P-A-A-F-S-A-R-W-A-Q-E
617 A17 3830.5 F-Q-P-H-P-G-T-Q-K-T-L-E-Q-F-H-L-S-S-M-S-S-
L-G-G-P-A-A-F-S-A-R-W-A-Q-E
618 A18 3915.6 F-Q-P-H-P-G-W-Q-K-T-L-E-Q-F-H-L-S-S-M-S-S-
L-G-G-P-A-A-F-S-A-R-W-A-Q-E
619 A19 3892.6 F-Q-P-H-P-G-Y-Q-K-T-L-E-Q-F-H-L-S-S-M-S-S-

L-G-G-P-A-A-F-S-A-R-W-A-Q-E
620 A20 3828.5 F-Q-P-H-P-G-V-Q-K-T-L-E-Q-F-H-L-S-S-M-S-S-
L-G-G-P-A-A-F-S-A-R-W-A-Q-E
621 B 1 3785.6 F-Q-P-H-P-G-L-A-K-T-L-E-Q-F-H-L-S-S-M-S-S-
L-G-G-P-A-A-F-S-A-R-W-A-Q-E
622 B 2 3870.7 F-Q-P-H-P-G-L-R-K-T-L-E-Q-F-H-L-S-S-M-S-S-
L-G-G-P-A-A-F-S-A-R-W-A-Q-E
623 B 3 3828.6 F-Q-P-H-P-G-L-N-K-T-L-E-Q-F-H-L-S-S-M-S-S-
L-G-G-P-A-A-F-S-A-R-W-A-Q-E
624 B 4 3829.6 F-Q-P-H-P-G-L-D-K-T-L-E-Q-F-H-L-S-S-M-S-S-
L-G-G-P-A-A-F-S-A-R-W-A-Q-E
625 B 5 3817.6 F-Q-P-H-P-G-L-C-K-T-L-E-Q-F-H-L-S-S-M-S-S-
L-G-G-P-A-A-F-S-A-R-W-A-Q-E
626 B 6 3842.6 F-Q-P-H-P-G-L-Q-K-T-L-E-Q-F-H-L-S-S-M-S-S-
L-G-G-P-A-A-F-S-A-R-W-A-Q-E
627 B 7 3843.6 F-Q-P-H-P-G-L-E-K-T-L-E-Q-F-H-L-S-S-M-S-S-
L-G-G-P-A-A-F-S-A-R-W-A-Q-E
628 B 8 3771.6 F-Q-P-H-P-G-L-G-K-T-L-E-Q-F-H-L-S-S-M-S-S-
L-G-G-P-A-A-F-S-A-R-W-A-Q-E
629 B 9 3851.6 F-Q-P-H-P-G-L-H-K-T-L-E-Q-F-H-L-S-S-M-S-S-
L-G-G-P-A-A-F-S-A-R-W-A-Q-E
630 B10 3827.7 F-Q-P-H-P-G-L-I-K-T-L-E-Q-F-H-L-S-S-M-S-S-
L-G-G-P-A-A-F-S-A-R-W-A-Q-E
631 B11 3827.7 F-Q-P-H-P-G-L-L-K-T-L-E-Q-F-H-L-S-S-M-S-S-
L-G-G-P-A-A-F-S-A-R-W-A-Q-E
632 B12 3842.7 F-Q-P-H-P-G-L-K-K-T-L-E-Q-F-H-L-S-S-M-S-S-
L-G-G-P-A-A-F-S-A-R-W-A-Q-E
633 B13 3845.7 F-Q-P-H-P-G-L-M-K-T-L-E-Q-F-H-L-S-S-M-S-S-
L-G-G-P-A-A-F-S-A-R-W-A-Q-E
634 B14 3861.7 F-Q-P-H-P-G-L-F-K-T-L-E-Q-F-H-L-S-S-M-S-S-
L-G-G-P-A-A-F-S-A-R-W-A-Q-E
635 B15 3811.6 F-Q-P-H-P-G-L-P-K-T-L-E-Q-F-H-L-S-S-M-S-S-
L-G-G-P-A-A-F-S-A-R-W-A-Q-E
636 B16 3801.6 F-Q-P-H-P-G-L-S-K-T-L-E-Q-F-H-L-S-S-M-S-S-
L-G-G-P-A-A-F-S-A-R-W-A-Q-E
637 B17 3815.6 F-Q-P-H-P-G-L-T-K-T-L-E-Q-F-H-L-S-S-M-S-S-
L-G-G-P-A-A-F-S-A-R-W-A-Q-E
638 B18 3900.7 F-Q-P-H-P-G-L-W-K-T-L-E-Q-F-H-L-S-S-M-S-S-
L-G-G-P-A-A-F-S-A-R-W-A-Q-E
639 B19 3877.7 F-Q-P-H-P-G-L-Y-K-T-L-E-Q-F-H-L-S-S-M-S-S-
L-G-G-P-A-A-F-S-A-R-W-A-Q-E
640 B20 3813.6 F-Q-P-H-P-G-L-V-K-T-L-E-Q-F-H-L-S-S-M-S-S-
L-G-G-P-A-A-F-S-A-R-W-A-Q-E
641 C 1 3785.5 F-Q-P-H-P-G-L-Q-A-T-L-E-Q-F-H-L-S-S-M-S-S-
L-G-G-P-A-A-F-S-A-R-W-A-Q-E
642 C 2 3870.6 F-Q-P-H-P-G-L-Q-R-T-L-E-Q-F-H-L-S-S-M-S-S-
L-G-G-P-A-A-F-S-A-R-W-A-Q-E
643 C 3 3828.5 F-Q-P-H-P-G-L-Q-N-T-L-E-Q-F-H-L-S-S-M-S-S-
L-G-G-P-A-A-F-S-A-R-W-A-Q-E
644 C 4 3829.5 F-Q-P-H-P-G-L-Q-D-T-L-E-Q-F-H-L-S-S-M-S-S-
L-G-G-P-A-A-F-S-A-R-W-A-Q-E
645 C 5 3817.5 F-Q-P-H-P-G-L-Q-C-T-L-E-Q-F-H-L-S-S-M-S-S-
L-G-G-P-A-A-F-S-A-R-W-A-Q-E
646 C 6 3842.5 F-Q-P-H-P-G-L-Q-Q-T-L-E-Q-F-H-L-S-S-M-S-S-

L-G-G-P-A-A-F-S-A-R-W-A-Q-E
647 C 7 3843.5 F-Q-P-H-P-G-L-Q-E-T-L-E-Q-F-H-L-S-S-M-S-S-
L-G-G-P-A-A-F-S-A-R-W-A-Q-E
648 C 8 3771.5 F-Q-P-H-P-G-L-Q-G-T-L-E-Q-F-H-L-S-S-M-S-S-
L-G-G-P-A-A-F-S-A-R-W-A-Q-E
649 C 9 3851.5 F-Q-P-H-P-G-L-Q-H-T-L-E-Q-F-H-L-S-S-M-S-S-
L-G-G-P-A-A-F-S-A-R-W-A-Q-E
650 C10 3827.6 F-Q-P-H-P-G-L-Q-I-T-L-E-Q-F-H-L-S-S-M-S-S-
L-G-G-P-A-A-F-S-A-R-W-A-Q-E
651 C11 3827.6 F-Q-P-H-P-G-L-Q-L-T-L-E-Q-F-H-L-S-S-M-S-S-
L-G-G-P-A-A-F-S-A-R-W-A-Q-E
652 C12 3842.6 F-Q-P-H-P-G-L-Q-K-T-L-E-Q-F-H-L-S-S-M-S-S-
L-G-G-P-A-A-F-S-A-R-W-A-Q-E
653 C13 3845.6 F-Q-P-H-P-G-L-Q-M-T-L-E-Q-F-H-L-S-S-M-S-S-
L-G-G-P-A-A-F-S-A-R-W-A-Q-E
654 C14 3861.6 F-Q-P-H-P-G-L-Q-F-T-L-E-Q-F-H-L-S-S-M-S-S-
L-G-G-P-A-A-F-S-A-R-W-A-Q-E
655 C15 3811.5 F-Q-P-H-P-G-L-Q-P-T-L-E-Q-F-H-L-S-S-M-S-S-
L-G-G-P-A-A-F-S-A-R-W-A-Q-E
656 C16 3801.5 F-Q-P-H-P-G-L-Q-S-T-L-E-Q-F-H-L-S-S-M-S-S-
L-G-G-P-A-A-F-S-A-R-W-A-Q-E
657 C17 3815.5 F-Q-P-H-P-G-L-Q-T-T-L-E-Q-F-H-L-S-S-M-S-S-
L-G-G-P-A-A-F-S-A-R-W-A-Q-E
658 C18 3900.6 F-Q-P-H-P-G-L-Q-W-T-L-E-Q-F-H-L-S-S-M-S-S-
L-G-G-P-A-A-F-S-A-R-W-A-Q-E
659 C19 3877.6 F-Q-P-H-P-G-L-Q-Y-T-L-E-Q-F-H-L-S-S-M-S-S-
L-G-G-P-A-A-F-S-A-R-W-A-Q-E
660 C20 3813.5 F-Q-P-H-P-G-L-Q-V-T-L-E-Q-F-H-L-S-S-M-S-S-
L-G-G-P-A-A-F-S-A-R-W-A-Q-E
661 D 1 3812.6 F-Q-P-H-P-G-L-Q-K-A-L-E-Q-F-H-L-S-S-M-S-S-
L-G-G-P-A-A-F-S-A-R-W-A-Q-E
662 D 2 3897.7 F-Q-P-H-P-G-L-Q-K-R-L-E-Q-F-H-L-S-S-M-S-S-
L-G-G-P-A-A-F-S-A-R-W-A-Q-E
663 D 3 3855.6 F-Q-P-H-P-G-L-Q-K-N-L-E-Q-F-H-L-S-S-M-S-S-
L-G-G-P-A-A-F-S-A-R-W-A-Q-E
664 D 4 3856.6 F-Q-P-H-P-G-L-Q-K-D-L-E-Q-F-H-L-S-S-M-S-S-
L-G-G-P-A-A-F-S-A-R-W-A-Q-E
665 D 5 3844.6 F-Q-P-H-P-G-L-Q-K-C-L-E-Q-F-H-L-S-S-M-S-S-
L-G-G-P-A-A-F-S-A-R-W-A-Q-E
666 D 6 3869.6 F-Q-P-H-P-G-L-Q-K-Q-L-E-Q-F-H-L-S-S-M-S-S-
L-G-G-P-A-A-F-S-A-R-W-A-Q-E
667 D 7 3870.6 F-Q-P-H-P-G-L-Q-K-E-L-E-Q-F-H-L-S-S-M-S-S-
L-G-G-P-A-A-F-S-A-R-W-A-Q-E
668 D 8 3798.6 F-Q-P-H-P-G-L-Q-K-G-L-E-Q-F-H-L-S-S-M-S-S-
L-G-G-P-A-A-F-S-A-R-W-A-Q-E
669 D 9 3878.6 F-Q-P-H-P-G-L-Q-K-H-L-E-Q-F-H-L-S-S-M-S-S-
L-G-G-P-A-A-F-S-A-R-W-A-Q-E
670 D10 3854.7 F-Q-P-H-P-G-L-Q-K-I-L-E-Q-F-H-L-S-S-M-S-S-
L-G-G-P-A-A-F-S-A-R-W-A-Q-E
671 D11 3854.7 F-Q-P-H-P-G-L-Q-K-L-L-E-Q-F-H-L-S-S-M-S-S-
L-G-G-P-A-A-F-S-A-R-W-A-Q-E
672 D12 3869.7 F-Q-P-H-P-G-L-Q-K-K-L-E-Q-F-H-L-S-S-M-S-S-
L-G-G-P-A-A-F-S-A-R-W-A-Q-E
673 D13 3872.7 F-Q-P-H-P-G-L-Q-K-M-L-E-Q-F-H-L-S-S-M-S-S-

L-G-G-P-A-A-F-S-A-R-W-A-Q-E
674 D14 3888.7 F-Q-P-H-P-G-L-Q-K-F-L-E-Q-F-H-L-S-S-M-S-S-
L-G-G-P-A-A-F-S-A-R-W-A-Q-E
675 D15 3838.6 F-Q-P-H-P-G-L-Q-K-P-L-E-Q-F-H-L-S-S-M-S-S-
L-G-G-P-A-A-F-S-A-R-W-A-Q-E
676 D16 3828.6 F-Q-P-H-P-G-L-Q-K-S-L-E-Q-F-H-L-S-S-M-S-S-
L-G-G-P-A-A-F-S-A-R-W-A-Q-E
677 D17 3842.6 F-Q-P-H-P-G-L-Q-K-T-L-E-Q-F-H-L-S-S-M-S-S-
L-G-G-P-A-A-F-S-A-R-W-A-Q-E
678 D18 3927.7 F-Q-P-H-P-G-L-Q-K-W-L-E-Q-F-H-L-S-S-M-S-S-
L-G-G-P-A-A-F-S-A-R-W-A-Q-E
679 D19 3904.7 F-Q-P-H-P-G-L-Q-K-Y-L-E-Q-F-H-L-S-S-M-S-S-
L-G-G-P-A-A-F-S-A-R-W-A-Q-E
680 D20 3840.6 F-Q-P-H-P-G-L-Q-K-V-L-E-Q-F-H-L-S-S-M-S-S-
L-G-G-P-A-A-F-S-A-R-W-A-Q-E
681 E 1 3800.5 F-Q-P-H-P-G-L-Q-K-T-A-E-Q-F-H-L-S-S-M-S-S-
L-G-G-P-A-A-F-S-A-R-W-A-Q-E
682 E 2 3885.6 F-Q-P-H-P-G-L-Q-K-T-R-E-Q-F-H-L-S-S-M-S-S-
L-G-G-P-A-A-F-S-A-R-W-A-Q-E
683 E 3 3843.5 F-Q-P-H-P-G-L-Q-K-T-N-E-Q-F-H-L-S-S-M-S-S-
L-G-G-P-A-A-F-S-A-R-W-A-Q-E
684 E 4 3844.5 F-Q-P-H-P-G-L-Q-K-T-D-E-Q-F-H-L-S-S-M-S-S-
L-G-G-P-A-A-F-S-A-R-W-A-Q-E
685 E 5 3832.5 F-Q-P-H-P-G-L-Q-K-T-C-E-Q-F-H-L-S-S-M-S-S-
L-G-G-P-A-A-F-S-A-R-W-A-Q-E
686 E 6 3857.5 F-Q-P-H-P-G-L-Q-K-T-Q-E-Q-F-H-L-S-S-M-S-S-
L-G-G-P-A-A-F-S-A-R-W-A-Q-E
687 E 7 3858.5 F-Q-P-H-P-G-L-Q-K-T-E-E-Q-F-H-L-S-S-M-S-S-
L-G-G-P-A-A-F-S-A-R-W-A-Q-E
688 E 8 3786.5 F-Q-P-H-P-G-L-Q-K-T-G-E-Q-F-H-L-S-S-M-S-S-
L-G-G-P-A-A-F-S-A-R-W-A-Q-E
689 E 9 3866.5 F-Q-P-H-P-G-L-Q-K-T-H-E-Q-F-H-L-S-S-M-S-S-
L-G-G-P-A-A-F-S-A-R-W-A-Q-E
690 E10 3842.6 F-Q-P-H-P-G-L-Q-K-T-I-E-Q-F-H-L-S-S-M-S-S-
L-G-G-P-A-A-F-S-A-R-W-A-Q-E
691 E11 3842.6 F-Q-P-H-P-G-L-Q-K-T-L-E-Q-F-H-L-S-S-M-S-S-
L-G-G-P-A-A-F-S-A-R-W-A-Q-E
692 E12 3857.6 F-Q-P-H-P-G-L-Q-K-T-K-E-Q-F-H-L-S-S-M-S-S-
L-G-G-P-A-A-F-S-A-R-W-A-Q-E
693 E13 3860.6 F-Q-P-H-P-G-L-Q-K-T-M-E-Q-F-H-L-S-S-M-S-S-
L-G-G-P-A-A-F-S-A-R-W-A-Q-E
694 E14 3876.6 F-Q-P-H-P-G-L-Q-K-T-F-E-Q-F-H-L-S-S-M-S-S-
L-G-G-P-A-A-F-S-A-R-W-A-Q-E
695 E15 3826.5 F-Q-P-H-P-G-L-Q-K-T-P-E-Q-F-H-L-S-S-M-S-S-
L-G-G-P-A-A-F-S-A-R-W-A-Q-E
696 E16 3816.5 F-Q-P-H-P-G-L-Q-K-T-S-E-Q-F-H-L-S-S-M-S-S-
L-G-G-P-A-A-F-S-A-R-W-A-Q-E
697 E17 3830.5 F-Q-P-H-P-G-L-Q-K-T-T-E-Q-F-H-L-S-S-M-S-S-
L-G-G-P-A-A-F-S-A-R-W-A-Q-E
698 E18 3915.6 F-Q-P-H-P-G-L-Q-K-T-W-E-Q-F-H-L-S-S-M-S-S-
L-G-G-P-A-A-F-S-A-R-W-A-Q-E
699 E19 3892.6 F-Q-P-H-P-G-L-Q-K-T-Y-E-Q-F-H-L-S-S-M-S-S-
L-G-G-P-A-A-F-S-A-R-W-A-Q-E
700 E20 3828.5 F-Q-P-H-P-G-L-Q-K-T-V-E-Q-F-H-L-S-S-M-S-S-

L-G-G-P-A-A-F-S-A-R-W-A-Q-E
701 F 1 3784.6 F-Q-P-H-P-G-L-Q-K-T-L-A-Q-F-H-L-S-S-M-S-S-
L-G-G-P-A-A-F-S-A-R-W-A-Q-E
702 F 2 3869.7 F-Q-P-H-P-G-L-Q-K-T-L-R-Q-F-H-L-S-S-M-S-S-
L-G-G-P-A-A-F-S-A-R-W-A-Q-E
703 F 3 3827.6 F-Q-P-H-P-G-L-Q-K-T-L-N-Q-F-H-L-S-S-M-S-S-
L-G-G-P-A-A-F-S-A-R-W-A-Q-E
704 F 4 3828.6 F-Q-P-H-P-G-L-Q-K-T-L-D-Q-F-H-L-S-S-M-S-S-
L-G-G-P-A-A-F-S-A-R-W-A-Q-E
705 F 5 3816.6 F-Q-P-H-P-G-L-Q-K-T-L-C-Q-F-H-L-S-S-M-S-S-
L-G-G-P-A-A-F-S-A-R-W-A-Q-E
706 F 6 3841.6 F-Q-P-H-P-G-L-Q-K-T-L-Q-Q-F-H-L-S-S-M-S-S-
L-G-G-P-A-A-F-S-A-R-W-A-Q-E
707 F 7 3842.6 F-Q-P-H-P-G-L-Q-K-T-L-E-Q-F-H-L-S-S-M-S-S-
L-G-G-P-A-A-F-S-A-R-W-A-Q-E
708 F 8 3770.6 F-Q-P-H-P-G-L-Q-K-T-L-G-Q-F-H-L-S-S-M-S-S-
L-G-G-P-A-A-F-S-A-R-W-A-Q-E
709 F 9 3850.6 F-Q-P-H-P-G-L-Q-K-T-L-H-Q-F-H-L-S-S-M-S-S-
L-G-G-P-A-A-F-S-A-R-W-A-Q-E
710 F10 3826.7 F-Q-P-H-P-G-L-Q-K-T-L-I-Q-F-H-L-S-S-M-S-S-
L-G-G-P-A-A-F-S-A-R-W-A-Q-E
711 F11 3826.7 F-Q-P-H-P-G-L-Q-K-T-L-L-Q-F-H-L-S-S-M-S-S-
L-G-G-P-A-A-F-S-A-R-W-A-Q-E
712 F12 3841.7 F-Q-P-H-P-G-L-Q-K-T-L-K-Q-F-H-L-S-S-M-S-S-
L-G-G-P-A-A-F-S-A-R-W-A-Q-E
713 F13 3844.7 F-Q-P-H-P-G-L-Q-K-T-L-M-Q-F-H-L-S-S-M-S-S-
L-G-G-P-A-A-F-S-A-R-W-A-Q-E
714 F14 3860.7 F-Q-P-H-P-G-L-Q-K-T-L-F-Q-F-H-L-S-S-M-S-S-
L-G-G-P-A-A-F-S-A-R-W-A-Q-E
715 F15 3810.6 F-Q-P-H-P-G-L-Q-K-T-L-P-Q-F-H-L-S-S-M-S-S-
L-G-G-P-A-A-F-S-A-R-W-A-Q-E
716 F16 3800.6 F-Q-P-H-P-G-L-Q-K-T-L-S-Q-F-H-L-S-S-M-S-S-
L-G-G-P-A-A-F-S-A-R-W-A-Q-E
717 F17 3814.6 F-Q-P-H-P-G-L-Q-K-T-L-T-Q-F-H-L-S-S-M-S-S-
L-G-G-P-A-A-F-S-A-R-W-A-Q-E
718 F18 3899.7 F-Q-P-H-P-G-L-Q-K-T-L-W-Q-F-H-L-S-S-M-S-S-
L-G-G-P-A-A-F-S-A-R-W-A-Q-E
719 F19 3876.7 F-Q-P-H-P-G-L-Q-K-T-L-Y-Q-F-H-L-S-S-M-S-S-
L-G-G-P-A-A-F-S-A-R-W-A-Q-E
720 F20 3812.6 F-Q-P-H-P-G-L-Q-K-T-L-V-Q-F-H-L-S-S-M-S-S-
L-G-G-P-A-A-F-S-A-R-W-A-Q-E
721 G 1 3785.6 F-Q-P-H-P-G-L-Q-K-T-L-E-A-F-H-L-S-S-M-S-S-
L-G-G-P-A-A-F-S-A-R-W-A-Q-E
722 G 2 3870.7 F-Q-P-H-P-G-L-Q-K-T-L-E-R-F-H-L-S-S-M-S-S-
L-G-G-P-A-A-F-S-A-R-W-A-Q-E
723 G 3 3828.6 F-Q-P-H-P-G-L-Q-K-T-L-E-N-F-H-L-S-S-M-S-S-
L-G-G-P-A-A-F-S-A-R-W-A-Q-E
724 G 4 3829.6 F-Q-P-H-P-G-L-Q-K-T-L-E-D-F-H-L-S-S-M-S-S-
L-G-G-P-A-A-F-S-A-R-W-A-Q-E
725 G 5 3817.6 F-Q-P-H-P-G-L-Q-K-T-L-E-C-F-H-L-S-S-M-S-S-
L-G-G-P-A-A-F-S-A-R-W-A-Q-E
726 G 6 3842.6 F-Q-P-H-P-G-L-Q-K-T-L-E-Q-F-H-L-S-S-M-S-S-
L-G-G-P-A-A-F-S-A-R-W-A-Q-E
727 G 7 3843.6 F-Q-P-H-P-G-L-Q-K-T-L-E-E-F-H-L-S-S-M-S-S-

L-G-G-P-A-A-F-S-A-R-W-A-Q-E
728 G 8 3771.6 F-Q-P-H-P-G-L-Q-K-T-L-E-G-F-H-L-S-S-M-S-S-
L-G-G-P-A-A-F-S-A-R-W-A-Q-E
729 G 9 3851.6 F-Q-P-H-P-G-L-Q-K-T-L-E-H-F-H-L-S-S-M-S-S-
L-G-G-P-A-A-F-S-A-R-W-A-Q-E
730 G10 3827.7 F-Q-P-H-P-G-L-Q-K-T-L-E-I-F-H-L-S-S-M-S-S-
L-G-G-P-A-A-F-S-A-R-W-A-Q-E
731 G11 3827.7 F-Q-P-H-P-G-L-Q-K-T-L-E-L-F-H-L-S-S-M-S-S-
L-G-G-P-A-A-F-S-A-R-W-A-Q-E
732 G12 3842.7 F-Q-P-H-P-G-L-Q-K-T-L-E-K-F-H-L-S-S-M-S-S-
L-G-G-P-A-A-F-S-A-R-W-A-Q-E
733 G13 3845.7 F-Q-P-H-P-G-L-Q-K-T-L-E-M-F-H-L-S-S-M-S-S-
L-G-G-P-A-A-F-S-A-R-W-A-Q-E
734 G14 3861.7 F-Q-P-H-P-G-L-Q-K-T-L-E-F-F-H-L-S-S-M-S-S-
L-G-G-P-A-A-F-S-A-R-W-A-Q-E
735 G15 3811.6 F-Q-P-H-P-G-L-Q-K-T-L-E-P-F-H-L-S-S-M-S-S-
L-G-G-P-A-A-F-S-A-R-W-A-Q-E
736 G16 3801.6 F-Q-P-H-P-G-L-Q-K-T-L-E-S-F-H-L-S-S-M-S-S-
L-G-G-P-A-A-F-S-A-R-W-A-Q-E
737 G17 3815.6 F-Q-P-H-P-G-L-Q-K-T-L-E-T-F-H-L-S-S-M-S-S-
L-G-G-P-A-A-F-S-A-R-W-A-Q-E
738 G18 3900.7 F-Q-P-H-P-G-L-Q-K-T-L-E-W-F-H-L-S-S-M-S-S-
L-G-G-P-A-A-F-S-A-R-W-A-Q-E
739 G19 3877.7 F-Q-P-H-P-G-L-Q-K-T-L-E-Y-F-H-L-S-S-M-S-S-
L-G-G-P-A-A-F-S-A-R-W-A-Q-E
740 G20 3813.6 F-Q-P-H-P-G-L-Q-K-T-L-E-V-F-H-L-S-S-M-S-S-
L-G-G-P-A-A-F-S-A-R-W-A-Q-E
741 H 1 3766.5 F-Q-P-H-P-G-L-Q-K-T-L-E-Q-A-H-L-S-S-M-S-S-
L-G-G-P-A-A-F-S-A-R-W-A-Q-E
742 H 2 3851.6 F-Q-P-H-P-G-L-Q-K-T-L-E-Q-R-H-L-S-S-M-S-S-
L-G-G-P-A-A-F-S-A-R-W-A-Q-E
743 H 3 3809.5 F-Q-P-H-P-G-L-Q-K-T-L-E-Q-N-H-L-S-S-M-S-S-
L-G-G-P-A-A-F-S-A-R-W-A-Q-E
744 H 4 3810.5 F-Q-P-H-P-G-L-Q-K-T-L-E-Q-D-H-L-S-S-M-S-S-
L-G-G-P-A-A-F-S-A-R-W-A-Q-E
745 H 5 3798.5 F-Q-P-H-P-G-L-Q-K-T-L-E-Q-C-H-L-S-S-M-S-S-
L-G-G-P-A-A-F-S-A-R-W-A-Q-E
746 H 6 3823.5 F-Q-P-H-P-G-L-Q-K-T-L-E-Q-Q-H-L-S-S-M-S-S-
L-G-G-P-A-A-F-S-A-R-W-A-Q-E
747 H 7 3824.5 F-Q-P-H-P-G-L-Q-K-T-L-E-Q-E-H-L-S-S-M-S-S-
L-G-G-P-A-A-F-S-A-R-W-A-Q-E
748 H 8 3752.5 F-Q-P-H-P-G-L-Q-K-T-L-E-Q-G-H-L-S-S-M-S-S-
L-G-G-P-A-A-F-S-A-R-W-A-Q-E
749 H 9 3832.5 F-Q-P-H-P-G-L-Q-K-T-L-E-Q-H-H-L-S-S-M-S-S-
L-G-G-P-A-A-F-S-A-R-W-A-Q-E
750 H10 3808.6 F-Q-P-H-P-G-L-Q-K-T-L-E-Q-I-H-L-S-S-M-S-S-
L-G-G-P-A-A-F-S-A-R-W-A-Q-E
751 H11 3808.6 F-Q-P-H-P-G-L-Q-K-T-L-E-Q-L-H-L-S-S-M-S-S-
L-G-G-P-A-A-F-S-A-R-W-A-Q-E
752 H12 3823.6 F-Q-P-H-P-G-L-Q-K-T-L-E-Q-K-H-L-S-S-M-S-S-
L-G-G-P-A-A-F-S-A-R-W-A-Q-E
753 H13 3826.6 F-Q-P-H-P-G-L-Q-K-T-L-E-Q-M-H-L-S-S-M-S-S-
L-G-G-P-A-A-F-S-A-R-W-A-Q-E
754 H14 3842.6 F-Q-P-H-P-G-L-Q-K-T-L-E-Q-F-H-L-S-S-M-S-S-

L-G-G-P-A-A-F-S-A-R-W-A-Q-E
755 H15 3792.5 F-Q-P-H-P-G-L-Q-K-T-L-E-Q-P-H-L-S-S-M-S-S-L-G-G-P-A-A-F-S-A-R-W-A-Q-E
756 H16 3782.5 F-Q-P-H-P-G-L-Q-K-T-L-E-Q-S-H-L-S-S-M-S-S-L-G-G-P-A-A-F-S-A-R-W-A-Q-E
757 H17 3796.5 F-Q-P-H-P-G-L-Q-K-T-L-E-Q-T-H-L-S-S-M-S-S-L-G-G-P-A-A-F-S-A-R-W-A-Q-E
758 H18 3881.6 F-Q-P-H-P-G-L-Q-K-T-L-E-Q-W-H-L-S-S-M-S-S-L-G-G-P-A-A-F-S-A-R-W-A-Q-E
759 H19 3858.6 F-Q-P-H-P-G-L-Q-K-T-L-E-Q-Y-H-L-S-S-M-S-S-L-G-G-P-A-A-F-S-A-R-W-A-Q-E
760 H20 3794.5 F-Q-P-H-P-G-L-Q-K-T-L-E-Q-V-H-L-S-S-M-S-S-L-G-G-P-A-A-F-S-A-R-W-A-Q-E
761 I 1 3776.6 F-Q-P-H-P-G-L-Q-K-T-L-E-Q-F-A-L-S-S-M-S-S-L-G-G-P-A-A-F-S-A-R-W-A-Q-E
762 I 2 3861.7 F-Q-P-H-P-G-L-Q-K-T-L-E-Q-F-R-L-S-S-M-S-S-L-G-G-P-A-A-F-S-A-R-W-A-Q-E
763 I 3 3819.6 F-Q-P-H-P-G-L-Q-K-T-L-E-Q-F-N-L-S-S-M-S-S-L-G-G-P-A-A-F-S-A-R-W-A-Q-E
764 I 4 3820.6 F-Q-P-H-P-G-L-Q-K-T-L-E-Q-F-D-L-S-S-M-S-S-L-G-G-P-A-A-F-S-A-R-W-A-Q-E
765 I 5 3808.6 F-Q-P-H-P-G-L-Q-K-T-L-E-Q-F-C-L-S-S-M-S-S-L-G-G-P-A-A-F-S-A-R-W-A-Q-E
766 I 6 3833.6 F-Q-P-H-P-G-L-Q-K-T-L-E-Q-F-Q-L-S-S-M-S-S-L-G-G-P-A-A-F-S-A-R-W-A-Q-E
767 I 7 3834.6 F-Q-P-H-P-G-L-Q-K-T-L-E-Q-F-E-L-S-S-M-S-S-L-G-G-P-A-A-F-S-A-R-W-A-Q-E
768 I 8 3762.6 F-Q-P-H-P-G-L-Q-K-T-L-E-Q-F-G-L-S-S-M-S-S-L-G-G-P-A-A-F-S-A-R-W-A-Q-E
769 I 9 3842.6 F-Q-P-H-P-G-L-Q-K-T-L-E-Q-F-H-L-S-S-M-S-S-L-G-G-P-A-A-F-S-A-R-W-A-Q-E
770 I10 3818.7 F-Q-P-H-P-G-L-Q-K-T-L-E-Q-F-I-L-S-S-M-S-S-L-G-G-P-A-A-F-S-A-R-W-A-Q-E
771 I11 3818.7 F-Q-P-H-P-G-L-Q-K-T-L-E-Q-F-L-L-S-S-M-S-S-L-G-G-P-A-A-F-S-A-R-W-A-Q-E
772 I12 3833.7 F-Q-P-H-P-G-L-Q-K-T-L-E-Q-F-K-L-S-S-M-S-S-L-G-G-P-A-A-F-S-A-R-W-A-Q-E
773 I13 3836.7 F-Q-P-H-P-G-L-Q-K-T-L-E-Q-F-M-L-S-S-M-S-S-L-G-G-P-A-A-F-S-A-R-W-A-Q-E
774 I14 3852.7 F-Q-P-H-P-G-L-Q-K-T-L-E-Q-F-F-L-S-S-M-S-S-L-G-G-P-A-A-F-S-A-R-W-A-Q-E
775 I15 3802.6 F-Q-P-H-P-G-L-Q-K-T-L-E-Q-F-P-L-S-S-M-S-S-L-G-G-P-A-A-F-S-A-R-W-A-Q-E
776 I16 3792.6 F-Q-P-H-P-G-L-Q-K-T-L-E-Q-F-S-L-S-S-M-S-S-L-G-G-P-A-A-F-S-A-R-W-A-Q-E
777 I17 3806.6 F-Q-P-H-P-G-L-Q-K-T-L-E-Q-F-T-L-S-S-M-S-S-L-G-G-P-A-A-F-S-A-R-W-A-Q-E
778 I18 3891.7 F-Q-P-H-P-G-L-Q-K-T-L-E-Q-F-W-L-S-S-M-S-S-L-G-G-P-A-A-F-S-A-R-W-A-Q-E
779 I19 3868.7 F-Q-P-H-P-G-L-Q-K-T-L-E-Q-F-Y-L-S-S-M-S-S-L-G-G-P-A-A-F-S-A-R-W-A-Q-E
780 I20 3804.6 F-Q-P-H-P-G-L-Q-K-T-L-E-Q-F-V-L-S-S-M-S-S-L-G-G-P-A-A-F-S-A-R-W-A-Q-E
781 J 1 3800.5 F-Q-P-H-P-G-L-Q-K-T-L-E-Q-F-H-A-S-S-M-S-S-

L-G-G-P-A-A-F-S-A-R-W-A-Q-E
782 J 2 3885.6 F-Q-P-H-P-G-L-Q-K-T-L-E-Q-F-H-R-S-S-M-S-S-
L-G-G-P-A-A-F-S-A-R-W-A-Q-E
783 J 3 3843.5 F-Q-P-H-P-G-L-Q-K-T-L-E-Q-F-H-N-S-S-M-S-S-
L-G-G-P-A-A-F-S-A-R-W-A-Q-E
784 J 4 3844.5 F-Q-P-H-P-G-L-Q-K-T-L-E-Q-F-H-D-S-S-M-S-S-
L-G-G-P-A-A-F-S-A-R-W-A-Q-E
785 J 5 3832.5 F-Q-P-H-P-G-L-Q-K-T-L-E-Q-F-H-C-S-S-M-S-S-
L-G-G-P-A-A-F-S-A-R-W-A-Q-E
786 J 6 3857.5 F-Q-P-H-P-G-L-Q-K-T-L-E-Q-F-H-Q-S-S-M-S-S-
L-G-G-P-A-A-F-S-A-R-W-A-Q-E
787 J 7 3858.5 F-Q-P-H-P-G-L-Q-K-T-L-E-Q-F-H-E-S-S-M-S-S-
L-G-G-P-A-A-F-S-A-R-W-A-Q-E
788 J 8 3786.5 F-Q-P-H-P-G-L-Q-K-T-L-E-Q-F-H-G-S-S-M-S-S-
L-G-G-P-A-A-F-S-A-R-W-A-Q-E
789 J 9 3866.5 F-Q-P-H-P-G-L-Q-K-T-L-E-Q-F-H-H-S-S-M-S-S-
L-G-G-P-A-A-F-S-A-R-W-A-Q-E
790 J10 3842.6 F-Q-P-H-P-G-L-Q-K-T-L-E-Q-F-H-I-S-S-M-S-S-
L-G-G-P-A-A-F-S-A-R-W-A-Q-E
791 J11 3842.6 F-Q-P-H-P-G-L-Q-K-T-L-E-Q-F-H-L-S-S-M-S-S-
L-G-G-P-A-A-F-S-A-R-W-A-Q-E
792 J12 3857.6 F-Q-P-H-P-G-L-Q-K-T-L-E-Q-F-H-K-S-S-M-S-S-
L-G-G-P-A-A-F-S-A-R-W-A-Q-E
793 J13 3860.6 F-Q-P-H-P-G-L-Q-K-T-L-E-Q-F-H-M-S-S-M-S-S-
L-G-G-P-A-A-F-S-A-R-W-A-Q-E
794 J14 3876.6 F-Q-P-H-P-G-L-Q-K-T-L-E-Q-F-H-F-S-S-M-S-S-
L-G-G-P-A-A-F-S-A-R-W-A-Q-E
795 J15 3826.5 F-Q-P-H-P-G-L-Q-K-T-L-E-Q-F-H-P-S-S-M-S-S-
L-G-G-P-A-A-F-S-A-R-W-A-Q-E
796 J16 3816.5 F-Q-P-H-P-G-L-Q-K-T-L-E-Q-F-H-S-S-S-M-S-S-
L-G-G-P-A-A-F-S-A-R-W-A-Q-E
797 J17 3830.5 F-Q-P-H-P-G-L-Q-K-T-L-E-Q-F-H-T-S-S-M-S-S-
L-G-G-P-A-A-F-S-A-R-W-A-Q-E
798 J18 3915.6 F-Q-P-H-P-G-L-Q-K-T-L-E-Q-F-H-W-S-S-M-S-S-
L-G-G-P-A-A-F-S-A-R-W-A-Q-E
799 J19 3892.6 F-Q-P-H-P-G-L-Q-K-T-L-E-Q-F-H-Y-S-S-M-S-S-
L-G-G-P-A-A-F-S-A-R-W-A-Q-E
800 J20 3828.5 F-Q-P-H-P-G-L-Q-K-T-L-E-Q-F-H-V-S-S-M-S-S-
L-G-G-P-A-A-F-S-A-R-W-A-Q-E
801 K 1 3826.6 F-Q-P-H-P-G-L-Q-K-T-L-E-Q-F-H-L-A-S-M-S-S-
L-G-G-P-A-A-F-S-A-R-W-A-Q-E
802 K 2 3911.7 F-Q-P-H-P-G-L-Q-K-T-L-E-Q-F-H-L-R-S-M-S-S-
L-G-G-P-A-A-F-S-A-R-W-A-Q-E
803 K 3 3869.6 F-Q-P-H-P-G-L-Q-K-T-L-E-Q-F-H-L-N-S-M-S-S-
L-G-G-P-A-A-F-S-A-R-W-A-Q-E
804 K 4 3870.6 F-Q-P-H-P-G-L-Q-K-T-L-E-Q-F-H-L-D-S-M-S-S-
L-G-G-P-A-A-F-S-A-R-W-A-Q-E
805 K 5 3858.6 F-Q-P-H-P-G-L-Q-K-T-L-E-Q-F-H-L-C-S-M-S-S-
L-G-G-P-A-A-F-S-A-R-W-A-Q-E
806 K 6 3883.6 F-Q-P-H-P-G-L-Q-K-T-L-E-Q-F-H-L-Q-S-M-S-S-
L-G-G-P-A-A-F-S-A-R-W-A-Q-E
807 K 7 3884.6 F-Q-P-H-P-G-L-Q-K-T-L-E-Q-F-H-L-E-S-M-S-S-
L-G-G-P-A-A-F-S-A-R-W-A-Q-E
808 K 8 3812.6 F-Q-P-H-P-G-L-Q-K-T-L-E-Q-F-H-L-G-S-M-S-S-

L-G-G-P-A-A-F-S-A-R-W-A-Q-E
809 K 9 3892.6 F-Q-P-H-P-G-L-Q-K-T-L-E-Q-F-H-L-H-S-M-S-S-
L-G-G-P-A-A-F-S-A-R-W-A-Q-E
810 K10 3868.7 F-Q-P-H-P-G-L-Q-K-T-L-E-Q-F-H-L-I-S-M-S-S-
L-G-G-P-A-A-F-S-A-R-W-A-Q-E
811 K11 3868.7 F-Q-P-H-P-G-L-Q-K-T-L-E-Q-F-H-L-L-S-M-S-S-
L-G-G-P-A-A-F-S-A-R-W-A-Q-E
812 K12 3883.7 F-Q-P-H-P-G-L-Q-K-T-L-E-Q-F-H-L-K-S-M-S-S-
L-G-G-P-A-A-F-S-A-R-W-A-Q-E
813 K13 3886.7 F-Q-P-H-P-G-L-Q-K-T-L-E-Q-F-H-L-M-S-M-S-S-
L-G-G-P-A-A-F-S-A-R-W-A-Q-E
814 K14 3902.7 F-Q-P-H-P-G-L-Q-K-T-L-E-Q-F-H-L-F-S-M-S-S-
L-G-G-P-A-A-F-S-A-R-W-A-Q-E
815 K15 3852.6 F-Q-P-H-P-G-L-Q-K-T-L-E-Q-F-H-L-P-S-M-S-S-
L-G-G-P-A-A-F-S-A-R-W-A-Q-E
816 K16 3842.6 F-Q-P-H-P-G-L-Q-K-T-L-E-Q-F-H-L-S-S-M-S-S-
L-G-G-P-A-A-F-S-A-R-W-A-Q-E
817 K17 3856.6 F-Q-P-H-P-G-L-Q-K-T-L-E-Q-F-H-L-T-S-M-S-S-
L-G-G-P-A-A-F-S-A-R-W-A-Q-E
818 K18 3941.7 F-Q-P-H-P-G-L-Q-K-T-L-E-Q-F-H-L-W-S-M-S-S-
L-G-G-P-A-A-F-S-A-R-W-A-Q-E
819 K19 3918.7 F-Q-P-H-P-G-L-Q-K-T-L-E-Q-F-H-L-Y-S-M-S-S-
L-G-G-P-A-A-F-S-A-R-W-A-Q-E
820 K20 3854.6 F-Q-P-H-P-G-L-Q-K-T-L-E-Q-F-H-L-V-S-M-S-S-
L-G-G-P-A-A-F-S-A-R-W-A-Q-E
821 L 1 3826.6 F-Q-P-H-P-G-L-Q-K-T-L-E-Q-F-H-L-S-A-M-S-S-
L-G-G-P-A-A-F-S-A-R-W-A-Q-E
822 L 2 3911.7 F-Q-P-H-P-G-L-Q-K-T-L-E-Q-F-H-L-S-R-M-S-S-
L-G-G-P-A-A-F-S-A-R-W-A-Q-E
823 L 3 3869.6 F-Q-P-H-P-G-L-Q-K-T-L-E-Q-F-H-L-S-N-M-S-S-
L-G-G-P-A-A-F-S-A-R-W-A-Q-E
824 L 4 3870.6 F-Q-P-H-P-G-L-Q-K-T-L-E-Q-F-H-L-S-D-M-S-S-
L-G-G-P-A-A-F-S-A-R-W-A-Q-E
825 L 5 3858.6 F-Q-P-H-P-G-L-Q-K-T-L-E-Q-F-H-L-S-C-M-S-S-
L-G-G-P-A-A-F-S-A-R-W-A-Q-E
826 L 6 3883.6 F-Q-P-H-P-G-L-Q-K-T-L-E-Q-F-H-L-S-Q-M-S-S-
L-G-G-P-A-A-F-S-A-R-W-A-Q-E
827 L 7 3884.6 F-Q-P-H-P-G-L-Q-K-T-L-E-Q-F-H-L-S-E-M-S-S-
L-G-G-P-A-A-F-S-A-R-W-A-Q-E
828 L 8 3812.6 F-Q-P-H-P-G-L-Q-K-T-L-E-Q-F-H-L-S-G-M-S-S-
L-G-G-P-A-A-F-S-A-R-W-A-Q-E
829 L 9 3892.6 F-Q-P-H-P-G-L-Q-K-T-L-E-Q-F-H-L-S-H-M-S-S-
L-G-G-P-A-A-F-S-A-R-W-A-Q-E
830 L10 3868.7 F-Q-P-H-P-G-L-Q-K-T-L-E-Q-F-H-L-S-I-M-S-S-
L-G-G-P-A-A-F-S-A-R-W-A-Q-E
831 L11 3868.7 F-Q-P-H-P-G-L-Q-K-T-L-E-Q-F-H-L-S-L-M-S-S-
L-G-G-P-A-A-F-S-A-R-W-A-Q-E
832 L12 3883.7 F-Q-P-H-P-G-L-Q-K-T-L-E-Q-F-H-L-S-K-M-S-S-
L-G-G-P-A-A-F-S-A-R-W-A-Q-E
833 L13 3886.7 F-Q-P-H-P-G-L-Q-K-T-L-E-Q-F-H-L-S-M-M-S-S-
L-G-G-P-A-A-F-S-A-R-W-A-Q-E
834 L14 3902.7 F-Q-P-H-P-G-L-Q-K-T-L-E-Q-F-H-L-S-F-M-S-S-
L-G-G-P-A-A-F-S-A-R-W-A-Q-E
835 L15 3852.6 F-Q-P-H-P-G-L-Q-K-T-L-E-Q-F-H-L-S-P-M-S-S-

L-G-G-P-A-A-F-S-A-R-W-A-Q-E
836 L16 3842.6 F-Q-P-H-P-G-L-Q-K-T-L-E-Q-F-H-L-S-S-M-S-S-
L-G-G-P-A-A-F-S-A-R-W-A-Q-E
837 L17 3856.6 F-Q-P-H-P-G-L-Q-K-T-L-E-Q-F-H-L-S-T-M-S-S-
L-G-G-P-A-A-F-S-A-R-W-A-Q-E
838 L18 3941.7 F-Q-P-H-P-G-L-Q-K-T-L-E-Q-F-H-L-S-W-M-S-S-
L-G-G-P-A-A-F-S-A-R-W-A-Q-E
839 L19 3918.7 F-Q-P-H-P-G-L-Q-K-T-L-E-Q-F-H-L-S-Y-M-S-S-
L-G-G-P-A-A-F-S-A-R-W-A-Q-E
840 L20 3854.6 F-Q-P-H-P-G-L-Q-K-T-L-E-Q-F-H-L-S-V-M-S-S-
L-G-G-P-A-A-F-S-A-R-W-A-Q-E
841 M 1 3782.5 F-Q-P-H-P-G-L-Q-K-T-L-E-Q-F-H-L-S-S-A-S-S-
L-G-G-P-A-A-F-S-A-R-W-A-Q-E
842 M 2 3867.6 F-Q-P-H-P-G-L-Q-K-T-L-E-Q-F-H-L-S-S-R-S-S-
L-G-G-P-A-A-F-S-A-R-W-A-Q-E
843 M 3 3825.5 F-Q-P-H-P-G-L-Q-K-T-L-E-Q-F-H-L-S-S-N-S-S-
L-G-G-P-A-A-F-S-A-R-W-A-Q-E
844 M 4 3826.5 F-Q-P-H-P-G-L-Q-K-T-L-E-Q-F-H-L-S-S-D-S-S-
L-G-G-P-A-A-F-S-A-R-W-A-Q-E
845 M 5 3814.5 F-Q-P-H-P-G-L-Q-K-T-L-E-Q-F-H-L-S-S-C-S-S-
L-G-G-P-A-A-F-S-A-R-W-A-Q-E
846 M 6 3839.5 F-Q-P-H-P-G-L-Q-K-T-L-E-Q-F-H-L-S-S-Q-S-S-
L-G-G-P-A-A-F-S-A-R-W-A-Q-E
847 M 7 3840.5 F-Q-P-H-P-G-L-Q-K-T-L-E-Q-F-H-L-S-S-E-S-S-
L-G-G-P-A-A-F-S-A-R-W-A-Q-E
848 M 8 3768.5 F-Q-P-H-P-G-L-Q-K-T-L-E-Q-F-H-L-S-S-G-S-S-
L-G-G-P-A-A-F-S-A-R-W-A-Q-E
849 M 9 3848.5 F-Q-P-H-P-G-L-Q-K-T-L-E-Q-F-H-L-S-S-H-S-S-
L-G-G-P-A-A-F-S-A-R-W-A-Q-E
850 M10 3824.6 F-Q-P-H-P-G-L-Q-K-T-L-E-Q-F-H-L-S-S-I-S-S-
L-G-G-P-A-A-F-S-A-R-W-A-Q-E
851 M11 3824.6 F-Q-P-H-P-G-L-Q-K-T-L-E-Q-F-H-L-S-S-L-S-S-
L-G-G-P-A-A-F-S-A-R-W-A-Q-E
852 M12 3839.6 F-Q-P-H-P-G-L-Q-K-T-L-E-Q-F-H-L-S-S-K-S-S-
L-G-G-P-A-A-F-S-A-R-W-A-Q-E
853 M13 3842.6 F-Q-P-H-P-G-L-Q-K-T-L-E-Q-F-H-L-S-S-M-S-S-
L-G-G-P-A-A-F-S-A-R-W-A-Q-E
854 M14 3858.6 F-Q-P-H-P-G-L-Q-K-T-L-E-Q-F-H-L-S-S-F-S-S-
L-G-G-P-A-A-F-S-A-R-W-A-Q-E
855 M15 3808.5 F-Q-P-H-P-G-L-Q-K-T-L-E-Q-F-H-L-S-S-P-S-S-
L-G-G-P-A-A-F-S-A-R-W-A-Q-E
856 M16 3798.5 F-Q-P-H-P-G-L-Q-K-T-L-E-Q-F-H-L-S-S-S-S-S-
L-G-G-P-A-A-F-S-A-R-W-A-Q-E
857 M17 3812.5 F-Q-P-H-P-G-L-Q-K-T-L-E-Q-F-H-L-S-S-T-S-S-
L-G-G-P-A-A-F-S-A-R-W-A-Q-E
858 M18 3897.6 F-Q-P-H-P-G-L-Q-K-T-L-E-Q-F-H-L-S-S-W-S-S-
L-G-G-P-A-A-F-S-A-R-W-A-Q-E
859 M19 3874.6 F-Q-P-H-P-G-L-Q-K-T-L-E-Q-F-H-L-S-S-Y-S-S-
L-G-G-P-A-A-F-S-A-R-W-A-Q-E
860 M20 3810.5 F-Q-P-H-P-G-L-Q-K-T-L-E-Q-F-H-L-S-S-V-S-S-
L-G-G-P-A-A-F-S-A-R-W-A-Q-E
861 N 1 3826.6 F-Q-P-H-P-G-L-Q-K-T-L-E-Q-F-H-L-S-S-M-A-S-
L-G-G-P-A-A-F-S-A-R-W-A-Q-E
862 N 2 3911.7 F-Q-P-H-P-G-L-Q-K-T-L-E-Q-F-H-L-S-S-M-R-S-

L-G-G-P-A-A-F-S-A-R-W-A-Q-E
863 N 3 3869.6 F-Q-P-H-P-G-L-Q-K-T-L-E-Q-F-H-L-S-S-M-N-S-
L-G-G-P-A-A-F-S-A-R-W-A-Q-E
864 N 4 3870.6 F-Q-P-H-P-G-L-Q-K-T-L-E-Q-F-H-L-S-S-M-D-S-
L-G-G-P-A-A-F-S-A-R-W-A-Q-E
865 N 5 3858.6 F-Q-P-H-P-G-L-Q-K-T-L-E-Q-F-H-L-S-S-M-C-S-
L-G-G-P-A-A-F-S-A-R-W-A-Q-E
866 N 6 3883.6 F-Q-P-H-P-G-L-Q-K-T-L-E-Q-F-H-L-S-S-M-Q-S-
L-G-G-P-A-A-F-S-A-R-W-A-Q-E
867 N 7 3884.6 F-Q-P-H-P-G-L-Q-K-T-L-E-Q-F-H-L-S-S-M-E-S-
L-G-G-P-A-A-F-S-A-R-W-A-Q-E
868 N 8 3812.6 F-Q-P-H-P-G-L-Q-K-T-L-E-Q-F-H-L-S-S-M-G-S-
L-G-G-P-A-A-F-S-A-R-W-A-Q-E
869 N 9 3892.6 F-Q-P-H-P-G-L-Q-K-T-L-E-Q-F-H-L-S-S-M-H-S-
L-G-G-P-A-A-F-S-A-R-W-A-Q-E
870 N10 3868.7 F-Q-P-H-P-G-L-Q-K-T-L-E-Q-F-H-L-S-S-M-I-S-
L-G-G-P-A-A-F-S-A-R-W-A-Q-E
871 N11 3868.7 F-Q-P-H-P-G-L-Q-K-T-L-E-Q-F-H-L-S-S-M-L-S-
L-G-G-P-A-A-F-S-A-R-W-A-Q-E
872 N12 3883.7 F-Q-P-H-P-G-L-Q-K-T-L-E-Q-F-H-L-S-S-M-K-S-
L-G-G-P-A-A-F-S-A-R-W-A-Q-E
873 N13 3886.7 F-Q-P-H-P-G-L-Q-K-T-L-E-Q-F-H-L-S-S-M-M-S-
L-G-G-P-A-A-F-S-A-R-W-A-Q-E
874 N14 3902.7 F-Q-P-H-P-G-L-Q-K-T-L-E-Q-F-H-L-S-S-M-F-S-
L-G-G-P-A-A-F-S-A-R-W-A-Q-E
875 N15 3852.6 F-Q-P-H-P-G-L-Q-K-T-L-E-Q-F-H-L-S-S-M-P-S-
L-G-G-P-A-A-F-S-A-R-W-A-Q-E
876 N16 3842.6 F-Q-P-H-P-G-L-Q-K-T-L-E-Q-F-H-L-S-S-M-S-S-
L-G-G-P-A-A-F-S-A-R-W-A-Q-E
877 N17 3856.6 F-Q-P-H-P-G-L-Q-K-T-L-E-Q-F-H-L-S-S-M-T-S-
L-G-G-P-A-A-F-S-A-R-W-A-Q-E
878 N18 3941.7 F-Q-P-H-P-G-L-Q-K-T-L-E-Q-F-H-L-S-S-M-W-S-
L-G-G-P-A-A-F-S-A-R-W-A-Q-E
879 N19 3918.7 F-Q-P-H-P-G-L-Q-K-T-L-E-Q-F-H-L-S-S-M-Y-S-
L-G-G-P-A-A-F-S-A-R-W-A-Q-E
880 N20 3854.6 F-Q-P-H-P-G-L-Q-K-T-L-E-Q-F-H-L-S-S-M-V-S-
L-G-G-P-A-A-F-S-A-R-W-A-Q-E
881 O 1 3826.6 F-Q-P-H-P-G-L-Q-K-T-L-E-Q-F-H-L-S-S-M-S-A-
L-G-G-P-A-A-F-S-A-R-W-A-Q-E
882 O 2 3911.7 F-Q-P-H-P-G-L-Q-K-T-L-E-Q-F-H-L-S-S-M-S-R-
L-G-G-P-A-A-F-S-A-R-W-A-Q-E
883 O 3 3869.6 F-Q-P-H-P-G-L-Q-K-T-L-E-Q-F-H-L-S-S-M-S-N-
L-G-G-P-A-A-F-S-A-R-W-A-Q-E
884 O 4 3870.6 F-Q-P-H-P-G-L-Q-K-T-L-E-Q-F-H-L-S-S-M-S-D-
L-G-G-P-A-A-F-S-A-R-W-A-Q-E
885 O 5 3858.6 F-Q-P-H-P-G-L-Q-K-T-L-E-Q-F-H-L-S-S-M-S-C-
L-G-G-P-A-A-F-S-A-R-W-A-Q-E
886 O 6 3883.6 F-Q-P-H-P-G-L-Q-K-T-L-E-Q-F-H-L-S-S-M-S-Q-
L-G-G-P-A-A-F-S-A-R-W-A-Q-E
887 O 7 3884.6 F-Q-P-H-P-G-L-Q-K-T-L-E-Q-F-H-L-S-S-M-S-E-
L-G-G-P-A-A-F-S-A-R-W-A-Q-E
888 O 8 3812.6 F-Q-P-H-P-G-L-Q-K-T-L-E-Q-F-H-L-S-S-M-S-G-
L-G-G-P-A-A-F-S-A-R-W-A-Q-E
889 O 9 3892.6 F-Q-P-H-P-G-L-Q-K-T-L-E-Q-F-H-L-S-S-M-S-H-

L-G-G-P-A-A-F-S-A-R-W-A-Q-E
890 O10 3868.7 F-Q-P-H-P-G-L-Q-K-T-L-E-Q-F-H-L-S-S-M-S-I-
L-G-G-P-A-A-F-S-A-R-W-A-Q-E
891 O11 3868.7 F-Q-P-H-P-G-L-Q-K-T-L-E-Q-F-H-L-S-S-M-S-L-
L-G-G-P-A-A-F-S-A-R-W-A-Q-E
892 O12 3883.7 F-Q-P-H-P-G-L-Q-K-T-L-E-Q-F-H-L-S-S-M-S-K-
L-G-G-P-A-A-F-S-A-R-W-A-Q-E
893 O13 3886.7 F-Q-P-H-P-G-L-Q-K-T-L-E-Q-F-H-L-S-S-M-S-M-
L-G-G-P-A-A-F-S-A-R-W-A-Q-E
894 O14 3902.7 F-Q-P-H-P-G-L-Q-K-T-L-E-Q-F-H-L-S-S-M-S-F-
L-G-G-P-A-A-F-S-A-R-W-A-Q-E
895 O15 3852.6 F-Q-P-H-P-G-L-Q-K-T-L-E-Q-F-H-L-S-S-M-S-P-
L-G-G-P-A-A-F-S-A-R-W-A-Q-E
896 O16 3842.6 F-Q-P-H-P-G-L-Q-K-T-L-E-Q-F-H-L-S-S-M-S-S-
L-G-G-P-A-A-F-S-A-R-W-A-Q-E
897 O17 3856.6 F-Q-P-H-P-G-L-Q-K-T-L-E-Q-F-H-L-S-S-M-S-T-
L-G-G-P-A-A-F-S-A-R-W-A-Q-E
898 O18 3941.7 F-Q-P-H-P-G-L-Q-K-T-L-E-Q-F-H-L-S-S-M-S-W-
L-G-G-P-A-A-F-S-A-R-W-A-Q-E
899 O19 3918.7 F-Q-P-H-P-G-L-Q-K-T-L-E-Q-F-H-L-S-S-M-S-Y-
L-G-G-P-A-A-F-S-A-R-W-A-Q-E
900 O20 3854.6 F-Q-P-H-P-G-L-Q-K-T-L-E-Q-F-H-L-S-S-M-S-V-
L-G-G-P-A-A-F-S-A-R-W-A-Q-E
901 P 1 3800.5 F-Q-P-H-P-G-L-Q-K-T-L-E-Q-F-H-L-S-S-M-S-S-
A-G-G-P-A-A-F-S-A-R-W-A-Q-E
902 P 2 3885.6 F-Q-P-H-P-G-L-Q-K-T-L-E-Q-F-H-L-S-S-M-S-S-
R-G-G-P-A-A-F-S-A-R-W-A-Q-E
903 P 3 3843.5 F-Q-P-H-P-G-L-Q-K-T-L-E-Q-F-H-L-S-S-M-S-S-
N-G-G-P-A-A-F-S-A-R-W-A-Q-E
904 P 4 3844.5 F-Q-P-H-P-G-L-Q-K-T-L-E-Q-F-H-L-S-S-M-S-S-
D-G-G-P-A-A-F-S-A-R-W-A-Q-E
905 P 5 3832.5 F-Q-P-H-P-G-L-Q-K-T-L-E-Q-F-H-L-S-S-M-S-S-
C-G-G-P-A-A-F-S-A-R-W-A-Q-E
906 P 6 3857.5 F-Q-P-H-P-G-L-Q-K-T-L-E-Q-F-H-L-S-S-M-S-S-
Q-G-G-P-A-A-F-S-A-R-W-A-Q-E
907 P 7 3858.5 F-Q-P-H-P-G-L-Q-K-T-L-E-Q-F-H-L-S-S-M-S-S-
E-G-G-P-A-A-F-S-A-R-W-A-Q-E
908 P 8 3786.5 F-Q-P-H-P-G-L-Q-K-T-L-E-Q-F-H-L-S-S-M-S-S-
G-G-G-P-A-A-F-S-A-R-W-A-Q-E
909 P 9 3866.5 F-Q-P-H-P-G-L-Q-K-T-L-E-Q-F-H-L-S-S-M-S-S-
H-G-G-P-A-A-F-S-A-R-W-A-Q-E
910 P10 3842.6 F-Q-P-H-P-G-L-Q-K-T-L-E-Q-F-H-L-S-S-M-S-S-
I-G-G-P-A-A-F-S-A-R-W-A-Q-E
911 P11 3842.6 F-Q-P-H-P-G-L-Q-K-T-L-E-Q-F-H-L-S-S-M-S-S-
L-G-G-P-A-A-F-S-A-R-W-A-Q-E
912 P12 3857.6 F-Q-P-H-P-G-L-Q-K-T-L-E-Q-F-H-L-S-S-M-S-S-
K-G-G-P-A-A-F-S-A-R-W-A-Q-E
913 P13 3860.6 F-Q-P-H-P-G-L-Q-K-T-L-E-Q-F-H-L-S-S-M-S-S-
M-G-G-P-A-A-F-S-A-R-W-A-Q-E
914 P14 3876.6 F-Q-P-H-P-G-L-Q-K-T-L-E-Q-F-H-L-S-S-M-S-S-
F-G-G-P-A-A-F-S-A-R-W-A-Q-E
915 P15 3826.5 F-Q-P-H-P-G-L-Q-K-T-L-E-Q-F-H-L-S-S-M-S-S-
P-G-G-P-A-A-F-S-A-R-W-A-Q-E
916 P16 3816.5 F-Q-P-H-P-G-L-Q-K-T-L-E-Q-F-H-L-S-S-M-S-S-

S-G-G-P-A-A-F-S-A-R-W-A-Q-E
917 P17 3830.5 F-Q-P-H-P-G-L-Q-K-T-L-E-Q-F-H-L-S-S-M-S-S-T-G-G-P-A-A-F-S-A-R-W-A-Q-E
918 P18 3915.6 F-Q-P-H-P-G-L-Q-K-T-L-E-Q-F-H-L-S-S-M-S-S-W-G-G-P-A-A-F-S-A-R-W-A-Q-E
919 P19 3892.6 F-Q-P-H-P-G-L-Q-K-T-L-E-Q-F-H-L-S-S-M-S-S-Y-G-G-P-A-A-F-S-A-R-W-A-Q-E
920 P20 3828.5 F-Q-P-H-P-G-L-Q-K-T-L-E-Q-F-H-L-S-S-M-S-S-V-G-G-P-A-A-F-S-A-R-W-A-Q-E
921 Q 1 3856.6 F-Q-P-H-P-G-L-Q-K-T-L-E-Q-F-H-L-S-S-M-S-S-L-A-G-P-A-A-F-S-A-R-W-A-Q-E
922 Q 2 3941.7 F-Q-P-H-P-G-L-Q-K-T-L-E-Q-F-H-L-S-S-M-S-S-L-R-G-P-A-A-F-S-A-R-W-A-Q-E
923 Q 3 3899.6 F-Q-P-H-P-G-L-Q-K-T-L-E-Q-F-H-L-S-S-M-S-S-L-N-G-P-A-A-F-S-A-R-W-A-Q-E
924 Q 4 3900.6 F-Q-P-H-P-G-L-Q-K-T-L-E-Q-F-H-L-S-S-M-S-S-L-D-G-P-A-A-F-S-A-R-W-A-Q-E
925 Q 5 3888.6 F-Q-P-H-P-G-L-Q-K-T-L-E-Q-F-H-L-S-S-M-S-S-L-C-G-P-A-A-F-S-A-R-W-A-Q-E
926 Q 6 3913.6 F-Q-P-H-P-G-L-Q-K-T-L-E-Q-F-H-L-S-S-M-S-S-L-Q-G-P-A-A-F-S-A-R-W-A-Q-E
927 Q 7 3914.6 F-Q-P-H-P-G-L-Q-K-T-L-E-Q-F-H-L-S-S-M-S-S-L-E-G-P-A-A-F-S-A-R-W-A-Q-E
928 Q 8 3842.6 F-Q-P-H-P-G-L-Q-K-T-L-E-Q-F-H-L-S-S-M-S-S-L-G-G-P-A-A-F-S-A-R-W-A-Q-E
929 Q 9 3922.6 F-Q-P-H-P-G-L-Q-K-T-L-E-Q-F-H-L-S-S-M-S-S-L-H-G-P-A-A-F-S-A-R-W-A-Q-E
930 Q10 3898.7 F-Q-P-H-P-G-L-Q-K-T-L-E-Q-F-H-L-S-S-M-S-S-L-I-G-P-A-A-F-S-A-R-W-A-Q-E
931 Q11 3898.7 F-Q-P-H-P-G-L-Q-K-T-L-E-Q-F-H-L-S-S-M-S-S-L-L-G-P-A-A-F-S-A-R-W-A-Q-E
932 Q12 3913.7 F-Q-P-H-P-G-L-Q-K-T-L-E-Q-F-H-L-S-S-M-S-S-L-K-G-P-A-A-F-S-A-R-W-A-Q-E
933 Q13 3916.7 F-Q-P-H-P-G-L-Q-K-T-L-E-Q-F-H-L-S-S-M-S-S-L-M-G-P-A-A-F-S-A-R-W-A-Q-E
934 Q14 3932.7 F-Q-P-H-P-G-L-Q-K-T-L-E-Q-F-H-L-S-S-M-S-S-L-F-G-P-A-A-F-S-A-R-W-A-Q-E
935 Q15 3882.6 F-Q-P-H-P-G-L-Q-K-T-L-E-Q-F-H-L-S-S-M-S-S-L-P-G-P-A-A-F-S-A-R-W-A-Q-E
936 Q16 3872.6 F-Q-P-H-P-G-L-Q-K-T-L-E-Q-F-H-L-S-S-M-S-S-L-S-G-P-A-A-F-S-A-R-W-A-Q-E
937 Q17 3886.6 F-Q-P-H-P-G-L-Q-K-T-L-E-Q-F-H-L-S-S-M-S-S-L-T-G-P-A-A-F-S-A-R-W-A-Q-E
938 Q18 3971.7 F-Q-P-H-P-G-L-Q-K-T-L-E-Q-F-H-L-S-S-M-S-S-L-W-G-P-A-A-F-S-A-R-W-A-Q-E
939 Q19 3948.7 F-Q-P-H-P-G-L-Q-K-T-L-E-Q-F-H-L-S-S-M-S-S-L-Y-G-P-A-A-F-S-A-R-W-A-Q-E
940 Q20 3884.6 F-Q-P-H-P-G-L-Q-K-T-L-E-Q-F-H-L-S-S-M-S-S-L-V-G-P-A-A-F-S-A-R-W-A-Q-E
941 R 1 3856.6 F-Q-P-H-P-G-L-Q-K-T-L-E-Q-F-H-L-S-S-M-S-S-L-G-A-P-A-A-F-S-A-R-W-A-Q-E
942 R 2 3941.7 F-Q-P-H-P-G-L-Q-K-T-L-E-Q-F-H-L-S-S-M-S-S-L-G-R-P-A-A-F-S-A-R-W-A-Q-E
943 R 3 3899.6 F-Q-P-H-P-G-L-Q-K-T-L-E-Q-F-H-L-S-S-M-S-S-

L-G-N-P-A-A-F-S-A-R-W-A-Q-E
944 R 4 3900.6 F-Q-P-H-P-G-L-Q-K-T-L-E-Q-F-H-L-S-S-M-S-S-
L-G-D-P-A-A-F-S-A-R-W-A-Q-E
945 R 5 3888.6 F-Q-P-H-P-G-L-Q-K-T-L-E-Q-F-H-L-S-S-M-S-S-
L-G-C-P-A-A-F-S-A-R-W-A-Q-E
946 R 6 3913.6 F-Q-P-H-P-G-L-Q-K-T-L-E-Q-F-H-L-S-S-M-S-S-
L-G-Q-P-A-A-F-S-A-R-W-A-Q-E
947 R 7 3914.6 F-Q-P-H-P-G-L-Q-K-T-L-E-Q-F-H-L-S-S-M-S-S-
L-G-E-P-A-A-F-S-A-R-W-A-Q-E
948 R 8 3842.6 F-Q-P-H-P-G-L-Q-K-T-L-E-Q-F-H-L-S-S-M-S-S-
L-G-G-P-A-A-F-S-A-R-W-A-Q-E
949 R 9 3922.6 F-Q-P-H-P-G-L-Q-K-T-L-E-Q-F-H-L-S-S-M-S-S-
L-G-H-P-A-A-F-S-A-R-W-A-Q-E
950 R10 3898.7 F-Q-P-H-P-G-L-Q-K-T-L-E-Q-F-H-L-S-S-M-S-S-
L-G-I-P-A-A-F-S-A-R-W-A-Q-E
951 R11 3898.7 F-Q-P-H-P-G-L-Q-K-T-L-E-Q-F-H-L-S-S-M-S-S-
L-G-L-P-A-A-F-S-A-R-W-A-Q-E
952 R12 3913.7 F-Q-P-H-P-G-L-Q-K-T-L-E-Q-F-H-L-S-S-M-S-S-
L-G-K-P-A-A-F-S-A-R-W-A-Q-E
953 R13 3916.7 F-Q-P-H-P-G-L-Q-K-T-L-E-Q-F-H-L-S-S-M-S-S-
L-G-M-P-A-A-F-S-A-R-W-A-Q-E
954 R14 3932.7 F-Q-P-H-P-G-L-Q-K-T-L-E-Q-F-H-L-S-S-M-S-S-
L-G-F-P-A-A-F-S-A-R-W-A-Q-E
955 R15 3882.6 F-Q-P-H-P-G-L-Q-K-T-L-E-Q-F-H-L-S-S-M-S-S-
L-G-P-P-A-A-F-S-A-R-W-A-Q-E
956 R16 3872.6 F-Q-P-H-P-G-L-Q-K-T-L-E-Q-F-H-L-S-S-M-S-S-
L-G-S-P-A-A-F-S-A-R-W-A-Q-E
957 R17 3886.6 F-Q-P-H-P-G-L-Q-K-T-L-E-Q-F-H-L-S-S-M-S-S-
L-G-T-P-A-A-F-S-A-R-W-A-Q-E
958 R18 3971.7 F-Q-P-H-P-G-L-Q-K-T-L-E-Q-F-H-L-S-S-M-S-S-
L-G-W-P-A-A-F-S-A-R-W-A-Q-E
959 R19 3948.7 F-Q-P-H-P-G-L-Q-K-T-L-E-Q-F-H-L-S-S-M-S-S-
L-G-Y-P-A-A-F-S-A-R-W-A-Q-E
960 R20 3884.6 F-Q-P-H-P-G-L-Q-K-T-L-E-Q-F-H-L-S-S-M-S-S-
L-G-V-P-A-A-F-S-A-R-W-A-Q-E
961 S 1 3816.6 F-Q-P-H-P-G-L-Q-K-T-L-E-Q-F-H-L-S-S-M-S-S-
L-G-G-A-A-A-F-S-A-R-W-A-Q-E
962 S 2 3901.7 F-Q-P-H-P-G-L-Q-K-T-L-E-Q-F-H-L-S-S-M-S-S-
L-G-G-R-A-A-F-S-A-R-W-A-Q-E
963 S 3 3859.6 F-Q-P-H-P-G-L-Q-K-T-L-E-Q-F-H-L-S-S-M-S-S-
L-G-G-N-A-A-F-S-A-R-W-A-Q-E
964 S 4 3860.6 F-Q-P-H-P-G-L-Q-K-T-L-E-Q-F-H-L-S-S-M-S-S-
L-G-G-D-A-A-F-S-A-R-W-A-Q-E
965 S 5 3848.6 F-Q-P-H-P-G-L-Q-K-T-L-E-Q-F-H-L-S-S-M-S-S-
L-G-G-C-A-A-F-S-A-R-W-A-Q-E
966 S 6 3873.6 F-Q-P-H-P-G-L-Q-K-T-L-E-Q-F-H-L-S-S-M-S-S-
L-G-G-Q-A-A-F-S-A-R-W-A-Q-E
967 S 7 3874.6 F-Q-P-H-P-G-L-Q-K-T-L-E-Q-F-H-L-S-S-M-S-S-
L-G-G-E-A-A-F-S-A-R-W-A-Q-E
968 S 8 3802.6 F-Q-P-H-P-G-L-Q-K-T-L-E-Q-F-H-L-S-S-M-S-S-
L-G-G-G-A-A-F-S-A-R-W-A-Q-E
969 S 9 3882.6 F-Q-P-H-P-G-L-Q-K-T-L-E-Q-F-H-L-S-S-M-S-S-
L-G-G-H-A-A-F-S-A-R-W-A-Q-E
970 S10 3858.7 F-Q-P-H-P-G-L-Q-K-T-L-E-Q-F-H-L-S-S-M-S-S-

L-G-G-I-A-A-F-S-A-R-W-A-Q-E
971 S11 3858.7 F-Q-P-H-P-G-L-Q-K-T-L-E-Q-F-H-L-S-S-M-S-S-
L-G-G-L-A-A-F-S-A-R-W-A-Q-E
972 S12 3873.7 F-Q-P-H-P-G-L-Q-K-T-L-E-Q-F-H-L-S-S-M-S-S-
L-G-G-K-A-A-F-S-A-R-W-A-Q-E
973 S13 3876.7 F-Q-P-H-P-G-L-Q-K-T-L-E-Q-F-H-L-S-S-M-S-S-
L-G-G-M-A-A-F-S-A-R-W-A-Q-E
974 S14 3892.7 F-Q-P-H-P-G-L-Q-K-T-L-E-Q-F-H-L-S-S-M-S-S-
L-G-G-F-A-A-F-S-A-R-W-A-Q-E
975 S15 3842.6 F-Q-P-H-P-G-L-Q-K-T-L-E-Q-F-H-L-S-S-M-S-S-
L-G-G-P-A-A-F-S-A-R-W-A-Q-E
976 S16 3832.6 F-Q-P-H-P-G-L-Q-K-T-L-E-Q-F-H-L-S-S-M-S-S-
L-G-G-S-A-A-F-S-A-R-W-A-Q-E
977 S17 3846.6 F-Q-P-H-P-G-L-Q-K-T-L-E-Q-F-H-L-S-S-M-S-S-
L-G-G-T-A-A-F-S-A-R-W-A-Q-E
978 S18 3931.7 F-Q-P-H-P-G-L-Q-K-T-L-E-Q-F-H-L-S-S-M-S-S-
L-G-G-W-A-A-F-S-A-R-W-A-Q-E
979 S19 3908.7 F-Q-P-H-P-G-L-Q-K-T-L-E-Q-F-H-L-S-S-M-S-S-
L-G-G-Y-A-A-F-S-A-R-W-A-Q-E
980 S20 3844.6 F-Q-P-H-P-G-L-Q-K-T-L-E-Q-F-H-L-S-S-M-S-S-
L-G-G-V-A-A-F-S-A-R-W-A-Q-E
981 T 1 0

982 T 2 0

983 T 3 0

984 T 4 0

985 T 5 0

986 T 6 0

987 T 7 0

988 T 8 0

989 T 9 0

990 T10 0

991 T11 0

992 T12 0

993 T13 0

994 T14 0

995 T15 0

996 T16 0

997 T17 0

998 T18 0

999 T19 0

1000 T20 0

1001 U 1 0

1002 U 2 0

1003 U 3 0

1004 U 4 0

1005 U 5 0

1006 U 6 0

1007 U 7 0

1008 U 8 0

1009 U 9 0

1010 U10 0

1011 U11 0

1012 U12 0

1013 U13 0

1014 U14 0

1015 U15 0

1016 U16 0

1017 U17 0

1018 U18 0

1019 U19 0

1020 U20 0

1021 V 1 3842.6 F-Q-P-H-P-G-L-Q-K-T-L-E-Q-F-H-L-S-S-M-S-S-
L-G-G-P-A-A-F-S-A-R-W-A-Q-E
1022 V 2 3842.6 F-Q-P-H-P-G-L-Q-K-T-L-E-Q-F-H-L-S-S-M-S-S-
L-G-G-P-A-A-F-S-A-R-W-A-Q-E
1023 V 3 3842.6 F-Q-P-H-P-G-L-Q-K-T-L-E-Q-F-H-L-S-S-M-S-S-
L-G-G-P-A-A-F-S-A-R-W-A-Q-E
1024 V 4 3842.6 F-Q-P-H-P-G-L-Q-K-T-L-E-Q-F-H-L-S-S-M-S-S-

L-G-G-P-A-A-F-S-A-R-W-A-Q-E
1025 V 5 3842.6 F-Q-P-H-P-G-L-Q-K-T-L-E-Q-F-H-L-S-S-M-S-S-
L-G-G-P-A-A-F-S-A-R-W-A-Q-E
1026 V 6 0

1027 V 7 0

1028 V 8 3842.6 F-Q-P-H-P-G-L-Q-K-T-L-E-Q-F-H-L-S-S-M-S-S-
L-G-G-P-A-A-F-S-A-R-W-A-Q-E
1029 V 9 3842.6 F-Q-P-H-P-G-L-Q-K-T-L-E-Q-F-H-L-S-S-M-S-S-
L-G-G-P-A-A-F-S-A-R-W-A-Q-E
1030 V10 3842.6 F-Q-P-H-P-G-L-Q-K-T-L-E-Q-F-H-L-S-S-M-S-S-
L-G-G-P-A-A-F-S-A-R-W-A-Q-E
1031 V11 3842.6 F-Q-P-H-P-G-L-Q-K-T-L-E-Q-F-H-L-S-S-M-S-S-
L-G-G-P-A-A-F-S-A-R-W-A-Q-E
1032 V12 3842.6 F-Q-P-H-P-G-L-Q-K-T-L-E-Q-F-H-L-S-S-M-S-S-
L-G-G-P-A-A-F-S-A-R-W-A-Q-E
1033 V13 0

1034 V14 0

1035 V15 3842.6 F-Q-P-H-P-G-L-Q-K-T-L-E-Q-F-H-L-S-S-M-S-S-
L-G-G-P-A-A-F-S-A-R-W-A-Q-E
1036 V16 3842.6 F-Q-P-H-P-G-L-Q-K-T-L-E-Q-F-H-L-S-S-M-S-S-
L-G-G-P-A-A-F-S-A-R-W-A-Q-E
1037 V17 3842.6 F-Q-P-H-P-G-L-Q-K-T-L-E-Q-F-H-L-S-S-M-S-S-
L-G-G-P-A-A-F-S-A-R-W-A-Q-E
1038 V18 3842.6 F-Q-P-H-P-G-L-Q-K-T-L-E-Q-F-H-L-S-S-M-S-S-
L-G-G-P-A-A-F-S-A-R-W-A-Q-E
1039 V19 3842.6 F-Q-P-H-P-G-L-Q-K-T-L-E-Q-F-H-L-S-S-M-S-S-
L-G-G-P-A-A-F-S-A-R-W-A-Q-E
1040 V20 0

1041 W 1 3842.6 F-Q-P-H-P-G-L-Q-K-T-L-E-Q-F-H-L-S-S-M-S-S-
L-G-G-P-A-A-F-S-A-R-W-A-Q-E
1042 W 2 3842.6 F-Q-P-H-P-G-L-Q-K-T-L-E-Q-F-H-L-S-S-M-S-S-
L-G-G-P-A-A-F-S-A-R-W-A-Q-E
1043 W 3 3842.6 F-Q-P-H-P-G-L-Q-K-T-L-E-Q-F-H-L-S-S-M-S-S-
L-G-G-P-A-A-F-S-A-R-W-A-Q-E
1044 W 4 3842.6 F-Q-P-H-P-G-L-Q-K-T-L-E-Q-F-H-L-S-S-M-S-S-
L-G-G-P-A-A-F-S-A-R-W-A-Q-E
1045 W 5 3842.6 F-Q-P-H-P-G-L-Q-K-T-L-E-Q-F-H-L-S-S-M-S-S-
L-G-G-P-A-A-F-S-A-R-W-A-Q-E
1046 W 6 0

1047 W 7 0

1048 W 8 3842.6 F-Q-P-H-P-G-L-Q-K-T-L-E-Q-F-H-L-S-S-M-S-S-
L-G-G-P-A-A-F-S-A-R-W-A-Q-E
1049 W 9 3842.6 F-Q-P-H-P-G-L-Q-K-T-L-E-Q-F-H-L-S-S-M-S-S-
L-G-G-P-A-A-F-S-A-R-W-A-Q-E
1050 W10 3842.6 F-Q-P-H-P-G-L-Q-K-T-L-E-Q-F-H-L-S-S-M-S-S-
L-G-G-P-A-A-F-S-A-R-W-A-Q-E
1051 W11 3842.6 F-Q-P-H-P-G-L-Q-K-T-L-E-Q-F-H-L-S-S-M-S-S-

L-G-G-P-A-A-F-S-A-R-W-A-Q-E
1052 W12 3842.6 F-Q-P-H-P-G-L-Q-K-T-L-E-Q-F-H-L-S-S-M-S-S-
L-G-G-P-A-A-F-S-A-R-W-A-Q-E
1053 W13 0

1054 W14 0

1055 W15 3842.6 F-Q-P-H-P-G-L-Q-K-T-L-E-Q-F-H-L-S-S-M-S-S-
L-G-G-P-A-A-F-S-A-R-W-A-Q-E
1056 W16 3842.6 F-Q-P-H-P-G-L-Q-K-T-L-E-Q-F-H-L-S-S-M-S-S-
L-G-G-P-A-A-F-S-A-R-W-A-Q-E
1057 W17 3842.6 F-Q-P-H-P-G-L-Q-K-T-L-E-Q-F-H-L-S-S-M-S-S-
L-G-G-P-A-A-F-S-A-R-W-A-Q-E
1058 W18 3842.6 F-Q-P-H-P-G-L-Q-K-T-L-E-Q-F-H-L-S-S-M-S-S-
L-G-G-P-A-A-F-S-A-R-W-A-Q-E
1059 W19 3842.6 F-Q-P-H-P-G-L-Q-K-T-L-E-Q-F-H-L-S-S-M-S-S-
L-G-G-P-A-A-F-S-A-R-W-A-Q-E
1060 W20 0

1061 X 1 3842.6 F-Q-P-H-P-G-L-Q-K-T-L-E-Q-F-H-L-S-S-M-S-S-
L-G-G-P-A-A-F-S-A-R-W-A-Q-E
1062 X 2 3842.6 F-Q-P-H-P-G-L-Q-K-T-L-E-Q-F-H-L-S-S-M-S-S-
L-G-G-P-A-A-F-S-A-R-W-A-Q-E
1063 X 3 3842.6 F-Q-P-H-P-G-L-Q-K-T-L-E-Q-F-H-L-S-S-M-S-S-
L-G-G-P-A-A-F-S-A-R-W-A-Q-E
1064 X 4 3842.6 F-Q-P-H-P-G-L-Q-K-T-L-E-Q-F-H-L-S-S-M-S-S-
L-G-G-P-A-A-F-S-A-R-W-A-Q-E
1065 X 5 3842.6 F-Q-P-H-P-G-L-Q-K-T-L-E-Q-F-H-L-S-S-M-S-S-
L-G-G-P-A-A-F-S-A-R-W-A-Q-E
1066 X 6 0

1067 X 7 0

1068 X 8 3842.6 F-Q-P-H-P-G-L-Q-K-T-L-E-Q-F-H-L-S-S-M-S-S-
L-G-G-P-A-A-F-S-A-R-W-A-Q-E
1069 X 9 3842.6 F-Q-P-H-P-G-L-Q-K-T-L-E-Q-F-H-L-S-S-M-S-S-
L-G-G-P-A-A-F-S-A-R-W-A-Q-E
1070 X10 3842.6 F-Q-P-H-P-G-L-Q-K-T-L-E-Q-F-H-L-S-S-M-S-S-
L-G-G-P-A-A-F-S-A-R-W-A-Q-E
1071 X11 3842.6 F-Q-P-H-P-G-L-Q-K-T-L-E-Q-F-H-L-S-S-M-S-S-
L-G-G-P-A-A-F-S-A-R-W-A-Q-E
1072 X12 3842.6 F-Q-P-H-P-G-L-Q-K-T-L-E-Q-F-H-L-S-S-M-S-S-
L-G-G-P-A-A-F-S-A-R-W-A-Q-E
1073 X13 0

1074 X14 0

1075 X15 3842.6 F-Q-P-H-P-G-L-Q-K-T-L-E-Q-F-H-L-S-S-M-S-S-
L-G-G-P-A-A-F-S-A-R-W-A-Q-E
1076 X16 3842.6 F-Q-P-H-P-G-L-Q-K-T-L-E-Q-F-H-L-S-S-M-S-S-
L-G-G-P-A-A-F-S-A-R-W-A-Q-E
1077 X17 3842.6 F-Q-P-H-P-G-L-Q-K-T-L-E-Q-F-H-L-S-S-M-S-S-
L-G-G-P-A-A-F-S-A-R-W-A-Q-E
1078 X18 3842.6 F-Q-P-H-P-G-L-Q-K-T-L-E-Q-F-H-L-S-S-M-S-S-

L-G-G-P-A-A-F-S-A-R-W-A-Q-E
1079 X19 3842.6 F-Q-P-H-P-G-L-Q-K-T-L-E-Q-F-H-L-S-S-M-S-S-L-G-G-P-A-A-F-S-A-R-W-A-Q-E
1080 X20 0

1081 Y 1 3842.6 F-Q-P-H-P-G-L-Q-K-T-L-E-Q-F-H-L-S-S-M-S-S-L-G-G-P-A-A-F-S-A-R-W-A-Q-E
1082 Y 2 3842.6 F-Q-P-H-P-G-L-Q-K-T-L-E-Q-F-H-L-S-S-M-S-S-L-G-G-P-A-A-F-S-A-R-W-A-Q-E
1083 Y 3 3842.6 F-Q-P-H-P-G-L-Q-K-T-L-E-Q-F-H-L-S-S-M-S-S-L-G-G-P-A-A-F-S-A-R-W-A-Q-E
1084 Y 4 3842.6 F-Q-P-H-P-G-L-Q-K-T-L-E-Q-F-H-L-S-S-M-S-S-L-G-G-P-A-A-F-S-A-R-W-A-Q-E
1085 Y 5 3842.6 F-Q-P-H-P-G-L-Q-K-T-L-E-Q-F-H-L-S-S-M-S-S-L-G-G-P-A-A-F-S-A-R-W-A-Q-E
1086 Y 6 0

1087 Y 7 0

1088 Y 8 3842.6 F-Q-P-H-P-G-L-Q-K-T-L-E-Q-F-H-L-S-S-M-S-S-L-G-G-P-A-A-F-S-A-R-W-A-Q-E
1089 Y 9 3842.6 F-Q-P-H-P-G-L-Q-K-T-L-E-Q-F-H-L-S-S-M-S-S-L-G-G-P-A-A-F-S-A-R-W-A-Q-E
1090 Y10 3842.6 F-Q-P-H-P-G-L-Q-K-T-L-E-Q-F-H-L-S-S-M-S-S-L-G-G-P-A-A-F-S-A-R-W-A-Q-E
1091 Y11 3842.6 F-Q-P-H-P-G-L-Q-K-T-L-E-Q-F-H-L-S-S-M-S-S-L-G-G-P-A-A-F-S-A-R-W-A-Q-E
1092 Y12 3842.6 F-Q-P-H-P-G-L-Q-K-T-L-E-Q-F-H-L-S-S-M-S-S-L-G-G-P-A-A-F-S-A-R-W-A-Q-E
1093 Y13 0

1094 Y14 0

1095 Y15 3842.6 F-Q-P-H-P-G-L-Q-K-T-L-E-Q-F-H-L-S-S-M-S-S-L-G-G-P-A-A-F-S-A-R-W-A-Q-E
1096 Y16 3842.6 F-Q-P-H-P-G-L-Q-K-T-L-E-Q-F-H-L-S-S-M-S-S-L-G-G-P-A-A-F-S-A-R-W-A-Q-E
1097 Y17 3842.6 F-Q-P-H-P-G-L-Q-K-T-L-E-Q-F-H-L-S-S-M-S-S-L-G-G-P-A-A-F-S-A-R-W-A-Q-E
1098 Y18 3842.6 F-Q-P-H-P-G-L-Q-K-T-L-E-Q-F-H-L-S-S-M-S-S-L-G-G-P-A-A-F-S-A-R-W-A-Q-E
1099 Y19 3842.6 F-Q-P-H-P-G-L-Q-K-T-L-E-Q-F-H-L-S-S-M-S-S-L-G-G-P-A-A-F-S-A-R-W-A-Q-E
1100 Y20 0

1101 Z 1 0

1102 Z 2 0

1103 Z 3 0

1104 Z 4 0

1105 Z 5 0

1106 Z 6 0

1107 Z 7 0

1108 Z 8 0

1109 Z 9 0

1110 Z10 0

1111 Z11 0

1112 Z12 0

1113 Z13 0

1114 Z14 0

1115 Z15 0

1116 Z16 0

1117 Z17 0

1118 Z18 0

1119 Z19 0

1120 Z20 0

1121 [1 3516.3 T-L-E-Q-F-H-L-S-S-M-S-S-L-G-G-P-A-A-
F-S-A-R-W-A-Q-E-A-Y-K-K-E-S
1122 [2 3516.3 T-L-E-Q-F-H-L-S-S-M-S-S-L-G-G-P-A-A-
F-S-A-R-W-A-Q-E-A-Y-K-K-E-S
1123 [3 3516.3 T-L-E-Q-F-H-L-S-S-M-S-S-L-G-G-P-A-A-
F-S-A-R-W-A-Q-E-A-Y-K-K-E-S
1124 [4 3516.3 T-L-E-Q-F-H-L-S-S-M-S-S-L-G-G-P-A-A-
F-S-A-R-W-A-Q-E-A-Y-K-K-E-S
1125 [5 3516.3 T-L-E-Q-F-H-L-S-S-M-S-S-L-G-G-P-A-A-
F-S-A-R-W-A-Q-E-A-Y-K-K-E-S
1126 [6 0

1127 [7 0

1128 [8 3516.3 T-L-E-Q-F-H-L-S-S-M-S-S-L-G-G-P-A-A-
F-S-A-R-W-A-Q-E-A-Y-K-K-E-S
1129 [9 3516.3 T-L-E-Q-F-H-L-S-S-M-S-S-L-G-G-P-A-A-
F-S-A-R-W-A-Q-E-A-Y-K-K-E-S
1130 [10 3516.3 T-L-E-Q-F-H-L-S-S-M-S-S-L-G-G-P-A-A-
F-S-A-R-W-A-Q-E-A-Y-K-K-E-S
1131 [11 3516.3 T-L-E-Q-F-H-L-S-S-M-S-S-L-G-G-P-A-A-
F-S-A-R-W-A-Q-E-A-Y-K-K-E-S
1132 [12 3516.3 T-L-E-Q-F-H-L-S-S-M-S-S-L-G-G-P-A-A-

F-S-A-R-W-A-Q-E-A-Y-K-K-E-S
1133 [13 0

1134 [14 0

1135 [15 3516.3 T-L-E-Q-F-H-L-S-S-M-S-S-L-G-G-P-A-A-
F-S-A-R-W-A-Q-E-A-Y-K-K-E-S
1136 [16 3516.3 T-L-E-Q-F-H-L-S-S-M-S-S-L-G-G-P-A-A-
F-S-A-R-W-A-Q-E-A-Y-K-K-E-S
1137 [17 3516.3 T-L-E-Q-F-H-L-S-S-M-S-S-L-G-G-P-A-A-
F-S-A-R-W-A-Q-E-A-Y-K-K-E-S
1138 [18 3516.3 T-L-E-Q-F-H-L-S-S-M-S-S-L-G-G-P-A-A-
F-S-A-R-W-A-Q-E-A-Y-K-K-E-S
1139 [19 3516.3 T-L-E-Q-F-H-L-S-S-M-S-S-L-G-G-P-A-A-
F-S-A-R-W-A-Q-E-A-Y-K-K-E-S
1140 [20 0

1141 \ 1 3516.3 T-L-E-Q-F-H-L-S-S-M-S-S-L-G-G-P-A-A-
F-S-A-R-W-A-Q-E-A-Y-K-K-E-S
1142 \ 2 3516.3 T-L-E-Q-F-H-L-S-S-M-S-S-L-G-G-P-A-A-
F-S-A-R-W-A-Q-E-A-Y-K-K-E-S
1143 \ 3 3516.3 T-L-E-Q-F-H-L-S-S-M-S-S-L-G-G-P-A-A-
F-S-A-R-W-A-Q-E-A-Y-K-K-E-S
1144 \ 4 3516.3 T-L-E-Q-F-H-L-S-S-M-S-S-L-G-G-P-A-A-
F-S-A-R-W-A-Q-E-A-Y-K-K-E-S
1145 \ 5 3516.3 T-L-E-Q-F-H-L-S-S-M-S-S-L-G-G-P-A-A-
F-S-A-R-W-A-Q-E-A-Y-K-K-E-S
1146 \ 6 0

1147 \ 7 0

1148 \ 8 3516.3 T-L-E-Q-F-H-L-S-S-M-S-S-L-G-G-P-A-A-
F-S-A-R-W-A-Q-E-A-Y-K-K-E-S
1149 \ 9 3516.3 T-L-E-Q-F-H-L-S-S-M-S-S-L-G-G-P-A-A-
F-S-A-R-W-A-Q-E-A-Y-K-K-E-S
1150 \10 3516.3 T-L-E-Q-F-H-L-S-S-M-S-S-L-G-G-P-A-A-
F-S-A-R-W-A-Q-E-A-Y-K-K-E-S
1151 \11 3516.3 T-L-E-Q-F-H-L-S-S-M-S-S-L-G-G-P-A-A-
F-S-A-R-W-A-Q-E-A-Y-K-K-E-S
1152 \12 3516.3 T-L-E-Q-F-H-L-S-S-M-S-S-L-G-G-P-A-A-
F-S-A-R-W-A-Q-E-A-Y-K-K-E-S
1153 \13 0

1154 \14 0

1155 \15 3516.3 T-L-E-Q-F-H-L-S-S-M-S-S-L-G-G-P-A-A-
F-S-A-R-W-A-Q-E-A-Y-K-K-E-S
1156 \16 3516.3 T-L-E-Q-F-H-L-S-S-M-S-S-L-G-G-P-A-A-
F-S-A-R-W-A-Q-E-A-Y-K-K-E-S
1157 \17 3516.3 T-L-E-Q-F-H-L-S-S-M-S-S-L-G-G-P-A-A-
F-S-A-R-W-A-Q-E-A-Y-K-K-E-S
1158 \18 3516.3 T-L-E-Q-F-H-L-S-S-M-S-S-L-G-G-P-A-A-
F-S-A-R-W-A-Q-E-A-Y-K-K-E-S
1159 \19 3516.3 T-L-E-Q-F-H-L-S-S-M-S-S-L-G-G-P-A-A-

F-S-A-R-W-A-Q-E-A-Y-K-K-E-S
1160 \20 0

1161] 1 3516.3 T-L-E-Q-F-H-L-S-S-M-S-S-L-G-G-P-A-A-
F-S-A-R-W-A-Q-E-A-Y-K-K-E-S
1162] 2 3516.3 T-L-E-Q-F-H-L-S-S-M-S-S-L-G-G-P-A-A-
F-S-A-R-W-A-Q-E-A-Y-K-K-E-S
1163] 3 3516.3 T-L-E-Q-F-H-L-S-S-M-S-S-L-G-G-P-A-A-
F-S-A-R-W-A-Q-E-A-Y-K-K-E-S
1164] 4 3516.3 T-L-E-Q-F-H-L-S-S-M-S-S-L-G-G-P-A-A-
F-S-A-R-W-A-Q-E-A-Y-K-K-E-S
1165] 5 3516.3 T-L-E-Q-F-H-L-S-S-M-S-S-L-G-G-P-A-A-
F-S-A-R-W-A-Q-E-A-Y-K-K-E-S
1166] 6 0

1167] 7 0

1168] 8 3516.3 T-L-E-Q-F-H-L-S-S-M-S-S-L-G-G-P-A-A-
F-S-A-R-W-A-Q-E-A-Y-K-K-E-S
1169] 9 3516.3 T-L-E-Q-F-H-L-S-S-M-S-S-L-G-G-P-A-A-
F-S-A-R-W-A-Q-E-A-Y-K-K-E-S
1170]10 3516.3 T-L-E-Q-F-H-L-S-S-M-S-S-L-G-G-P-A-A-
F-S-A-R-W-A-Q-E-A-Y-K-K-E-S
1171]11 3516.3 T-L-E-Q-F-H-L-S-S-M-S-S-L-G-G-P-A-A-
F-S-A-R-W-A-Q-E-A-Y-K-K-E-S
1172]12 3516.3 T-L-E-Q-F-H-L-S-S-M-S-S-L-G-G-P-A-A-
F-S-A-R-W-A-Q-E-A-Y-K-K-E-S
1173]13 0

1174]14 0

1175]15 3516.3 T-L-E-Q-F-H-L-S-S-M-S-S-L-G-G-P-A-A-
F-S-A-R-W-A-Q-E-A-Y-K-K-E-S
1176]16 3516.3 T-L-E-Q-F-H-L-S-S-M-S-S-L-G-G-P-A-A-
F-S-A-R-W-A-Q-E-A-Y-K-K-E-S
1177]17 3516.3 T-L-E-Q-F-H-L-S-S-M-S-S-L-G-G-P-A-A-
F-S-A-R-W-A-Q-E-A-Y-K-K-E-S
1178]18 3516.3 T-L-E-Q-F-H-L-S-S-M-S-S-L-G-G-P-A-A-
F-S-A-R-W-A-Q-E-A-Y-K-K-E-S
1179]19 3516.3 T-L-E-Q-F-H-L-S-S-M-S-S-L-G-G-P-A-A-
F-S-A-R-W-A-Q-E-A-Y-K-K-E-S
1180]20 0

1181 ^ 1 3516.3 T-L-E-Q-F-H-L-S-S-M-S-S-L-G-G-P-A-A-
F-S-A-R-W-A-Q-E-A-Y-K-K-E-S
1182 ^ 2 3516.3 T-L-E-Q-F-H-L-S-S-M-S-S-L-G-G-P-A-A-
F-S-A-R-W-A-Q-E-A-Y-K-K-E-S
1183 ^ 3 3516.3 T-L-E-Q-F-H-L-S-S-M-S-S-L-G-G-P-A-A-
F-S-A-R-W-A-Q-E-A-Y-K-K-E-S
1184 ^ 4 3516.3 T-L-E-Q-F-H-L-S-S-M-S-S-L-G-G-P-A-A-
F-S-A-R-W-A-Q-E-A-Y-K-K-E-S
1185 ^ 5 3516.3 T-L-E-Q-F-H-L-S-S-M-S-S-L-G-G-P-A-A-
F-S-A-R-W-A-Q-E-A-Y-K-K-E-S
1186 ^ 6 0

1187 ^ 7 0

1188 ^ 8 3516.3 T-L-E-Q-F-H-L-S-S-M-S-S-L-G-G-P-A-A-
F-S-A-R-W-A-Q-E-A-Y-K-K-E-S
1189 ^ 9 3516.3 T-L-E-Q-F-H-L-S-S-M-S-S-L-G-G-P-A-A-
F-S-A-R-W-A-Q-E-A-Y-K-K-E-S
1190 ^10 3516.3 T-L-E-Q-F-H-L-S-S-M-S-S-L-G-G-P-A-A-
F-S-A-R-W-A-Q-E-A-Y-K-K-E-S
1191 ^11 3516.3 T-L-E-Q-F-H-L-S-S-M-S-S-L-G-G-P-A-A-
F-S-A-R-W-A-Q-E-A-Y-K-K-E-S
1192 ^12 3516.3 T-L-E-Q-F-H-L-S-S-M-S-S-L-G-G-P-A-A-
F-S-A-R-W-A-Q-E-A-Y-K-K-E-S
1193 ^13 0

1194 ^14 0

1195 ^15 3516.3 T-L-E-Q-F-H-L-S-S-M-S-S-L-G-G-P-A-A-
F-S-A-R-W-A-Q-E-A-Y-K-K-E-S
1196 ^16 3516.3 T-L-E-Q-F-H-L-S-S-M-S-S-L-G-G-P-A-A-
F-S-A-R-W-A-Q-E-A-Y-K-K-E-S
1197 ^17 3516.3 T-L-E-Q-F-H-L-S-S-M-S-S-L-G-G-P-A-A-
F-S-A-R-W-A-Q-E-A-Y-K-K-E-S
1198 ^18 3516.3 T-L-E-Q-F-H-L-S-S-M-S-S-L-G-G-P-A-A-
F-S-A-R-W-A-Q-E-A-Y-K-K-E-S
1199 ^19 3516.3 T-L-E-Q-F-H-L-S-S-M-S-S-L-G-G-P-A-A-
F-S-A-R-W-A-Q-E-A-Y-K-K-E-S

Array 1

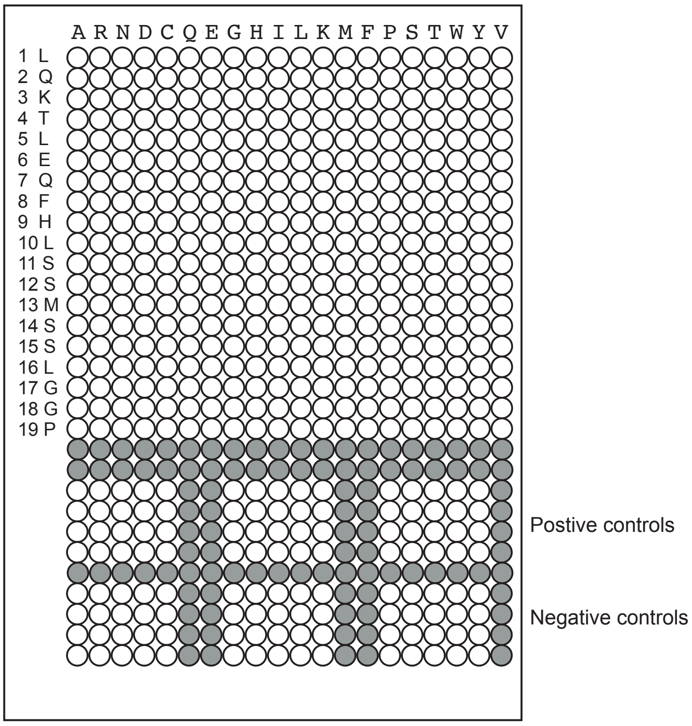

Array 2

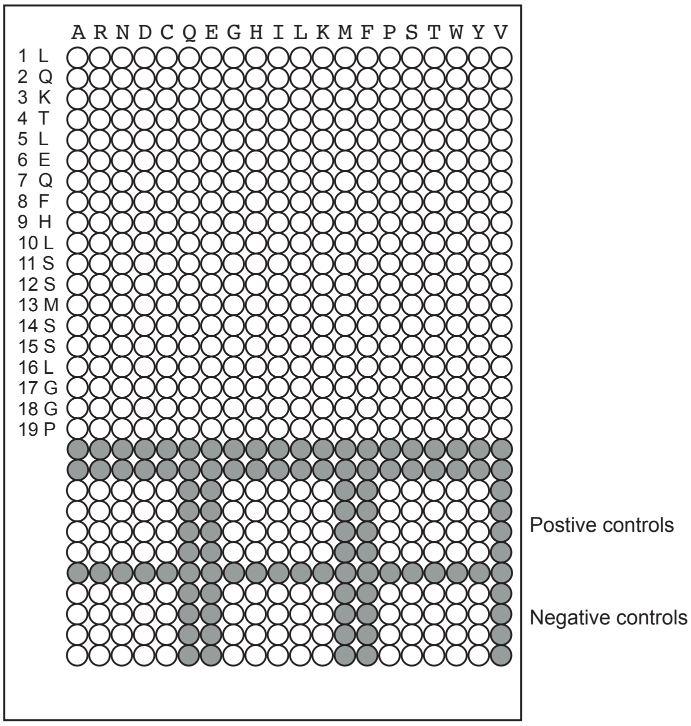

Supplement: Figure 4—source data 1. [file elife-63545-fig4-data1.pdf]
