# Supplementary material for: Mutations in SKI in Shprintzen–Goldberg syndrome lead to attenuated TGF-β responses through SKI stabilization
Source: eLife. 2021 Jan 8;10:e63545. doi: 10.7554/eLife.63545 (PMC7834018; doi:10.7554/eLife.63545)
Supplement: Figure 5—source data 1. [file elife-63545-fig5-data1.pdf]

Full wwPDB X-ray Structure Validation Report (i)

Jul 29, 2020 – 02:19 PM BST

PDB ID	:	6ZVQ
Title	:	Complex between SMAD2 MH2 domain and peptide from Ski corepressor
Deposited on	:	2020-07-27
Resolution	:	2.03 Å(reported)

This is a Full wwPDB X-ray Structure Validation Report.

This report is produced by the wwPDB biocuration pipeline after annotation of the structure.

We welcome your comments at *validation@mail.wwpdb.org*
A user guide is available at
https://www.wwpdb.org/validation/2017/XrayValidationReportHelp
with specific help available everywhere you see the (i) symbol.

CONFIDENTIAL VALIDATION REPORT

The following versions of software and data (see references (i)) were used in the production of this report:

MolProbity	:	4.02b-467
Mogul	:	1.8.5 (274361), CSD as541be (2020)
Xtriage (Phenix)	:	1.13
EDS	:	2.13
Percentile statistics	:	20191225.v01 (using entries in the PDB archive December 25th 2019)
Refmac	:	5.8.0158
CCP4	:	7.0.044 (Gargrove)
Ideal geometry (proteins)	:	Engh & Huber (2001)
Ideal geometry (DNA, RNA)	:	Parkinson et al. (1996)
Validation Pipeline (wwPDB-VP)	:	2.13

1 Overall quality at a glance (i)

The following experimental techniques were used to determine the structure:
X-RAY DIFFRACTION

The reported resolution of this entry is 2.03 Å.

Percentile scores (ranging between 0-100) for global validation metrics of the entry are shown in the following graphic. The table shows the number of entries on which the scores are based.

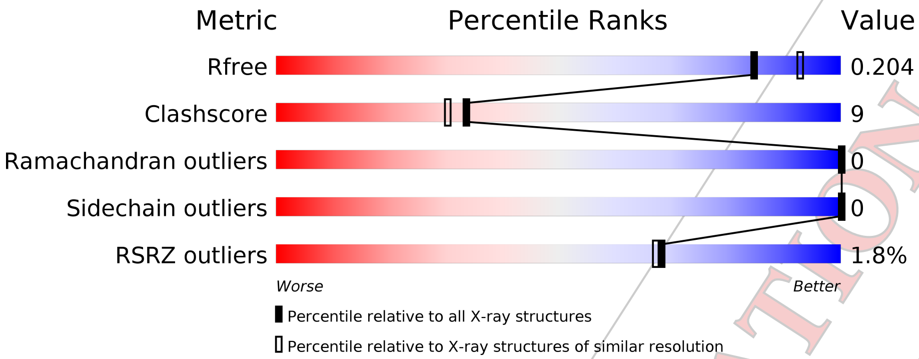

Metric	Whole archive (#Entries)	Similar resolution (#Entries, resolution range(Å))
R_{free}	130704	10434 (2.04-2.00)
Clashscore	141614	11643 (2.04-2.00)
Ramachandran outliers	138981	11493 (2.04-2.00)
Sidechain outliers	138945	11492 (2.04-2.00)
RSRZ outliers	127900	10220 (2.04-2.00)

The table below summarises the geometric issues observed across the polymeric chains and their fit to the electron density. The red, orange, yellow and green segments on the lower bar indicate the fraction of residues that contain outliers for >=3, 2, 1 and 0 types of geometric quality criteria respectively. A grey segment represents the fraction of residues that are not modelled. The numeric value for each fraction is indicated below the corresponding segment, with a dot representing fractions <=5% The upper red bar (where present) indicates the fraction of residues that have poor fit to the electron density. The numeric value is given above the bar.

Mol	Chain	Length	Quality of chain
1	A	227	75% 14% 11%
2	B	35	9%<br>66% 11% 23%

The following table lists non-polymeric compounds, carbohydrate monomers and non-standard residues in protein, DNA, RNA chains that are outliers for geometric or electron-density-fit criteria:

CONFIDENTIAL VALIDATION REPORT

Mol	Type	Chain	Res	Chirality	Geometry	Clashes	Electron density
5	TAR	A	508	X	-	-	-

CONFIDENTIAL VALIDATION REPORT

2 Entry composition (i)

There are 6 unique types of molecules in this entry. The entry contains 1971 atoms, of which 28 are hydrogens and 0 are deuteriums.

In the tables below, the ZeroOcc column contains the number of atoms modelled with zero occupancy, the AltConf column contains the number of residues with at least one atom in alternate conformation and the Trace column contains the number of residues modelled with at most 2 atoms.

- Molecule 1 is a protein called Mothers against decapentaplegic homolog 2.

Mol	Chain	Residues	Atoms						ZeroOcc	AltConf	Trace
			Total	C	N	O	P	S			
1	A	202	1601	1011	275	298	2	15	1	4	0

- Molecule 2 is a protein called Ski oncogene.

Mol	Chain	Residues	Atoms					ZeroOcc	AltConf	Trace
			Total	C	N	O	S			
2	B	27	178	114	30	33	1	0	0	0

- Molecule 3 is SULFATE ION (three-letter code: SO4) (formula: O_4S).

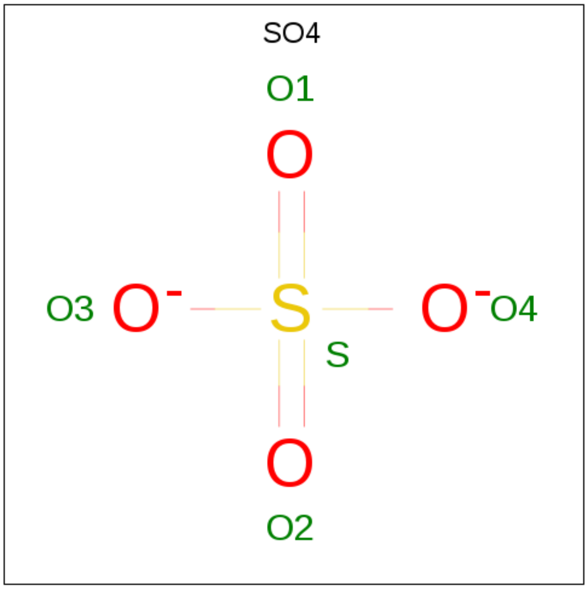

Mol	Chain	Residues	Atoms			ZeroOcc	AltConf
			Total	O	S		
3	A	1	5	4	1	0	0
3	A	1	5	4	1	0	0

CONFIDENTIAL VALIDATION REPORT

- Molecule 4 is GLYCEROL (three-letter code: GOL) (formula: $C_3H_8O_3$).

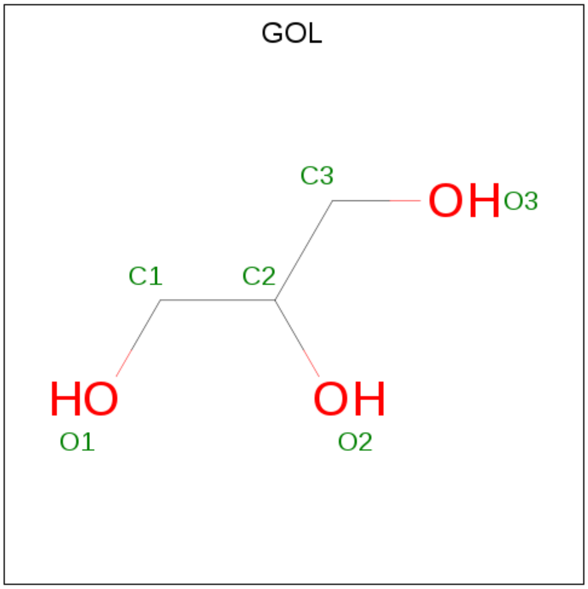

Mol	Chain	Residues	Atoms	ZeroOcc	AltConf
4	A	1	Total C O<br>6 3 3	0	0
4	A	1	Total C H O<br>14 3 8 3	0	0
4	A	1	Total C O<br>6 3 3	0	0
4	A	1	Total C O<br>6 3 3	0	0
4	A	1	Total C O<br>6 3 3	0	0
4	A	1	Total C O<br>6 3 3	0	0
4	A	1	Total C H O<br>14 3 8 3	0	0
4	A	1	Total C H O<br>14 3 8 3	0	0

- Molecule 5 is D(-)-TARTARIC ACID (three-letter code: TAR) (formula: $C_4H_6O_6$).

CONFIDENTIAL VALIDATION REPORT

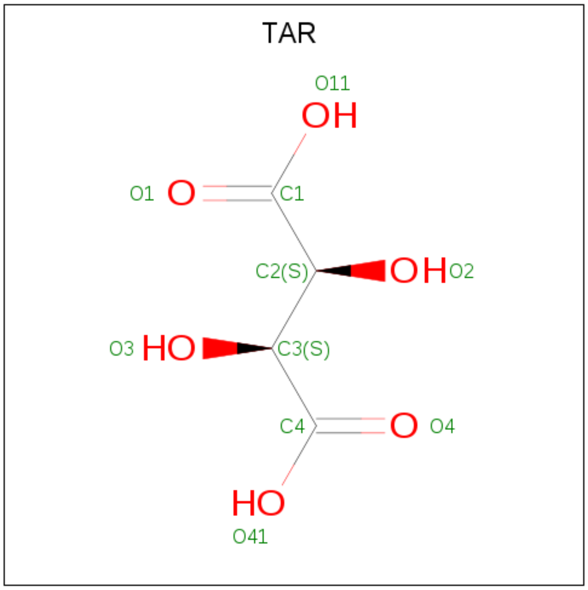

Mol	Chain	Residues	Atoms	ZeroOcc	AltConf
5	A	1	Total C O<br>10 4 6	0	0
5	A	1	Total C O<br>10 4 6	0	0
5	A	1	Total C H O<br>14 4 4 6	0	0

- Molecule 6 is water.

Mol	Chain	Residues	Atoms	ZeroOcc	AltConf
6	A	72	Total O<br>72 72	0	0
6	B	4	Total O<br>4 4	0	0

CONFIDENTIAL VALIDATION REPORT

3 Residue-property plots (i)

These plots are drawn for all protein, RNA, DNA and oligosaccharide chains in the entry. The first graphic for a chain summarises the proportions of the various outlier classes displayed in the second graphic. The second graphic shows the sequence view annotated by issues in geometry and electron density. Residues are color-coded according to the number of geometric quality criteria for which they contain at least one outlier: green = 0, yellow = 1, orange = 2 and red = 3 or more. A red dot above a residue indicates a poor fit to the electron density (RSRZ > 2). Stretches of 2 or more consecutive residues without any outlier are shown as a green connector. Residues present in the sample, but not in the model, are shown in grey.

- Molecule 1: Mothers against decapentaplegic homolog 2

Chain A:

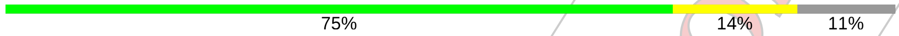

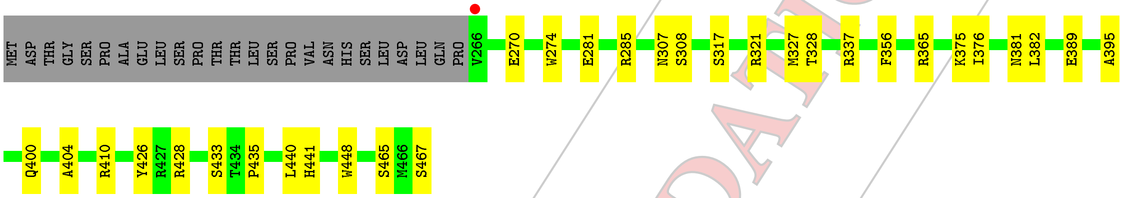

- Molecule 2: Ski oncogene

Chain B:

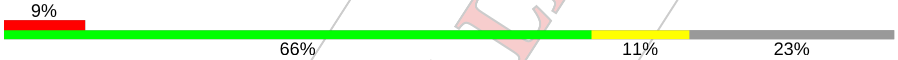

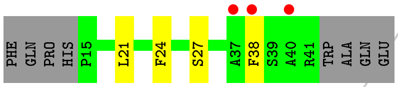

CONFIDENTIAL VALIDATION REPORT

4 Data and refinement statistics (i)

Property	Value	Source
Space group	I 21 3	Depositor
Cell constants<br>a, b, c, α, β, γ	113.82Å 113.82Å 113.82Å<br>90.00° 90.00° 90.00°	Depositor
Resolution (Å)	56.91 – 2.03<br>56.91 – 2.03	Depositor<br>EDS
% Data completeness<br>(in resolution range)	99.9 (56.91-2.03)<br>92.4 (56.91-2.03)	Depositor<br>EDS
R_{merge}	0.12	Depositor
R_{sym}	(Not available)	Depositor
$< I/\sigma(I) >$ [1]	1.14 (at 2.03Å)	Xtriage
Refinement program	REFMAC 5.8.0258, PHENIX 1.18.2_3874	Depositor
R, R_{free}	0.164 , 0.210<br>0.167 , 0.204	Depositor<br>DCC
R_{free} test set	744 reflections (4.66%)	wwPDB-VP
Wilson B-factor (Å^2)	33.1	Xtriage
Anisotropy	0.000	Xtriage
Bulk solvent k_{sol}(e/Å^3), B_{sol}(Å^2)	0.37 , 57.5	EDS
L-test for twinning[2]	$< \|L\| >$ = 0.49, $< L^2 >$ = 0.32	Xtriage
Estimated twinning fraction	0.045 for -l,-k,-h	Xtriage
F_o,F_c correlation	0.97	EDS
Total number of atoms	1971	wwPDB-VP
Average B, all atoms (Å^2)	43.0	wwPDB-VP

Xtriage's analysis on translational NCS is as follows: *The largest off-origin peak in the Patterson function is 3.90% of the height of the origin peak. No significant pseudotranslation is detected.*

[1] Intensities estimated from amplitudes.
[2] Theoretical values of $< |L| >$, $< L^2 >$ for acentric reflections are 0.5, 0.333 respectively for untwinned datasets, and 0.375, 0.2 for perfectly twinned datasets.

CONFIDENTIAL VALIDATION REPORT

5 Model quality (i)

5.1 Standard geometry (i)

Bond lengths and bond angles in the following residue types are not validated in this section: GOL, SO4, TAR, SEP

The Z score for a bond length (or angle) is the number of standard deviations the observed value is removed from the expected value. A bond length (or angle) with $|Z| > 5$ is considered an outlier worth inspection. RMSZ is the root-mean-square of all Z scores of the bond lengths (or angles).

Mol	Chain	Bond lengths		Bond angles	
		RMSZ	#$\|Z\|$ >5	RMSZ	#$\|Z\|$ >5
1	A	0.41	0/1633	0.59	0/2222
2	B	0.31	0/182	0.49	0/246
All	All	0.40	0/1815	0.58	0/2468

There are no bond length outliers.

There are no bond angle outliers.

There are no chirality outliers.

There are no planarity outliers.

5.2 Too-close contacts (i)

In the following table, the Non-H and H(model) columns list the number of non-hydrogen atoms and hydrogen atoms in the chain respectively. The H(added) column lists the number of hydrogen atoms added and optimized by MolProbity. The Clashes column lists the number of clashes within the asymmetric unit, whereas Symm-Clashes lists symmetry related clashes.

Mol	Chain	Non-H	H(model)	H(added)	Clashes	Symm-Clashes
1	A	1601	0	1531	28	0
2	B	178	0	161	4	0
3	A	10	0	0	0	0
4	A	48	24	64	12	0
5	A	30	4	12	4	0
6	A	72	0	0	0	0
6	B	4	0	0	0	0
All	All	1943	28	1768	31	0

The all-atom clashscore is defined as the number of clashes found per 1000 atoms (including hydrogen atoms). The all-atom clashscore for this structure is 9.

CONFIDENTIAL VALIDATION REPORT

All (31) close contacts within the same asymmetric unit are listed below, sorted by their clash magnitude.

Atom-1	Atom-2	Interatomic distance (Å)	Clash overlap (Å)
1:A:337:ARG:HD3	4:A:505:GOL:H12	1.51	0.92
2:B:27:SER:HB2	2:B:38:PHE:CD2	2.12	0.83
2:B:27:SER:HB2	2:B:38:PHE:HD2	1.48	0.79
1:A:376[A]:ILE:HD11	1:A:382:LEU:HD22	1.65	0.78
1:A:410:ARG:HG2	5:A:508:TAR:O3	1.88	0.72
1:A:337:ARG:CD	4:A:505:GOL:H12	2.20	0.70
1:A:307:ASN:HA	4:A:506:GOL:H31	1.76	0.66
1:A:308:SER:HB3	4:A:506:GOL:H2	1.78	0.65
1:A:337:ARG:HD3	4:A:505:GOL:C1	2.24	0.65
1:A:274:TRP:HB3	4:A:509:GOL:H31	1.78	0.64
1:A:270:GLU:OE1	4:A:509:GOL:O2	2.15	0.62
1:A:356:PHE:CE2	1:A:375:LYS:HG3	2.35	0.62
1:A:274:TRP:CB	4:A:509:GOL:H31	2.31	0.60
1:A:395:ALA:HA	2:B:21:LEU:HD22	1.84	0.59
1:A:356:PHE:HE2	1:A:426:TYR:HH	1.53	0.55
1:A:321:ARG:HH11	4:A:504:GOL:H31	1.72	0.55
1:A:365:ARG:HG3	1:A:389:GLU:OE2	2.12	0.49
1:A:321:ARG:NH1	4:A:504:GOL:H31	2.28	0.49
1:A:428:ARG:HB3	1:A:433[B]:SER:OG	2.14	0.47
1:A:308:SER:CB	4:A:506:GOL:H2	2.44	0.47
1:A:448:TRP:CE3	2:B:24:PHE:HB2	2.49	0.47
1:A:317:SER:O	4:A:510:GOL:H31	2.15	0.46
5:A:508:TAR:O3	5:A:508:TAR:O11	2.28	0.45
1:A:400:GLN:HB2	1:A:404:ALA:CB	2.46	0.45
1:A:327:MET:HG3	1:A:328:THR:N	2.31	0.45
1:A:381:ASN:O	5:A:507:TAR:H2	2.18	0.43
1:A:285:ARG:HG2	1:A:285:ARG:HH11	1.84	0.43
1:A:440:LEU:HD23	1:A:441:HIS:N	2.34	0.43
1:A:281:GLU:HA	1:A:435:PRO:O	2.19	0.42
1:A:356:PHE:CE2	1:A:375:LYS:CG	3.01	0.42
1:A:410:ARG:CG	5:A:508:TAR:O3	2.63	0.40

There are no symmetry-related clashes.

CONFIDENTIAL VALIDATION REPORT

5.3 Torsion angles (i)

5.3.1 Protein backbone (i)

In the following table, the Percentiles column shows the percent Ramachandran outliers of the chain as a percentile score with respect to all X-ray entries followed by that with respect to entries of similar resolution.

The Analysed column shows the number of residues for which the backbone conformation was analysed, and the total number of residues.

Mol	Chain	Analysed	Favoured	Allowed	Outliers	Percentiles	
1	A	203/227 (89%)	196 (97%)	7 (3%)	0	100	100
2	B	25/35 (71%)	22 (88%)	3 (12%)	0	100	100
All	All	228/262 (87%)	218 (96%)	10 (4%)	0	100	100

There are no Ramachandran outliers to report.

5.3.2 Protein sidechains (i)

In the following table, the Percentiles column shows the percent sidechain outliers of the chain as a percentile score with respect to all X-ray entries followed by that with respect to entries of similar resolution.

The Analysed column shows the number of residues for which the sidechain conformation was analysed, and the total number of residues.

Mol	Chain	Analysed	Rotameric	Outliers	Percentiles	
1	A	172/197 (87%)	172 (100%)	0	100	100
2	B	16/28 (57%)	16 (100%)	0	100	100
All	All	188/225 (84%)	188 (100%)	0	100	100

There are no protein residues with a non-rotameric sidechain to report.

Some sidechains can be flipped to improve hydrogen bonding and reduce clashes. All (2) such sidechains are listed below:

Mol	Chain	Res	Type
1	A	455	GLN
2	B	25	HIS

CONFIDENTIAL VALIDATION REPORT

5.3.3 RNA (i)

There are no RNA molecules in this entry.

5.4 Non-standard residues in protein, DNA, RNA chains (i)

2 non-standard protein/DNA/RNA residues are modelled in this entry.

In the following table, the Counts columns list the number of bonds (or angles) for which Mogul statistics could be retrieved, the number of bonds (or angles) that are observed in the model and the number of bonds (or angles) that are defined in the Chemical Component Dictionary. The Link column lists molecule types, if any, to which the group is linked. The Z score for a bond length (or angle) is the number of standard deviations the observed value is removed from the expected value. A bond length (or angle) with $|Z| > 2$ is considered an outlier worth inspection. RMSZ is the root-mean-square of all Z scores of the bond lengths (or angles).

Mol	Type	Chain	Res	Link	Bond lengths			Bond angles		
					Counts	RMSZ	$\#\|Z\| > 2$	Counts	RMSZ	$\#\|Z\| > 2$
1	SEP	A	467	1	7,10,10	1.61	1 (14%)	8,14,14	1.48	2 (25%)
1	SEP	A	465	1	8,9,10	1.52	1 (12%)	8,12,14	0.92	0

In the following table, the Chirals column lists the number of chiral outliers, the number of chiral centers analysed, the number of these observed in the model and the number defined in the Chemical Component Dictionary. Similar counts are reported in the Torsion and Rings columns. '-' means no outliers of that kind were identified.

Mol	Type	Chain	Res	Link	Chirals	Torsions	Rings
1	SEP	A	467	1	-	0/6/10/10	-
1	SEP	A	465	1	-	0/5/8/10	-

All (2) bond length outliers are listed below:

Mol	Chain	Res	Type	Atoms	Z	Observed(Å)	Ideal(Å)
1	A	467	SEP	P-O1P	3.43	1.61	1.50
1	A	465	SEP	P-O1P	3.35	1.61	1.50

All (2) bond angle outliers are listed below:

Mol	Chain	Res	Type	Atoms	Z	Observed(^{o})	Ideal(^{o})
1	A	467	SEP	OG-CB-CA	2.84	110.53	108.06
1	A	467	SEP	P-OG-CB	-2.09	112.53	118.30

There are no chirality outliers.

CONFIDENTIAL VALIDATION REPORT

There are no torsion outliers.

There are no ring outliers.

No monomer is involved in short contacts.

5.5 Carbohydrates (i)

There are no monosaccharides in this entry.

5.6 Ligand geometry (i)

13 ligands are modelled in this entry.

In the following table, the Counts columns list the number of bonds (or angles) for which Mogul statistics could be retrieved, the number of bonds (or angles) that are observed in the model and the number of bonds (or angles) that are defined in the Chemical Component Dictionary. The Link column lists molecule types, if any, to which the group is linked. The Z score for a bond length (or angle) is the number of standard deviations the observed value is removed from the expected value. A bond length (or angle) with $|Z| > 2$ is considered an outlier worth inspection. RMSZ is the root-mean-square of all Z scores of the bond lengths (or angles).

Mol	Type	Chain	Res	Link	Bond lengths			Bond angles		
					Counts	RMSZ	$\#\|Z\| > 2$	Counts	RMSZ	$\#\|Z\| > 2$
4	GOL	A	509	-	5,5,5	1.05	0	5,5,5	0.92	0
5	TAR	A	513	-	3,9,9	0.33	0	6,12,12	0.95	1 (16%)
4	GOL	A	506	-	5,5,5	1.03	0	5,5,5	0.87	0
4	GOL	A	512	-	5,5,5	0.82	0	5,5,5	1.09	0
4	GOL	A	505	-	5,5,5	0.82	0	5,5,5	1.01	0
5	TAR	A	508	-	3,9,9	0.44	0	6,12,12	0.70	0
5	TAR	A	507	-	3,9,9	0.33	0	6,12,12	0.66	0
3	SO4	A	502	-	4,4,4	0.16	0	6,6,6	0.16	0
4	GOL	A	503	-	5,5,5	0.89	0	5,5,5	1.00	0
4	GOL	A	504	-	5,5,5	0.75	0	5,5,5	0.94	0
4	GOL	A	510	-	5,5,5	0.89	0	5,5,5	1.02	0
3	SO4	A	501	-	4,4,4	0.16	0	6,6,6	0.11	0
4	GOL	A	511	-	5,5,5	0.70	0	5,5,5	0.93	0

In the following table, the Chirals column lists the number of chiral outliers, the number of chiral centers analysed, the number of these observed in the model and the number defined in the Chemical Component Dictionary. Similar counts are reported in the Torsion and Rings columns. '-' means no outliers of that kind were identified.

Mol	Type	Chain	Res	Link	Chirals	Torsions	Rings
4	GOL	A	509	-	-	2/4/4/4	-

Continued on next page...

CONFIDENTIAL VALIDATION REPORT

Continued from previous page...

Mol	Type	Chain	Res	Link	Chirals	Torsions	Rings
5	TAR	A	513	-	-	4/4/12/12	-
4	GOL	A	506	-	-	0/4/4/4	-
4	GOL	A	505	-	-	2/4/4/4	-
4	GOL	A	512	-	-	0/4/4/4	-
5	TAR	A	508	-	1/1/4/4	4/4/12/12	-
5	TAR	A	507	-	-	4/4/12/12	-
4	GOL	A	503	-	-	1/4/4/4	-
4	GOL	A	504	-	-	2/4/4/4	-
4	GOL	A	510	-	-	1/4/4/4	-
4	GOL	A	511	-	-	2/4/4/4	-

There are no bond length outliers.

All (1) bond angle outliers are listed below:

Mol	Chain	Res	Type	Atoms	Z	Observed(°)	Ideal(°)
5	A	513	TAR	C1-C2-C3	-2.12	108.55	113.11

All (1) chirality outliers are listed below:

Mol	Chain	Res	Type	Atom
5	A	508	TAR	C2

All (22) torsion outliers are listed below:

Mol	Chain	Res	Type	Atoms
4	A	509	GOL	O1-C1-C2-C3
5	A	513	TAR	C1-C2-C3-C4
5	A	508	TAR	C1-C2-C3-C4
5	A	508	TAR	O2-C2-C3-C4
5	A	507	TAR	C1-C2-C3-C4
4	A	504	GOL	C1-C2-C3-O3
4	A	504	GOL	O2-C2-C3-O3
4	A	511	GOL	O1-C1-C2-C3
4	A	505	GOL	O1-C1-C2-C3
5	A	513	TAR	C1-C2-C3-O3
5	A	513	TAR	O2-C2-C3-C4
4	A	509	GOL	O1-C1-C2-O2
4	A	505	GOL	O1-C1-C2-O2
4	A	511	GOL	O1-C1-C2-O2
5	A	513	TAR	O2-C2-C3-O3

Continued on next page...

CONFIDENTIAL VALIDATION REPORT

Continued from previous page...

Mol	Chain	Res	Type	Atoms
5	A	508	TAR	O2-C2-C3-O3
5	A	507	TAR	O2-C2-C3-C4
5	A	508	TAR	C1-C2-C3-O3
5	A	507	TAR	C1-C2-C3-O3
4	A	510	GOL	O1-C1-C2-C3
5	A	507	TAR	O2-C2-C3-O3
4	A	503	GOL	C1-C2-C3-O3

There are no ring outliers.

7 monomers are involved in 16 short contacts:

Mol	Chain	Res	Type	Clashes	Symm-Clashes
4	A	509	GOL	3	0
4	A	506	GOL	3	0
4	A	505	GOL	3	0
5	A	508	TAR	3	0
5	A	507	TAR	1	0
4	A	504	GOL	2	0
4	A	510	GOL	1	0

5.7 Other polymers (i)

There are no such residues in this entry.

5.8 Polymer linkage issues (i)

There are no chain breaks in this entry.

CONFIDENTIAL VALIDATION REPORT

6 Fit of model and data (i)

6.1 Protein, DNA and RNA chains (i)

In the following table, the column labelled '#RSRZ> 2' contains the number (and percentage) of RSRZ outliers, followed by percent RSRZ outliers for the chain as percentile scores relative to all X-ray entries and entries of similar resolution. The OWAB column contains the minimum, median, 95th percentile and maximum values of the occupancy-weighted average B-factor per residue. The column labelled 'Q< 0.9' lists the number of (and percentage) of residues with an average occupancy less than 0.9.

Mol	Chain	Analysed	<RSRZ>	#RSRZ>2			OWAB(Å^2)	Q<0.9
1	A	200/227 (88%)	-0.16	1 (0%)	91	91	27, 37, 55, 61	3 (1%)
2	B	27/35 (77%)	0.85	3 (11%)	5	5	50, 62, 87, 95	1 (3%)
All	All	227/262 (86%)	-0.04	4 (1%)	68	67	27, 39, 63, 95	4 (1%)

All (4) RSRZ outliers are listed below:

Mol	Chain	Res	Type	RSRZ
2	B	37	ALA	4.0
1	A	266	VAL	3.1
2	B	40	ALA	2.8
2	B	38	PHE	2.7

6.2 Non-standard residues in protein, DNA, RNA chains (i)

In the following table, the Atoms column lists the number of modelled atoms in the group and the number defined in the chemical component dictionary. The B-factors column lists the minimum, median, 95th percentile and maximum values of B factors of atoms in the group. The column labelled 'Q< 0.9' lists the number of atoms with occupancy less than 0.9.

Mol	Type	Chain	Res	Atoms	RSCC	RSR	B-factors(Å^2)	Q<0.9
1	SEP	A	467	11/11	0.96	0.10	50,51,54,54	0
1	SEP	A	465	10/11	0.98	0.09	44,46,50,52	0

6.3 Carbohydrates (i)

There are no monosaccharides in this entry.

CONFIDENTIAL VALIDATION REPORT

6.4 Ligands (i)

In the following table, the Atoms column lists the number of modelled atoms in the group and the number defined in the chemical component dictionary. The B-factors column lists the minimum, median, 95th percentile and maximum values of B factors of atoms in the group. The column labelled ‘Q< 0.9’ lists the number of atoms with occupancy less than 0.9.

Mol	Type	Chain	Res	Atoms	RSCC	RSR	B-factors($Å^2$)	Q<0.9
5	TAR	A	513	10/10	0.61	0.34	80,85,101,102	0
5	TAR	A	507	10/10	0.69	0.28	91,92,93,93	0
4	GOL	A	504	6/6	0.74	0.29	64,77,82,83	0
4	GOL	A	511	6/6	0.75	0.24	76,92,94,95	0
5	TAR	A	508	10/10	0.78	0.29	57,59,61,62	10
4	GOL	A	506	6/6	0.81	0.25	71,71,72,74	0
4	GOL	A	512	6/6	0.81	0.14	76,91,93,94	0
4	GOL	A	509	6/6	0.87	0.22	66,68,68,68	0
4	GOL	A	503	6/6	0.88	0.22	60,62,65,66	0
4	GOL	A	510	6/6	0.89	0.26	66,68,68,70	0
4	GOL	A	505	6/6	0.92	0.17	67,69,70,70	0
3	SO4	A	502	5/5	0.96	0.20	74,76,77,77	0
3	SO4	A	501	5/5	0.97	0.13	73,73,73,74	0

6.5 Other polymers (i)

There are no such residues in this entry.

CONFIDENTIAL VALIDATION REPORT
