# Supplementary material for: Mutations in SKI in Shprintzen–Goldberg syndrome lead to attenuated TGF-β responses through SKI stabilization
Source: eLife. 2021 Jan 8;10:e63545. doi: 10.7554/eLife.63545 (PMC7834018; doi:10.7554/eLife.63545)
Supplement: Transparent reporting form [file elife-63545-transrepform.docx]

***eLife’s* transparent reporting form**

We encourage authors to provide detailed information *within their submission* to facilitate the interpretation and replication of experiments. Authors can upload supporting documentation to indicate the use of appropriate reporting guidelines for health-related research (see [EQUATOR Network](http://www.equator-network.org/%20)), life science research (see the [BioSharing Information Resource](https://biosharing.org/)), or the [ARRIVE guidelines](http://www.plosbiology.org/article/info:doi/10.1371/journal.pbio.1000412) for reporting work involving animal research. Where applicable, authors should refer to any relevant reporting standards documents in this form.

If you have any questions, please consult our Journal Policies and/or contact us: [editorial@elifesciences.org](mailto:editorial@elifesciences.org).

**Sample-size estimation**

- You should state whether an appropriate sample size was computed when the study was being designed
- You should state the statistical method of sample size computation and any required assumptions
- If no explicit power analysis was used, you should describe how you decided what sample (replicate) size (number) to use

Please outline where this information can be found within the submission (e.g., sections or figure legends), or explain why this information doesn’t apply to your submission:

This does not apply to this work as the paper does not contain any population studies or *in vivo* experiments.

**Replicates**

- You should report how often each experiment was performed
- You should include a definition of biological versus technical replication
- The data obtained should be provided and sufficient information should be provided to indicate the number of independent biological and/or technical replicates
- If you encountered any outliers, you should describe how these were handled
- Criteria for exclusion/inclusion of data should be clearly stated
- High-throughput sequence data should be uploaded before submission, with a private link for reviewers provided (these are available from both GEO and ArrayExpress)

Please outline where this information can be found within the submission (e.g., sections or figure legends), or explain why this information doesn’t apply to your submission:

• As stated in the Methods, all experiments that were quantified were performed at least in triplicate as independent experiments (biological replicates) for statistical analysis unless otherwise specified in the figure legends.

- For qPCR at least four or more biological replicates (independent experiments) were performed in technical duplicates.

- For Luciferase assays three or more biological experiments (independent experiments) were performed with four technical replicates.

- For Flow Cytometry and Western blot quantification at least four biological replicates (independent experiments) were used.

- For peptide array experiments quantification was performed from two independent experiments.

• For all other experiments a representative of at least two biological independent replicates is shown.

• RNA sequencing experiments are the mean of four biological replicates. Validation of RNA seq data performed by qPCR analysis is the average of at least 4 biological experiments performed in technical duplicate.

• Criteria used for RNA-seq data analyses and information regarding the generation of the different datasets presented in the work are described in the Methods.

• RNA-seq data have been submitted to the European Genome-phenome Archive (EGA) under the accession number EGAS00001004908.

**Statistical reporting**

- Statistical analysis methods should be described and justified
- Raw data should be presented in figures whenever informative to do so (typically when N per group is less than 10)
- For each experiment, you should identify the statistical tests used, exact values of N, definitions of center, methods of multiple test correction, and dispersion and precision measures (e.g., mean, median, SD, SEM, confidence intervals; and, for the major substantive results, a measure of effect size (e.g., Pearson's r, Cohen's d)
- Report exact p-values wherever possible alongside the summary statistics and 95% confidence intervals. These should be reported for all key questions and not only when the p-value is less than 0.05.

Please outline where this information can be found within the submission (e.g., sections or figure legends), or explain why this information doesn’t apply to your submission:

As described in the Methods, for all experiments where data was quantified, the normalized values were log transformed for the statistical analysis. Statistical analysis was performed in Prism 8 (GraphPad). For comparison between more than two groups with one variable, one-way analysis of variance (Anova) was used followed by the Sidak’s correction test. For comparison between groups that had been split on two independent variables, two-way analysis of variance (Anova) was performed followed by Tukey’s multiple comparison tests.

(For large datasets, or papers with a very large number of statistical tests, you may upload a single table file with tests, Ns, etc., with reference to sections in the manuscript.)

**Group allocation**

- Indicate how samples were allocated into experimental groups (in the case of clinical studies, please specify allocation to treatment method); if randomization was used, please also state if restricted randomization was applied
- Indicate if masking was used during group allocation, data collection and/or data analysis

Please outline where this information can be found within the submission (e.g., sections or figure legends), or explain why this information doesn’t apply to your submission:

N/A

**Additional data files (“source data”)**

- We encourage you to upload relevant additional data files, such as numerical data that are represented as a graph in a figure, or as a summary table
- Where provided, these should be in the most useful format, and they can be uploaded as “Source data” files linked to a main figure or table
- Include model definition files including the full list of parameters used
- Include code used for data analysis (e.g., R, MatLab)
- Avoid stating that data files are “available upon request”

**Figure 1 – Source data 1**

Sequences of knockout alleles made in HEK293T cells.

**Figure 1 Supplement 1 – Source data 1**

Luciferase assay data for HEK293T S4 KO clones, as presented in Figure 1 Supplement 1A.

**Figure 1 Supplement 1 – Source data 2**

Luciferase assay data for HaCaT S4 KO clones, as presented in Figure 1 Supplement 1B.

**Figure 1 Supplement 1 – Source data 3**

qPCR data for HaCaT S4 KO clones, as presented in Figure 1 Supplement 1C

**Figure 2 – Source data 1**

Quantification of Western blot for HaCaT S4 KO rescue cell lines, as presented in Figure 2B

**Figure 2 – Source data 2**

Flow cytometry data for HaCaT S4 KO rescue cell lines, as presented in Figure 2C

**Figure 2 Supplement 1 – Source data 1**

Luciferase assays data for HaCaT S4 KO rescue cell lines, as presented in Figure 2 Supplement 1A

**Figure 2 Supplement 1 – Source data 2**

qPCR data for HaCaT S4 KO rescue cell lines, as presented in Figure 2 Supplement 1B

**Figure 4 – Source data 1**

Peptides sequences for peptide array.

**Figure 4 – Source data 2**

Quantification of peptide arrays.

**Figure 5 – Source data 1**

Structure Validation report for crystal structure (ID: 6ZVQ).

**Figure 6 – Source data 1**

Luciferase assays for Activin A-induced HEK293T P35S SKI clones, as presented in Figure 6D

**Figure 6 – Source data 2**

Luciferase assays for BMP4-induced HEK293T P35S SKI clones, as presented in Figure 6E.

**Figure 7 – Source data 1**

RNA-seq raw data

**Figure 7 Supplement 2 – Source data 1**

qPCR validations of RNA-seq data for SGS and control dermal fibroblasts.

Please indicate the figures or tables for which source data files have been provided:
